# Individual Differences in Perceived Complexity, Visual Discomfort, and Preference for Images Varying in Spectral Scaling

**DOI:** 10.3390/vision10040085

**Published:** 2026-10-21

**Authors:** Branka Spehar, Zoe Wilde

**Affiliations:** School of Psychology, UNSW Sydney, Sydney, NSW 2052, Australia; zoewildehk@gmail.com

**Keywords:** complexity, discomfort, preference, natural scene statistics, individual differences

## Abstract

Participants (*N* = 89) rated preference, discomfort, and perceived complexity of grayscale, thresholded, edge and mountain profile images varying in spectral scaling properties. Participants also completed self-report questionnaires designed to measure neurodivergent (CATI, ASRS) or sensory processing sensitivity (CHYPS-V, SPQ-S). Across image types, perceived complexity and discomfort declined as the spectral slope parameter increased, whereas preference increased from the shallowest to the intermediate slopes and then plateaued. Self-reported autistic-like traits, ADHD-like traits, and sensory sensitivity were largely unrelated to overall rating levels but showed small associations with how ratings changed across spectral slope. These findings support treating complexity, discomfort, and preference as related but non-equivalent visual judgments, with trait-related influences expressed mainly as modest variation in sensitivity to spectral scaling.

## 1. Background

Perceptual processes are accompanied by qualitative and affective properties that permeate our moment-to-moment experiences, influencing our moods, actions, and decisions, but they remain relatively less studied. A central limiting factor is that these affective properties of perception are often explored separately across a wide range of domains including esthetics, emotion and visual discomfort [1,2]. Furthermore, the domains such as esthetics and emotion processing are preoccupied with specialized stimuli such as artworks or scenes with explicit emotional content, and thus quite removed from basic perceptual processing [3,4]. Similarly, visual discomfort is often considered together with the broader notions of atypical sensory sensitivities (hyper and hyposensitivity) and has generally been relegated to clinical domains such as migraine and neurodivergent conditions, including autism and attention deficit hyperactivity disorder (ADHD) [5].

Despite these challenges, research over the past few decades has established close links between preference, visual discomfort, and low-level visual features associated with natural scene statistics. For example, images that deviate from natural scene statistics are consistently rated as less pleasant and more uncomfortable [2,6,7,8,9,10]. These findings build on the foundational work of Berlyne [11,12], who argued that affective responses are shaped by low-level stimulus properties and the degree of sensory arousal they evoke. If images conforming to natural scene statistics are optimally stimulating, or processed most efficiently, it follows that they should be both preferred and experienced as less uncomfortable than images that deviate from these statistics. Consistent with this prediction, excess energy at mid-range spatial frequencies and anisotropic distributions of contrast energy across orientations have been identified as key determinants of visual discomfort [2,13,14].

However, visual discomfort and esthetic preference have rarely been examined simultaneously, and directly comparing these responses to the same visual stimuli may provide important insights into the extent to which they reflect distinct affective experiences [2,6,15]. For example, Fernandez and Wilkins [2] measured participants’ liking of abstract paintings along with the degree of discomfort experienced while viewing them, finding a significant negative correlation between the two ratings. Similarly, Juricevic et al. [6] reported that ratings of artistic merit and visual discomfort showed opposite patterns as a function of variations in luminance and chromatic image statistics. Importantly, however, in both studies the associations between these measures were only moderate, suggesting that preference and discomfort do not reflect identical perceptual judgments.

Further evidence for this distinction comes from a study by Burtan et al. [15], who examined the effects of image complexity, preference, and discomfort on gait dynamics. Participants walked towards projected fractal images of three types (edge, grayscale, and thresholded) that varied in fractal dimension, while measures of walking speed, stride time, and step length were recorded. At the end of each trial, one group of participants rated how much they liked the image, whereas a second group rated the level of discomfort it evoked. Consistent with previous findings, participants walked fastest towards fractals of intermediate complexity whose statistical properties resembled those commonly found in natural scenes. Notably, velocity was predicted by an interaction between fractal dimension and rated visual discomfort, whereas preference ratings showed no such relationship. This dissociation further suggests that visual discomfort and preference capture distinct, albeit partially overlapping, aspects of the perceptual experience [15].

While these findings suggest that preference and discomfort are distinct but related perceptual experiences that vary in response to how closely stimuli align with natural scene statistics, most studies do not consider the variable and individualistic nature of these types of affective experiences. For example, Spehar et al. [10] and Güçlütürk et al. [16] have shown that, despite an overall preference for natural or synthetic images of intermediate complexity, there are pronounced individual differences in the preference for natural scene statistics. While some participants indeed prefer the images sharing the natural scene statistics (or intermediate complexity), the others show distinct preferences for images with either low or high complexity.

Additionally, individual differences in discomfort in response to visual stimuli have rarely been considered. Recent research examining visual discomfort and migraine has resulted in the development of a new targeted measurement tool for visual discomfort—The Cardiff Hypersensitivity Scale—Visual (CHYPS-V, [17]). In addition to a focus on the functional impact of visual discomfort and the identification of subtypes of visual sensitivity, CHYPS-V has been used to investigate the experience of visual discomfort across a range of clinical diagnoses and areas of neurodiversity, including migraine, dyslexia, eating disorders and fear disorders, as well as autism and ADHD [5].

## 2. The Present Study

The purpose of the present study is to explore the relationship between preference and discomfort ratings in response to synthetic images varying in fractal scaling-based complexity from the perspective of individual differences. As altered preference and increased discomfort in response to visual stimuli has been reported in both neurodiverse [18] and highly sensitive individuals [17], we aimed to evaluate the extent to which preference, discomfort and complexity ratings to these images would co-vary with self-reported autistic-and ADHD-like traits as well as sensory sensitivity. Self-reported autistic and ADHD traits were measured using the Comprehensive Autistic Trait Inventory (CATI, [19]) and the Adult ADHD Self-Report Scale (ASRS, [20]). Sensory sensitivity was measured using the Sensory Perception Quotient (SPQ-S), a well-validated tool for measuring sensory processing sensitivity [21], and the Cardiff Hypersensitivity Scale—Visual (CHYPS-V) [17].

**Figure 1:**
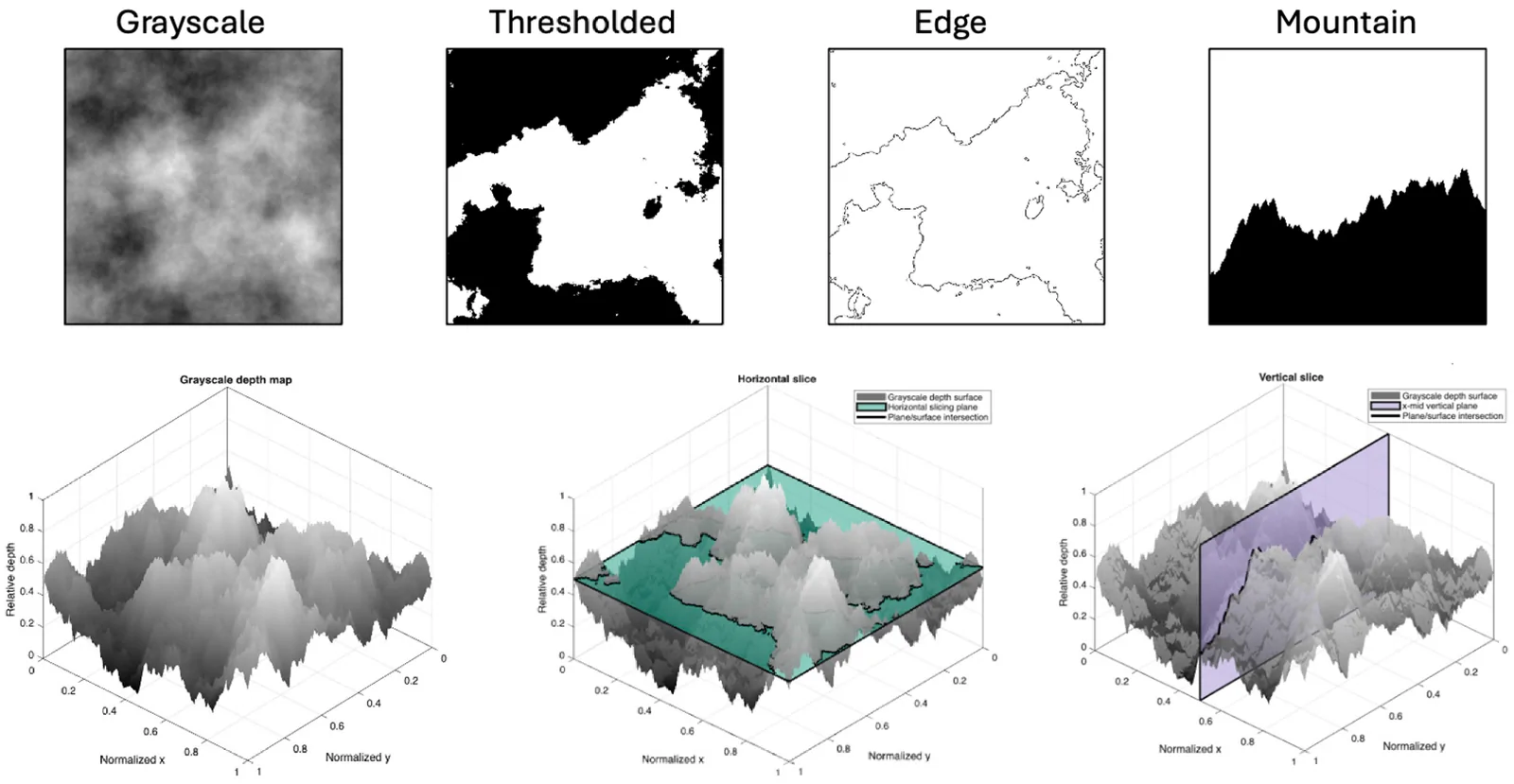
Types of image variants used in the present study: (**Top**) From left to right, examples of grayscale, thresholded, edges, and mountain images. (**Bottom**) An illustration of how thresholded, edge and mountain images were derived from the seed grayscale images.

To measure perceptual and affective ratings, this study used unbiased static statistical fractal stimuli varying in visual complexity. We used four different types of fractal patterns, allowing us to determine whether the effect of fractal scaling on affective ratings of stimuli transcends image type. The derivation of and the relationship between the four types of patterns used in this study are illustrated in Figure 1. The grayscale image in Figure 1(top panel) represents a seed image, while the thresholded image is the binary variant of the grayscale pattern and the edges version is generated by extracting edges from the thresholded image. The bottom panel in Figure 1 depicts how the thresholded, edge and mountain images are related to and represent horizontal and vertical slices respectively of the depth map derived from the seed grayscale image.

**Figure 2:**
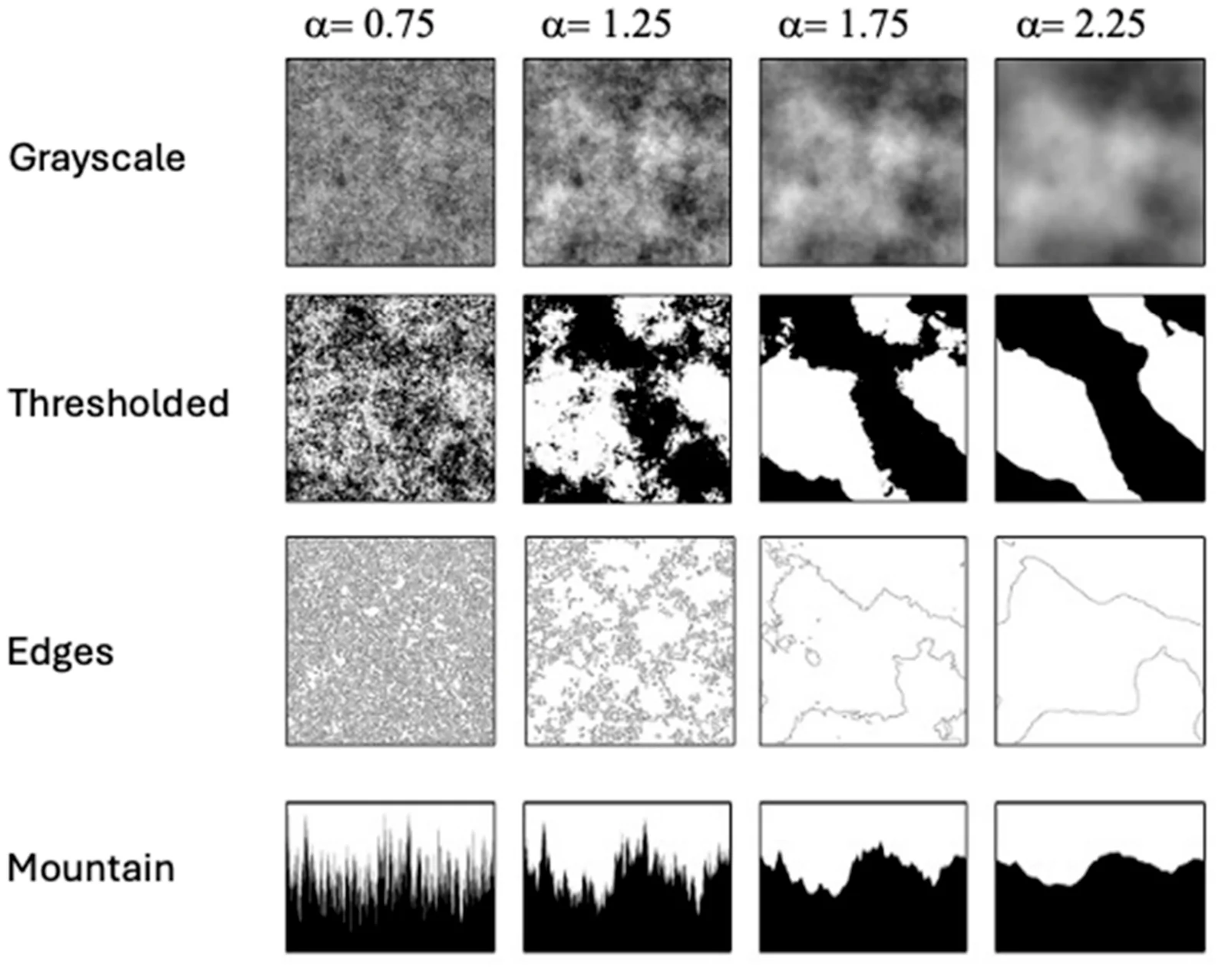
Examples of variations in fractal scaling characteristics in the grayscale, thresholded, edges and mountain image variants. Images vary in complexity from left (high complexity) to right (low complexity).

Variations in fractal scaling across these image variants are defined by changes in the 1/f amplitude spectrum slope (α) of the seed grayscale image and are illustrated in Figure 2. Importantly, although the same input α values were used to generate both the grayscale and modified images, the thresholding, edge extraction, and mountain slice procedures alter the images’ photometric properties and consequently their resulting 1/f amplitude spectra. As a result, thresholded, edge, and mountain images generally exhibit shallower 1/f slopes than their corresponding grayscale images, particularly at steeper input α values. Despite these changes in spectral slope, the relationship between coarse and fine spatial detail is preserved across image transformations, and the geometric (fractal scaling) properties of the modified images remain nearly identical to those of their grayscale counterparts. In both grayscale and modified images, fractal scaling reflects the relative balance of coarse and fine spatial structure; however, this balance is quantified using different metrics. In grayscale images, it is determined by the relative amplitude or energy at low and high spatial frequencies, whereas in thresholded, edge, and mountain images it is reflected in the density of coarse versus fine spatial structures [22,23,24].

The main goal of this study is to investigate how preference and discomfort ratings are shaped by the statistical complexity of visual stimuli at both population and individual levels. To confirm that physical variations in fractal scaling characteristics are mirrored at the perceptual levels, we will measure the perceived complexity of these images as well. Further, we aim to examine the extent to which measures of sensory sensitivity and neurodivergent traits co-vary with perceptual and affective ratings.

## 3. Method

### 3.1. Design

This study employed a 4 (image type: grayscale, thresholded, edges, mountain) × 4 (fractal scaling variations: 0.75, 1.25, 1.75, 2.25) repeated measures design. The three dependent variables are: ratings of preference, discomfort and complexity. Participant variables were also included as a covariate—the level of self-reported autistic-like traits, ADHD traits and sensory processing sensitivity.

### 3.2. Participants

This study recruited a total of 102 participants (73 female, 28 male, 1 other) aged 18–38 (M = 19.4, SD = 2.53). Participants were first-year psychology undergraduates, recruited through the SONA platform at the University of New South Wales in exchange for a course credit. All participants indicated normal or corrected-to-normal vision. No further ocular health information was collected. Participants were required to give informed consent prior to beginning the experiment. After study completion, participant ratings for visual stimuli were screened for insufficient effort responding using the Individual Response Variability Index (IRV), whereby participants were flagged if their response variability was more than 2 SD below the sample mean, in line with research from [25]. This resulted in the removal of 13 participants who gave the same rating on all trials within at least one of the rating blocks, leaving 89 total participants included in this study (65 female, 23 male, 1 other) aged 18–38 (M = 19.0, SD = 2.56). Ethics approval for this research was granted by the Research Ethics and Compliance Support (RECS) team at the University of New South Wales, Sydney.

### 3.3. Materials and Equipment

#### 3.3.1. Visual Images

The grayscale images were constructed by first creating a 512 × 512 pixels random noise pattern, with each pixel value (0–255) selected from a Gaussian distribution. A Fourier transform was then performed to obtain the amplitude frequency spectrum, which was adjusted to create a range of spectral slopes ranging from 0.75 to 2.25 in increments of 0.5. The four resulting experimental images had amplitude spectrum slope α values of 0.75, 1.25, 1.75, and 2.25 (see Figure 2). These steps were repeated to create twelve different seed images, used to create thresholded, edges and mountain variants, as illustrated in Figure 1 and Figure 2. In total, 192 different experimental images were created.

#### 3.3.2. Individual Differences Questionnaires

Comprehensive Autistic Trait Inventory (CATI). CATI is a 42-item neurodiversity affirming questionnaire measuring the presence of self-reported autistic-like traits. CATI has high internal consistency (Cronbach’s alpha = 0.969 for total scale, 0.855–0.941 for subscales), and strong convergent validity with existing measures of autistic traits [19]. Respondents answer questions on a 5-point Likert scale from definitely disagree (1) to definitely agree (5), and receive a total score ranging from 42 to 210, with higher scores indicating more self-reported autistic-like traits.

Adult ADHD Self Report Scale (ASRS). ASRS is an 18-item self-report screening scale for ADHD with questions mapping onto the key criterion of DSM-IV diagnosis of ADHD. ASRS has shown high internal consistency (Cronbach’s alpha = 0.88) and concurrent validity (r = 0.84), as well as moderate predictive power (0.52) [20]. When completing ASRS, participants are asked questions about their behavior in the last six months and asked to respond on a 5-point Likert scale ranging from never (0) to very often (4). Total scores range from 0 to 72, with higher scores indicating the presence of more ADHD traits.

Cardiff Hypersensitivity Scale—Visual (CHYPS-V). CHYPS-V [17] is a new scale measuring visual hypersensitivity across the four domains of pattern (“stripey and repeating patterns and pictures seem to shimmer, flicker or move when I look at them”), strobing (“I close my eyes or look away if there are strobing or flashing lights on a TV program or a film at the cinema”), brightness (“I turn off or dim bright ceiling lights because they make my eyes or head feel uncomfortable”) and intense visual environments (“I find everyday environments with a lot of movement uncomfortable for my eyes and head”). While it is yet to be widely used in research, it has shown good reliability (Cronbach’s alpha = 0.94), as well as convergent validity with existing measures of visual sensitivity (r = 0.69–0.86) [17]. Participants responded to 20 questions on a 4-point Likert scale ranging from almost never (0) to almost always (3) and received a sum score as well as scores across the four domains, with higher scores indicating higher visual sensitivity.

Sensory Perception Quotient—Short (SPQ-S). SPQ-S was developed to address the need for a questionnaire that investigates only basic sensory sensitivity and not affective response [21]. The result is a 35-item questionnaire that measures sensory sensitivity across vision, hearing, touch, smell and taste. Items are scored on a 4-point Likert scale from strongly agree (0) to strongly disagree (3), such that lower total scores indicate more sensory difficulties. SPQ-S has been shown to have excellent internal consistency and good concurrent validity [21] and has been used extensively in research to measure hypersensitivity [26].

### 3.4. Procedure

**Figure 3:**
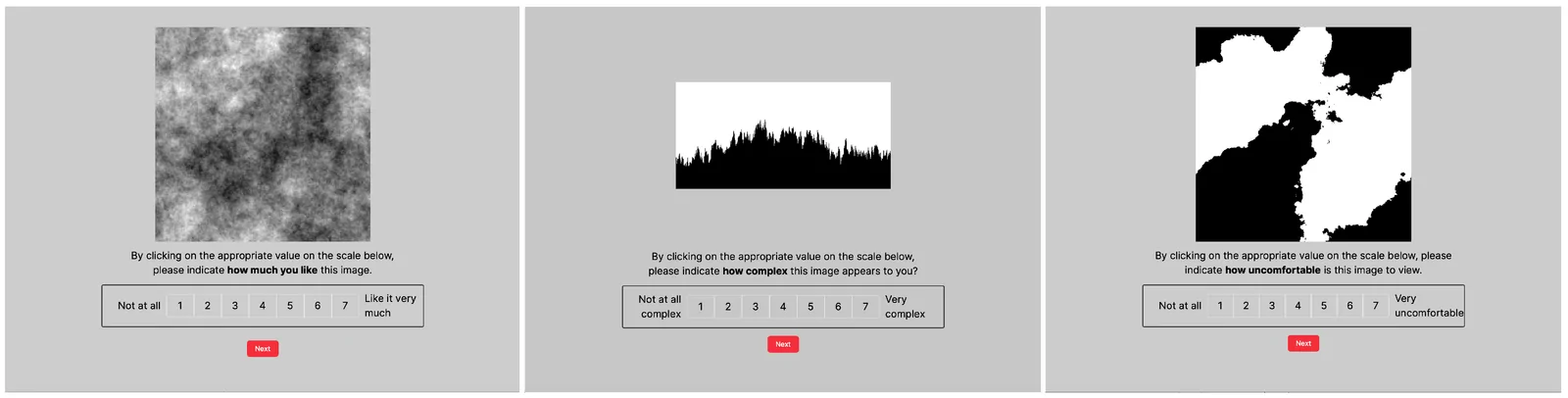
Examples of preference (**left panel**), complexity (**middle panel**) and discomfort (**right panel**) trials.

Gorilla Experiment Builder software (Cauldron Science, Cambridge, UK) was used to present stimuli and collect image evaluation and questionnaire responses. Participants completed the experiment online on their own desktop or laptop computer devices. The use of mobile phones and tablet devices was not permitted, with no further instructions for viewing distance, screen settings or room lighting. Most of the participants completed the experiment on Apple (69%)- or Windows (30%)-operated devices. To accommodate differences in aspect ratios across different devices, all display elements were positioned within the largest 4:3 landscape box that can be fitted on the screen. Further, the 4:3 virtual area is divided into a 24 × 18 virtual grid used as a coordinate system to maintain the consistent aspect ratio and layout of stimuli. The dimension of grayscale, edges and thresholded stimuli was 10 × 10 in the virtual grid coordinates with the mountain stimuli subtending 10 × 5 units. The relative size and layout of experimental stimuli across the three rating tasks are shown in Figure 3.

Participants first completed image evaluations in which they rated perceived preference, discomfort, and complexity in separate blocks, with block order randomized across participants. Each block consisted of 48 trials, comprising 12 ratings for each of the four image types. Within each image type, participants completed three ratings at each of the four fractal scaling levels. The three occurrences of each image type × fractal scaling combination used differently seeded stimuli, ensuring that no individual stimulus image was presented more than once.

In the preference block, participants were asked to indicate how much they liked each image on a 7-point Likert scale from 1 (not at all) to 7 (like very much) (Figure 3, left panel). In the complexity block, participants were asked to rate how complex each image appeared to them on a 7-point Likert scale from 1 (not at all complex) to 7 (very complex) (Figure 3, middle panel). In the discomfort block, participants were asked to indicate how uncomfortable each image was to view on a 7-point Likert scale from 1 (not at all) to 7 (very uncomfortable) (Figure 3, right panel). No image was repeated in any of the blocks so that participants viewed a total of 144 images in the image evaluation section. The image evaluation section took approximately 15–20 min to complete.

Participants then completed the set of four individual difference measures. The order of these was the same for all participants: ASRS, CATI, CHYPS-V and SPQ-S. Each questionnaire was preceded by a short preamble explaining the questionnaire’s length and followed by a postamble thanking participants for completing the questionnaire and reminding them how many questionnaires remained. At the completion of the questionnaire block, participants were thanked for completing the experiment and shown a debriefing form providing them information about this study and optional additional reading should they be interested in the research area. The questionnaire section took approximately 25–30 min to complete.

### 3.5. Data Analysis

To examine the effects of image statistics on perceived complexity, discomfort, and preference, separate linear mixed-effects models were fitted for trial-level complexity, discomfort, and preference ratings. Each model included 4272 observations from 89 participants and was estimated using linear mixed-effects modeling. Initial models included the spectral slope fractal scaling parameter (four levels) and image type (four levels) as categorical fixed factors, together with their interaction. Random effects included participant- and stimulus-specific intercepts, accounting for variability in overall ratings across both participants and individual stimulus images. (Satterthwaite approximations were used for denominator degrees of freedom; consequently, denominator degrees of freedom differed across outcome models according to their estimated random effects variance structure. In particular, residual stimulus-level variance was estimated as nonzero for complexity ratings but was effectively zero for discomfort and preference ratings.)

We additionally examined whether different types of ratings changed systematically over the course of the task or as a function of repeated exposure to the experimental conditions. Trial number was included as a continuous predictor, and presentation instance (i.e., the first, second, or third) of differently seeded exemplars of each image type × fractal scaling combination was included as a categorical predictor. To account for individual differences in temporal response patterns, trial number slopes were allowed to vary across participants.

Following the primary analyses, we conducted separate covariate analyses for each rating outcome. The four participant-level covariates (standardized SPQ-S, CATI, CHYPS-V, and ASRS scores) were entered simultaneously as continuous predictors, alongside the experimental factors of image type and fractal scaling and their interaction. Participant and stimulus were retained as crossed random intercepts. This approach allowed the unique association of each covariate with ratings to be estimated while adjusting for the other covariates and the experimental effects. The reported partial eta squared values were estimated from the omnibus F statistics using $\eta_{p}^{2}=(F\times df_{1})/(F\times df_{1}+df_{2})$. The Holm method was used to adjust for pairwise comparisons and moderation follow-ups.

## 4. Results

### 4.1. Questionnaire Results

Mean scores and standard deviations for each of the four trait measures (CATI, ASRS, CHYPS-V and SPQ-S) are reported in Table 1.

Pearson correlations among the individual difference measures are presented in Table 2. CATI scores correlated positively with ASRS scores, r = 0.55, 95% CI [0.38, 0.68], and CHYPS-V scores, r = 0.53, 95% CI [0.36, 0.66], and negatively with SPQ-S scores, r = −0.26, 95% CI [−0.45, −0.06]. ASRS scores correlated positively with CHYPS-V scores, r = 0.41, 95% CI [0.22, 0.57], and negatively with SPQ-S scores, r = −0.31, 95% CI [−0.49, −0.11]. CHYPS-V and SPQ-S scores were moderately negatively correlated, r = −0.50, 95% CI [−0.64, −0.32]. All correlations were statistically significant after Holm adjustment.

### 4.2. Complexity Ratings

#### 4.2.1. Initial Analyses

**Figure 4:**
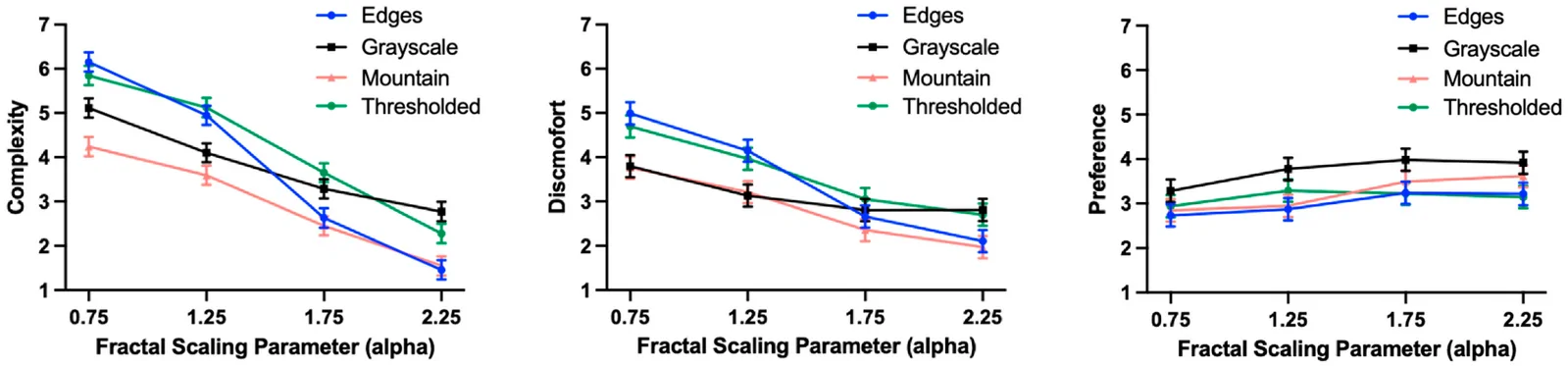
Estimated marginal means of rated complexity (**left panel**), discomfort (**middle panel**) and preference (**right panel**) as a function of fractal scaling parameter (alpha) and image type. Error bars represent 95% confidence intervals.

Across all image types, higher spectral slopes were associated with lower perceived complexity, with the strength of this relationship varying by image category (Figure 4, left panel). A linear mixed-effects model with fractal scaling (0.75, 1.25, 1.75, and 2.25) and image type (edge, grayscale, mountain, and thresholded) as categorical fixed factors together with their interaction and participant- and stimulus-specific intercepts was fitted to the complexity ratings. The full model explained 54.5% of the variance through fixed effects (R_m_^2^ = 0.545) and 66.7% when both fixed and random effects were considered (R_c_^2^ = 0.667).

The omnibus tests revealed significant main effects of fractal scaling, *F*(3, 32.00) = 1272.23, *p* < 0.001, ηp^2^ = 0.99, indicating that images with steeper spectral slope parameters were rated as less complex. The main effect of image type, *F*(3, 32) = 165.1, *p* < 0.001, ηp^2^ = 0.94, was also significant with thresholded (M = 4.23, 95% CI [4.07, 4.39]) and mountain (M = 2.96, 95% CI [2.80, 3.12]) images receiving the highest and lowest complexity ratings respectively.

These main effects were qualified by a significant fractal scaling × image type interaction, *F*(9, 32) = 39.87, *p* < 0.001, ηp^2^ = 0.92, indicating that the effect of fractal scaling on perceived complexity differed across image types. The simple effects analyses confirmed a significant effect of fractal scaling for all image types: edge, *F*(3, 32) = 667.42, *p* < 0.001; grayscale, *F*(3, 32) = 152.28, *p* < 0.001; mountain, *F*(3, 32) = 205.75, *p* < 0.001; and thresholded, *F*(3, 32) = 366.40, *p* < 0.001. There was also a significant effect of image type at all fractal scaling levels: 0.75, *F*(3, 32) = 106.85, *p* < 0.001; 1.25, *F*(3, 32) = 75.73, *p* < 0.001; 1.75, *F*(3, 32) = 45.769, *p* < 0.001; and 2.25, *F*(3, 32) = 56.33, *p* < 0.001. The decrease in rated complexity from α = 0.75 to α = 2.25 was greatest for edge (Δ = −4.69) and thresholded images (Δ = −3.57) and more gradual for grayscale (Δ = −2.34) and mountain images (Δ = −2.67). The overall decrease in rated complexity was significantly different between all pairs of image types. The detailed model output, estimated marginal means, and pairwise comparisons are provided in Supplementary Materials (Section S1).

The follow-up analysis, with trial number as a continuous predictor, revealed no evidence of systematic trial order changes over the course of the task, *F*(1, 211.23) = 1.04, *p* = 0.309 or across successive presentations of differently seeded exemplars representing the same fractal scaling × image type experimental condition, *F*(2, 4097.16) = 2.34, *p* = 0.096. Complete model results, including estimated marginal means and pairwise comparisons, are presented in Supplementary Materials (Section S2).

#### 4.2.2. Covariate Analyses

Following the primary analysis of complexity ratings, the four participant-level covariates (SPQ-S, CATI, CHYPS-V, and ASRS scores) were entered simultaneously as continuous standardized predictors, alongside the experimental factors of image type and fractal scaling and their interaction (see Supplementary (Section S3)). Participant and stimulus were retained as crossed random intercepts. The full model with individual differences as covariates explained 55.5% of the variance through fixed effects (R_m_^2^ = 0.555) and 67.3% when both fixed and random effects were considered (R_c_^2^ = 0.673). The effects of fractal scaling and image type and their interaction remained unchanged after inclusion of the covariates. In addition, none of the individual difference measures showed significant main effects (all *ps* > 0.05), indicating that these variables were not associated with overall complexity ratings.

**Figure 5:**
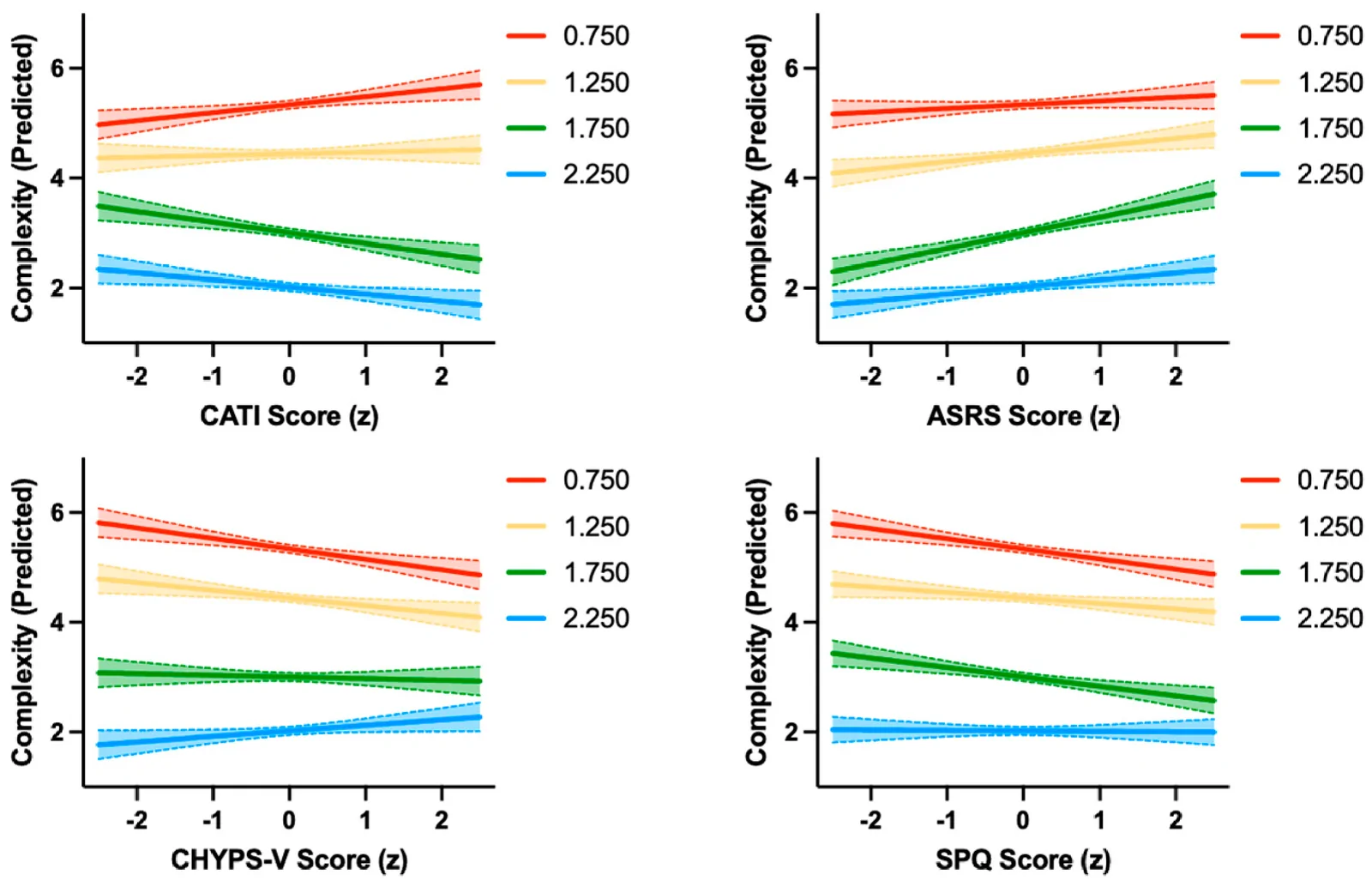
Predicted ratings for complexity as a function of standardized covariate scores, with separate lines for the four fractal scaling levels and shaded 95% confidence intervals: CATI (**top left panel**), ASRS (**top right panel**), CHYPS-V (**bottom left panel**) and SPQ-S (**bottom right panel**). Note: Values were derived from the full linear mixed-effects model, including fractal scaling parameter, image type, CATI, CHYPS-V, ASRS, and SPQ-S, together with covariate interactions with fractal scaling. Remaining covariates were held constant at their sample means. Shaded confidence bands represent 95% confidence intervals. Predictions are averaged across image type.

However, the association between each of the four individual difference measures and complexity ratings varied at different levels of fractal scaling, as illustrated in Figure 5.

As shown in Figure 5, significant interactions were observed for fractal scaling and CATI, *F*(3, 4124) = 12.23, *p* < 0.001 ηp^2^ = 0.009; ASRS, *F*(3, 4124) = 4.84, *p* = 0.002, ηp^2^ = 0.004; CHYPS-V, *F*(3, 4124) = 8.60, *p* < 0.001, ηp^2^ = 0.006; and SPQ-S, *F*(3, 4124) = 4.27, *p* = 0.005, ηp^2^ = 0.003. As evident in Table 3, these interactions primarily reflect differences in the strength and, in some cases, direction of the covariate associations across different levels of fractal scaling. Although all four measures moderated the effect of fractal scaling on complexity, the direction and form of this moderation differed across the individual difference measures.

The CATI × fractal scaling relationship indicates that the association between CATI and rated complexity became increasingly negative as fractal scaling increased, with the stronger negative associations at 1.75 and 2.25 fractal scaling levels. The ASRS × scaling interaction was somewhat less monotonic, with the principal change occurring at scaling level 1.75, where the association between ASRS and complexity was more positive than at the lower scaling levels. For CHYPS-V, the association similarly changed across scaling levels but in a positive direction, shifting from relatively weak or negative associations at lower fractal scaling levels toward a more positive association at higher levels, particularly at 2.25. The SPQ-S × fractal scaling interaction reflected a shift toward a relatively more positive association between SPQ-S scores and complexity at the highest scaling level, particularly at 2.25. Full parameter estimates for all covariate effects in this model are reported in Supplementary Materials (Section S3).

### 4.3. Discomfort Ratings

#### 4.3.1. Initial Analyses

As shown in Figure 4 (middle panel), discomfort ratings also decreased with increasing fractal scaling values across all image types, although the magnitude of this decrease varied by image type. The ratings were analyzed using the same linear mixed-effects model with alpha slope (0.75, 1.25, 1.75, 2.25) and image type (edge, grayscale, mountain, thresholded) as categorical fixed effects together with their interaction and participant- and stimulus-specific random intercepts. The full model explained 21% of the variance through fixed effects (R_m_^2^ = 0.209) and 43% when both fixed and random effects were considered (R_c_^2^ = 0.429).

The main effect of fractal scaling was significant, *F*(3, 4168) = 399.98, *p* < 0.001, ηp^2^ = 0.22, indicating that discomfort ratings decreased systematically as fractal scaling values increased. A significant main effect of image type was also observed, *F*(3, 4168) = 62.76, *p* < 0.001, ηp^2^ = 0.05, with thresholded (M = 3.60, 95% CI [3.39, 3.80]) and edge (M = 3.49, 95% CI [3.28, 3.69]) images rated as more uncomfortable overall than grayscale (M = 3.13, 95% CI [2.93, 3.34]) and mountain (M = 2.83, 95% CI [2.62, 3.03]) images. The differences in overall rated discomfort between all image type pairings were statistically significant, except between the thresholded and edge images.

The fractal scaling × image type interaction was significant, *F*(9, 4168) = 18.58, *p* < 0.001, ηp^2^ = 0.04, indicating that the effect of spectral slope on discomfort varied across image types. Follow-up simple effects analyses confirmed significant effects of fractal scaling for all image types: edge, *F*(3, 4168) = 232.88, *p* < 0.001; grayscale, *F*(3, 4168) = 28.80, *p* < 0.001; mountain, *F*(3, 4168) = 87.06, *p* < 0.001, and thresholded images, *F*(3, 4168) = 106.972, *p* < 0.001. There was also a significant effect of image type at all fractal scaling levels: 0.75, *F*(3, 4168) = 52.23, *p* < 0.001; 1.25, *F*(3, 4168) = 35.49, *p* < 0.001; 1.75, *F*(3, 4168) = 10.29, *p* < 0.001; and 2.25, *F*(3, 4165) = 22.48, *p* < 0.001. Although overall discomfort decreased with increasing fractal scaling for all image types, this reduction was substantially larger for edge images (Δ = −2.92) than for grayscale images (Δ = −0.99). Full model output and follow-up analyses are provided in Supplementary Materials (Section S4).

Unlike with the complexity ratings, the follow-up analysis revealed a significant effect of trial number, *F*(1, 186.48) = 5.237, *p* = 0.022, such that there was a small increase in discomfort ratings over the course of rating block (β = 0.007, 95% CI [0.001, 0.013]). The effect of presentation instance was not significant *F*(2, 4093.02) = 2.07, *p* = 0.126. Complete model results, including estimated marginal means and pairwise comparisons, are presented in Supplementary Materials (Section S5).

#### 4.3.2. Covariate Analyses

As with the complexity ratings, following the primary analysis, the four participant-level covariates (SPQ-S, CATI, CHYPS-V, and ASRS scores) were entered simultaneously as continuous standardized predictors of rated discomfort, alongside the experimental factors of image type and fractal scaling and their interaction. Participant and stimulus were retained as crossed random intercepts. The full model with individual differences as covariates explained 23.0% of the variance through fixed effects (R_m_^2^ = 0.230) and 44% when both fixed and random effects were considered (R_c_^2^ = 0.440). The effects of fractal scaling and image type and their interaction remained unchanged after inclusion of the covariates. Although no significant main effects for rated discomfort were observed for CATI, ASRS, or SPQ-S, a small positive effect of CHYPS-V was detected, *F*(1, 84) = 4.35, *p* = 0.040, ηp^2^ = 0.004. The participants with higher reported CHYPS-V scores gave higher discomfort ratings overall (β = 0.258, 95% CI [0.015, 0.501]).

**Figure 6:**
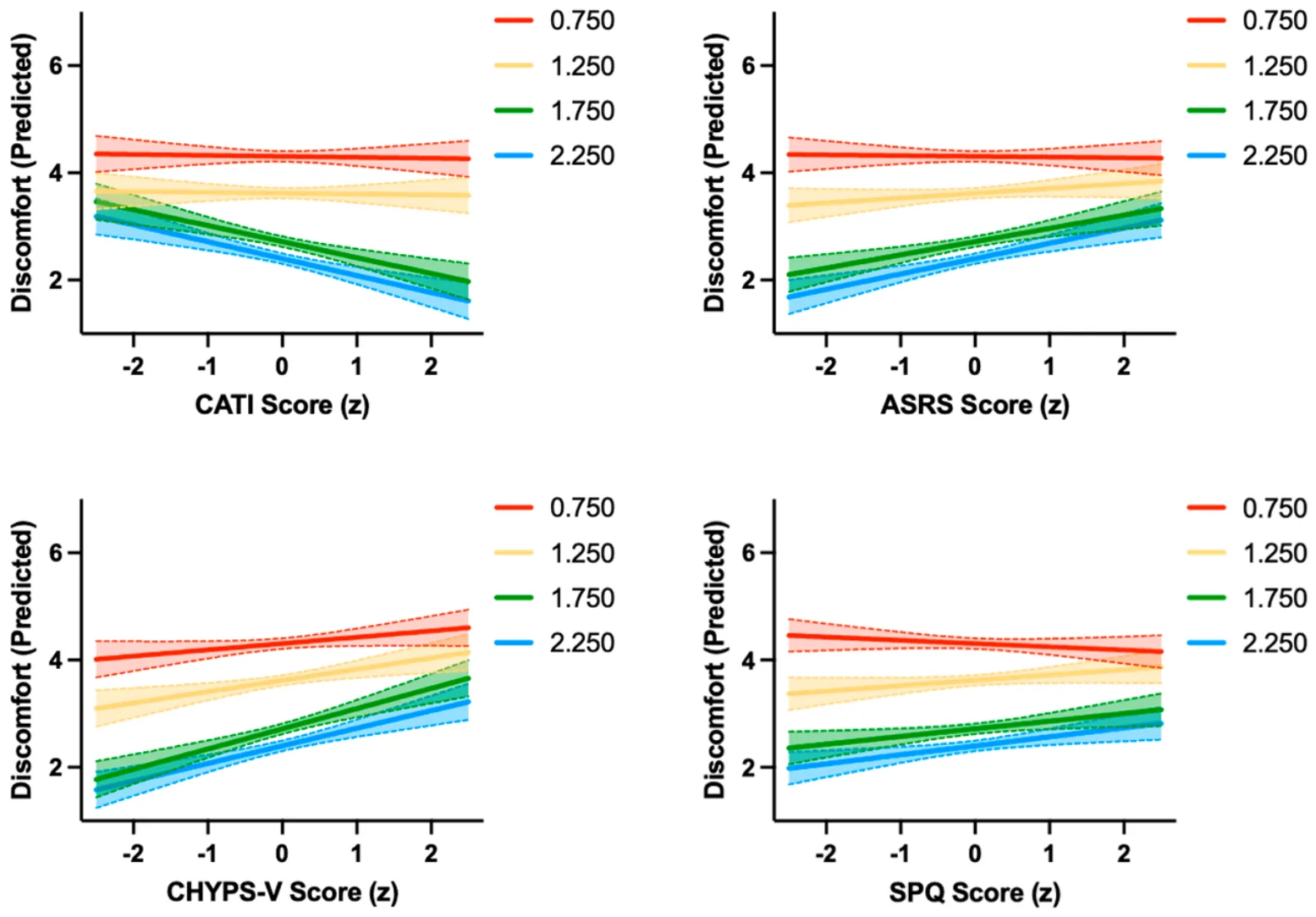
Estimated marginal means for discomfort ratings as a function of standardized scores for CATI (**top left panel**), ASRS (**top right panel**), CHYPS-V (**bottom left panel**) and SPQ-S (**bottom right panel**). Note: Values were derived from the full linear mixed-effects model including spectral slope, image type, CATI, CHYPS-V, ASRS, and SPQ-S, together with their interactions with spectral slope. Remaining covariates were held constant at their sample means. Shaded confidence bands represent 95% confidence intervals.

In addition, significant interactions were observed between fractal scaling and all four individual differences covariates: CATI, *F*(3, 4156) = 8.84, *p* < 0.001, ηp^2^ = 0.006; ASRS, *F*(3, 4156) = 6.90, *p* < 0.001, ηp^2^ = 0.005; CHYPS-V, *F*(3, 4156) = 4.26, *p* = 0.005, ηp^2^ = 0.003; and SPQ-S, *F*(3, 4156) = 4.17, *p* = 0.006, ηp^2^ = 0.003. These moderating effects, which were relatively small in magnitude, differed across the covariates, as illustrated in Figure 6.

As evident from the simple slopes detailed in Table 4, the effect of CATI (Figure 6, top left panel) was essentially absent at lower fractal scaling levels but the relationship with the rated discomfort became progressively more negative for steeper slopes: participants with higher CATI scores rated images with higher fractal scaling characteristics as less uncomfortable compared with participants with lower CATI scores. The pattern for the other three individual differences measures was apparently reversed. The association between ASRS and discomfort (Figure 6, top right panel) became more positive at higher fractal scaling levels: participants with higher ASRS scores reported greater discomfort for images with higher fractal scaling characteristics compared with participants with lower scores. CHYPS-V (Figure 6, bottom left panel) shows a broadly similar pattern with higher CHYPS-V score becoming increasingly associated with greater discomfort as fractal scaling increased, with this positive association particularly evident at 1.75 and 2.25 fractal scaling levels. Finally, higher SPQ-S scores (Figure 6, bottom right panel) were also associated with an increasingly positive relationship with discomfort at higher fractal scaling levels. Full parameter estimates for all covariate effects in this model are reported in Supplementary Materials (Section S6).

### 4.4. Preference Ratings

#### 4.4.1. Initial Analyses

As shown in Figure 4 (right panel), preference ratings generally increased with increasing fractal scaling values across image types, but the overall linear trend was not as pronounced as it was with the complexity and discomfort ratings. The same linear mixed-effects model was fitted to preference ratings, with fractal scaling, image type and their interaction included as fixed effects and participant- and stimulus-specific random intercepts. The full model explained 4.6% of the variance through fixed effects (R_m_^2^ = 0.046) and 29.4% when both fixed and random effects were considered (R_c_^2^ = 0.294).

The omnibus tests revealed a significant main effect of fractal scaling, *F*(3, 4168) = 32.43, *p* < 0.001, ηp^2^ = 0.023, such that preference ratings increased from *M* = 2.95 (95% CI [2.75, 3.15]) at α = 0.75 to *M* = 3.49 (95% CI [3.29, 3.69]) at α = 1.75 and remained elevated at α = 2.25 (*M* = 3.47, 95% CI [3.27, 3.67]). Pairwise comparisons showed that all slope conditions differed significantly from one another (all *ps* < 0.001), except the comparison between α = 1.75 and α = 2.25 (*p* = 0.789). The main effect of image type was also significant, *F*(3, 4168) = 50.53, *p* < 0.001, ηp^2^ = 0.035, with grayscale images receiving the highest preference ratings overall (*M* = 3.74, 95% CI [3.54, 3.94]), followed by mountain (*M* = 3.23, 95% CI [3.03, 3.43]), thresholded (*M* = 3.14, 95% CI [2.94, 3.34]), and edge images (*M* = 3.02, 95% CI [2.83, 3.22]).

The effect of image type on preference varied significantly as a function of fractal scaling, *F*(9, 4168) = 3.29, *p* < 0.001, ηp^2^ = 0.007. Simple effects analyses showed a significant effect of fractal scaling for all image types: edge, *F*(3, 4168) = 8.05, *p* < 0.001; grayscale, *F*(3, 4168) = 12.35, *p* < 0.001; mountain, *F*(3, 4168) = 18.77, *p* < 0.001; and thresholded, *F*(3, 4168) = 3.15, *p* = 0.024. There was also a significant effect of image type at all fractal scaling levels: 0.75, *F*(3, 4168) = 7.31, *p* < 0.001; 1.25, *F*(3, 4168) = 20.49, *p* < 0.001; 1.75, *F*(3, 4168) = 15.71, *p* < 0.001; and 2.25, *F*(3, 4168) = 16.90, *p* < 0.001. Grayscale images were consistently preferred over edge images across all four scaling levels, with the largest difference at scaling 1.25. The relative preference for the other image types varied more modestly with scaling: thresholded images were preferred over edge images at scaling 1.25, whereas mountain images were preferred over edge images at the higher scaling levels (1.75 and 2.25). Full model output, estimated marginal means, and pairwise comparisons are reported in Supplementary Materials (Section S7).

The follow-up model revealed no evidence of systematic trial number changes in preference over the course of the task, F(1, 233.91) = 0.138, *p* = 0.710, or across successive presentation instances, F(2, 4072.81) = 1.53, *p* = 0.217. Complete model results, including estimated marginal means and pairwise comparisons, are presented in Supplementary Materials (Section S8).

#### 4.4.2. Covariate Analyses

To examine whether individual differences were associated with preference responses to fractal scaling, SPQ-S, CATI, CHYPS-V, and ASRS scores were entered simultaneously as continuous predictors, together with their interactions with fractal scaling. As before, image type, fractal scaling, and their interaction were retained as experimental fixed effects, while participant and stimulus were included as crossed random intercepts. The full model with individual differences as covariates explained 6.1% of the variance through fixed effects (R_m_^2^ = 0.061) and 31.4% when both fixed and random effects were considered (R_c_^2^ = 0.314). The effects of fractal scaling and image type and their interaction remained unchanged after inclusion of the covariates. In addition, none of the individual difference measures showed significant main effects (all *ps* > 0.45), indicating that these variables were not associated with overall preference ratings.

**Figure 7:**
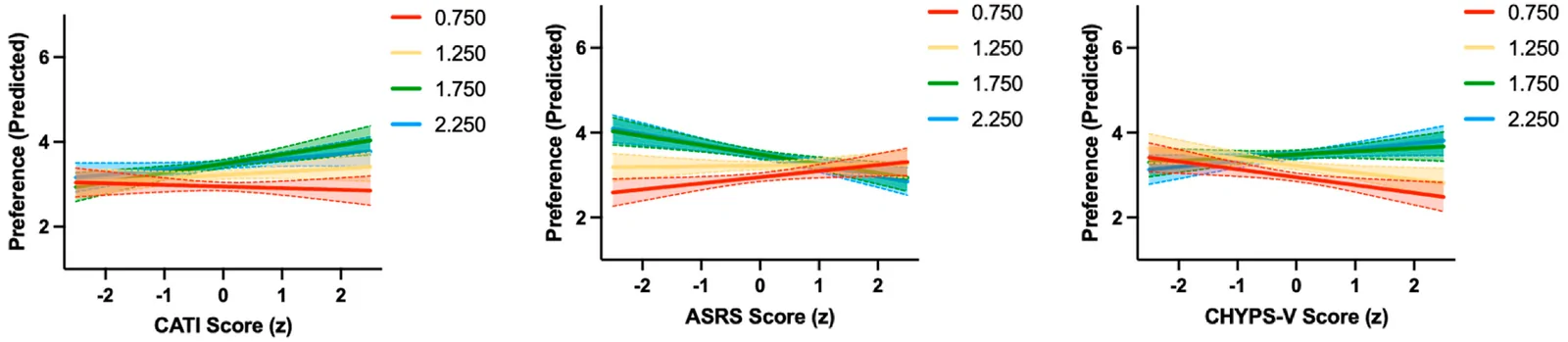
Estimated marginal means for preference ratings as a function of standardized scores for CATI (**left panel**), ASRS (**middle panel**) and CHYPS-V (**right panel**). *Note:* Values were derived from the full linear mixed-effects model including spectral slope, image type, CATI, CHYPS-V, ASRS, and SPQ-S, together with their interactions with spectral slope. Remaining covariates were held constant at their sample means. Shaded confidence bands represent 95% confidence intervals.

As illustrated in Figure 7, significant interactions for preference ratings were observed between fractal scaling and CATI, *F*(3, 4156) = 3.47, *p* = 0.016, ηp^2^ = 0.003; ASRS, *F*(3, 4156) = 12.19, *p* < 0.001, ηp^2^ = 0.009; and CHYPS-V, *F*(3, 4156) = 8.07, *p* < 0.001, ηp^2^ = 0.006. The interaction between fractal scaling and SPQ-S scores was not significant (*p* = 0.192). Full model output and parameter estimates are provided in Supplementary Materials (Section S9).

Follow-up analyses indicated that these interactions again reflected changes in the covariate slopes across scaling conditions (Table 5). The CHYPS-V association with preference ratings shows that participants with higher scores exhibit increasing/decreasing preference for lower/higher levels of fractal scaling respectively. The CATI association varied more modestly across scaling levels but exhibited a similar general trend to that observed with the CHYPS-V. In contrast, the ASRS association became increasingly negative at higher levels of fractal scaling. Thus, as with complexity and discomfort, the significant interactions indicate that individual difference associations with preference depended on the fractal scaling characteristics of the stimuli.

### 4.5. Comparisons Between Complexity, Discomfort, and Preference Ratings

**Figure 8:**
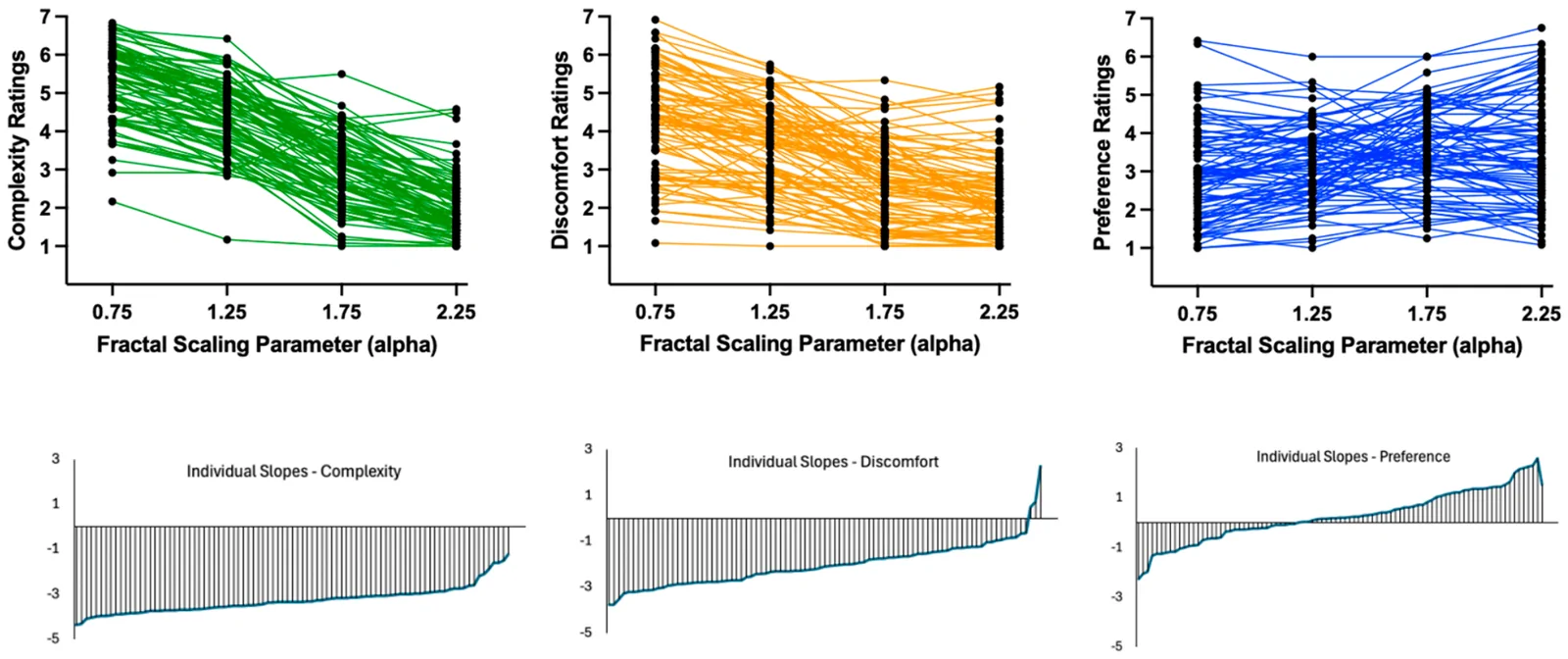
Individual differences in responses to fractal scaling for complexity, discomfort, and preference ratings. Upper panels show individual participant response profiles across fractal scaling, averaged across image types, for complexity (**left**), discomfort (**center**), and preference (**right**). Lower panels show model-estimated participant-specific fractal scaling slopes, calculated as the population-level fixed scaling slope plus each participant’s random slope estimate and ordered from the most negative to the most positive within each rating outcome.

Figure 8 (top row) illustrates individual participants’ ratings of complexity, discomfort and preference (averages across the four image types) as a function of variations in spectral slope. The panels illustrate the heterogeneity in individual responses for all three types of ratings, though the nature of individual variations differs considerably for different ratings. To characterize individual differences in sensitivity to fractal scaling, separate linear mixed-effects models were fitted for complexity, discomfort, and preference ratings, with fractal scaling entered as a continuous predictor and image type as a categorical factor. The models included the main effects of fractal scaling and image type and their interaction, together with participant-specific random intercepts and random slopes for fractal scaling. Participant-specific total scaling slopes were calculated by combining the population-level fixed scaling effect with each participant’s random slope estimate and are shown in Figure 8, bottom row. Complexity slopes were predominantly negative, indicating that perceived complexity generally decreased with increasing fractal scaling, although the magnitude of this relationship varied across participants. Discomfort showed a similar pattern, with predominantly negative slopes and a small number of participants showing weakly positive relationships (3 out of 89 participants). Preference showed a qualitatively different pattern and greater heterogeneity in the direction of the relationship itself, with individual slopes spanning both negative (36 out of 89 participants) and positive values (53 out of 89 participants). Similar interindividual variations in preference ratings have been reported before [10,16].

Associations among the three rating measures were examined in the following way. We first calculated between-person correlations using each participant’s mean rating across the 16 image type × spectral slope conditions. Second, we calculated within-person correlations after subtracting each participant’s overall mean from their condition-level ratings to remove differences in participants’ general use of the rating scales [27,28]. Finally, condition-adjusted correlations were calculated after additionally removing the sample mean for each of the 16 experimental conditions. Results are presented in Table 6.

At the between-person level, mean complexity and discomfort ratings were strongly positively correlated; participants who generally perceived the images as more complex also tended to report greater discomfort. Average preference, however, was not reliably related to either average complexity or average discomfort.

At the within-person level, ratings of complexity were positively associated with discomfort, and ratings of discomfort were negatively associated with preference. Thus, relative to their own average responses, participants tended to report greater discomfort and lower preference for the same conditions. There was no significant within-person association between complexity and preference.

After removing the average effects of image-type × fractal scaling condition, the complexity–discomfort association remained positive but was reduced. This likely indicates that the association between complexity and discomfort partly reflected their shared sensitivity to experimental conditions. By comparison, the negative association between discomfort and preference remained largely unchanged, indicating participant-specific covariation beyond the average effects of image type and fractal scaling.

## 5. Discussion

The present study examined how spectral scaling and image representation shape perceived complexity, visual discomfort, and preference, and whether these relationships vary with self-reported autistic-like traits, ADHD-like traits, and sensory sensitivity. The spectral slope manipulation produced the clearest differences in perceived complexity, a substantial but smaller change in discomfort, and only a modest change in preference. Image type influenced all three judgments, and it moderated the strength of the spectral slope effect. The individual difference measures were more consistently associated with changes in ratings across spectral slope than with overall rating levels. These moderation effects were small, but they suggest that individual differences may be expressed as variation in perceptual and affective tuning to image statistics rather than as a general tendency to assign higher or lower ratings.

### 5.1. The Role of Stimulus-Driven Factors in Rated Complexity, Discomfort and Preference

As in our previous studies [9,10], images with increasing spectral slope alpha and reduced relative contribution of fine-scale image structure were reliably rated as less complex across all image types. This is a strong manipulation check in that it confirms that the accompanying changes in discomfort and preference occurred across a stimulus continuum that participants clearly discriminated in terms of apparent complexity.

The interaction between spectral slope and image type further demonstrates the role of stimulus-driven effects in complexity and affective judgments. Binarization and edge extraction alter the distribution and visibility of contours, local contrast, the absence of blur, and fine-scale structure, all of which interact with the nominally identical spectral manipulation of the seed grayscale images. Visual discomfort decreased as spectral slopes became steeper, and this reduction was particularly pronounced for edge images. Edge and thresholded images were more uncomfortable overall than grayscale and mountain images, and the decrease in discomfort across the spectral slope range was largest for edge images. This pattern indicates that spectral scaling was an important contributor to discomfort, but that specific image features and contour structure also played a role.

Preference was clearly differentiated from both complexity and discomfort. The spectral slope effect on preference was small, and ratings plateaued at the two steepest slopes. Grayscale images were preferred overall, but the preference function across spectral slope was broadly similar for the four image types. Comparison between preference and discomfort ratings, especially at the two steepest slopes, indicates that lower discomfort ratings did not map one-to-one onto greater liking, consistent with prior behavioral evidence that liking and discomfort are related but separable affective responses [2,6,15].

The present findings are broadly consistent with previous research linking visual discomfort to low-level image statistics, including blur and deviations from the image properties typically found in natural scenes [2,6,7,13,14,29]. However, direct comparisons between the current findings and earlier studies are not straightforward. For example, O’hare and Hibbard [29] reported a strong association between perceived blur and discomfort ratings for both synthetic and natural images. In the present study, the grayscale stimuli at the highest fractal scaling values contained visible blur and were rated as more uncomfortable than the edge and mountain images. However, discomfort ratings did not differ between the grayscale and thresholded images, despite the absence of blur in the latter. This pattern likely reflects the broader range of stimulus characteristics sampled in the current study and suggests that discomfort is influenced by multiple image features in addition to blur, such as contrast or excess energy at particular spatial frequencies.

Similarly, Juricevic et al. [6] reported a non-monotonic relationship between discomfort and spectral scaling, whereas the relationship observed in the present study was linear. This apparent discrepancy likely reflects differences in the range of fractal scaling values examined across the two studies. Specifically, our study sampled a narrower range of scaling variations than Juricevic et al. [6], potentially capturing only an approximately linear segment of a broader non-monotonic function. Comparisons with previous laboratory-based studies should also be made with caution because data in the present study were collected online without control over viewing conditions. Furthermore, our sample was relatively small and may not have been fully representative of the broader population, potentially limiting the generalizability of the findings and the reliability of the observed individual difference effects.

### 5.2. Individual Differences in Perceptual and Affective Tuning

The effects of individual difference measures were small and indicate modest, fractal scaling-specific differences in perceptual and affective ratings rather than a consistent tendency for participants with higher questionnaire scores to assign uniformly higher or lower ratings. Because the effects were small and the questionnaire measures were correlated, the specific response profiles should be interpreted cautiously.

For perceived complexity, all four individual difference measures showed small associations that varied across fractal scaling level or physical image complexity. Participants with higher CATI scores exhibited greater differentiation between the images with the shallowest (α = 0.75; most complex) and the steepest (α = 2.25; least complex) slopes compared with participants with lower scores. Participants with higher CHYPS-V scores exhibited the opposite pattern, with lower complexity ratings for physically more complex images and higher ratings for the least physically complex images. Higher ASRS scores were associated with greater perceived complexity at all but the shallowest (α = 0.75; most complex) fractal scaling slope, whereas higher SPQ-S scores—which indicate fewer sensory difficulties—were associated with lower perceived complexity at all but the steepest (α = 2.25; least complex) fractal scaling.

For discomfort, higher CATI scores were associated with less discomfort at the two steepest slopes (α = 1.75 and 2.25), whereas higher ASRS and SPQ-S scores were associated with greater discomfort at those same slopes. Higher CHYPS-V scores were associated with greater discomfort at α = 1.25, 1.75, and 2.25.

For preference, the effects were more selective. Higher ASRS scores were associated with a greater liking of the most physically complex images but a lower liking of physically less complex images. Higher CHYPS-V scores were associated with a lower liking of physically more complex images but a greater liking of the least physically complex images. Higher CATI scores were associated with a greater preference only at α = 1.75, and SPQ-S did not reliably moderate preference across the physical complexity continuum.

### 5.3. Relationships Among Complexity, Discomfort and Preference Ratings

The correlational analyses further clarify how perceived complexity, discomfort, and preference are related. At the between-person level, participants who generally perceived the images as more complex also tended to report greater discomfort. Within participants, conditions rated as more complex than usual were likewise rated as more uncomfortable, whereas conditions producing greater-than-usual discomfort were rated as less preferable. After removing the shared mean effects of each image type × spectral slope condition, the complexity–discomfort correlation was substantially reduced, indicating that much of their association reflected their common sensitivity to the experimental manipulation, although a smaller participant-specific relationship remained.

In contrast, the discomfort preference correlation remained virtually unchanged, suggesting participant-specific covariation beyond mean condition effects. Together, these findings further support the conclusion that discomfort and preference judgments are related but not interchangeable.

### 5.4. Limitations

Several limitations should be considered when interpreting these findings. The sample was relatively small, predominantly female, and drawn from a young university population, while autistic-like, ADHD-like, and sensory traits were assessed using self-report measures rather than clinically characterized groups; generalization to broader populations should therefore be cautious. In addition, this study was conducted online using participants’ own computers, without control over viewing distance, display settings, or ambient lighting, which may have introduced variability into responses to the low-level image properties. The individual difference effects were also small, and correlations among the trait measures limit construct-specific interpretations. Future studies with larger and more diverse samples, controlled viewing conditions, and broader stimulus sets would help establish the robustness and generalizability of these effects.

## 6. Conclusions

This study demonstrates that visual complexity, discomfort and preference judgments are shaped by a combination of image statistics and spatial structural characteristics, with fractal scaling exerting a particularly strong influence on perceived complexity and visual discomfort. Increasing spectral slope consistently reduced both complexity and discomfort across image types, whereas preference showed a weaker non-linear relationship with image structure. Although individual difference measures were associated with only small effects, they predominantly moderated responses to changes in spectral slope rather than overall rating levels, suggesting that autistic-like traits, ADHD-like traits, and aspects of sensory sensitivity may influence how observers respond to variations in image statistics.

Correlational analyses further demonstrated that complexity, discomfort, and preference are related but dissociable aspects of visual experience. While complexity and discomfort were strongly associated with both between- and within-participants, condition adjustment substantially reduced their association, indicating that a substantial component of their association seems to reflect shared sensitivity to the experimental conditions, although a smaller participant-specific relationship remained. In contrast, the relationship between discomfort and preference remained evident even after controlling for mean condition effects, suggesting a degree of participant-specific covariation.

Together, these findings support the view that complexity, discomfort, and preference are related but with distinct dimensions of visual experience, arising from an interaction between stimulus characteristics and individual perceptual sensitivities. Future research using larger, more representative population samples and more precisely characterized neurodivergent groups, combined with carefully controlled viewing conditions, will be important for clarifying the mechanisms underlying these relationships and determining their relevance for understanding individual differences in visual processing.

## Figures and Tables

**Table 1:** Mean scores, standard deviation and score range for the used questionnaire measures.

	Mean	SD	95% CI of Mean	Range (Possible Range)
CATI	128.5	26.62	[122.9, 134.1]	54–192 (42–210)
ASRS	33.96	10.54	[31.73, 36.18]	12–66 (0–72)
CHYPS-V	16.63	12.27	[14.05, 19.21]	0–53 (0–60)
SPQ-Short	48.88	10.11	[46.75, 51.01]	22–69 (0–105)

**Table 2:** Bivariate correlations between questionnaire measures.

Questionnaire	CATI	ASRS	CHYPS-V	SPQ-Short
CATI				
ASRS	0.55 *** [0.38, 0.68]			
CHYPS-V	0.53 *** [0.36, 0.66]	0.41 *** [0.22, 0.57]		
SPQ-Short	−0.26 * [−0.45, −0.06]	−0.31 ** [−0.49, −0.11]	−0.50 *** [−0.64, −0.32]	

Note. * *p* < 0.05, ** *p* < 0.01, *** *p* < 0.001.

**Table 3:** Simple slopes of individual difference measures predicting complexity ratings at each level of fractal scaling.

Fractal Scaling	CATI	CHYPS-V	ASRS	SPQ-S
0.75	0.145 [0.046, 0.245]	−0.191 [−0.291, −0.090]	0.068 [−0.026, 0.161]	−0.185 [−0.274,−0.096]
1.25	0.030 [−0.069, 0.130]	−0.140 [−0.240, −0.040]	0.141 [0.048, 0.235]	−0.101 [−0.190, −0.012]
1.75	−0.193 [−0.292, −0.093]	−0.030 [−0.130, 0.070]	0.282 [0.188, 0.376]	−0.172 [−0.261, −0.083]
2.25	−0.130 [−0.230, −0.030]	0.101 [0.001, 0.201]	0.129 [0.035, 0.222]	−0.009 [−0.098, 0.080]

**Table 4:** Simple slopes of individual difference measures predicting discomfort ratings at each level of fractal scaling.

Fractal Scaling	CATI	CHYPS-V	ASRS	SPQ-S
0.75	−0.018 [−0.147, 0.111]	0.118 [−0.012, 0.247]	−0.014 [−0.135, 0.107]	−0.061 [−0.176, 0.054]
1.25	−0.015 [−0.144, 0.114]	0.210 [0.080, 0.339]	0.090 [−0.031, 0.212]	0.100 [−0.015, 0.215]
1.75	−0.298 [−0.427, −0.169]	0.377 [0.247, 0.506]	0.247 [0.125, 0.368]	0.143 [0.028, 0.258]
2.25	−0.316 [−0.445, −0.187]	0.328 [0.199, 0.458]	0.286 [0.165, 0.407]	0.168 [0.053, 0.283]

**Table 5:** Simple slopes of individual difference measures predicting preference ratings at each level of fractal scaling.

Fractal Scaling	CATI	CHYPS-V	ASRS	SPQ-S
0.75	−0.038 [−0.170, 0.093]	−0.186 [−0.318, −0.054]	0.145 [0.021, 0.268]	−0.018 [−0.135, 0.099]
1.25	0.077 [−0.054, 0.209]	−0.164 [−0.295, −0.032]	0.015 [−0.108, 0.138]	−0.155 [−0.272, −0.038]
1.75	0.220 [0.089, 0.351]	0.074 [−0.057, 0.206]	−0.219 [−0.342, −0.096]	−0.029 [−0.146, 0.088]
2.25	0.122 [−0.009, 0.253]	0.138 [0.006, 0.270]	−0.248 [−0.372, −0.125]	−0.104 [−0.221, 0.013]

**Table 6:** Correlations among complexity, discomfort, and preference ratings at different levels of analysis.

Rating Pairs	Between-Person r [95% CI]	Within-Person r [95% CI]	Condition-Adjusted Within-Person r [95% CI]
Complexity—Discomfort	0.62 *** [0.48, 0.74]	0.58 *** [0.47, 0.67]	0.22 *** [0.10, 0.34]
Complexity—Preference	0.23 * [0.03, 0.42]	−0.11 [−0.25, 0.04]	0.01 [−0.10, 0.12]
Discomfort—Preference	0.19 [−0.02, 0.38]	−0.44 *** [−0.55, −0.31]	−0.43 *** [−0.54, −0.31]

Note: * *p* < 0.05, *** *p* < 0.001. Values are Pearson correlation coefficients with 95% confidence intervals. Between-person correlations were calculated from participant means across the 16 experimental conditions (*N* = 89), with confidence intervals based on Fisher’s *z* transformation.

## Data Availability

The data presented in this study are openly available in OSF at https://osf.io/26wmf/overview (accessed on 10 September 2026).

## Supplementary Materials

The following supporting information can be downloaded at: https://www.mdpi.com/article/10.3390/vision10040085/s1, Section S1: Complexity Ratings: Initial LME Model; Section S2: Complexity Ratings: LME Model with Trial Number and Repeated Instances Fixed Effects; Section S3: Complexity Ratings: LME Model with Covariates; Section S4: Discomfort Ratings: Initial LME Model; Section S5: Discomfort Ratings: LME Model with Trial Number and Repeated Instances Fixed Effects; Section S6: Discomfort Ratings: LME Model with Covariates; Section S7: Preference Ratings: Initial LME Model; Section S8: Preference Ratings: LME Model with Trial Number and Repeated Instances Fixed Effects; Section S9: Preference Ratings: LME Model with Covariates.
