# Supplementary material for: Individual Differences in Perceived Complexity, Visual Discomfort, and Preference for Images Varying in Spectral Scaling
Source: Vision (Basel). 2026 Oct 21;10(4):85. doi: 10.3390/vision10040085 (PMC13709420; doi:10.3390/vision10040085)
Supplement: Supplementary file 1 [file vision-10-00085-s001.zip › vision-4585449-Supplementary.pdf]

Section S1: Complexity Ratings: Initial LME Model

Model Info

Info		
Model Type	Mixed Model	Linear Mixed model for continuous y
Model	lmer	Complexity ~ 1 + `Fractal Scaling` + ImageType + `Fractal Scaling`:ImageType + (1 | ParticipantID) + (1 | Trial Image)
Distribution	Gaussian	Normal distribution of residuals
Direction	y	Dependend variable scores
Optimizer	bobyqa	
DF method	Satterthwaite	
Sample size	4272	
Converged	yes	
Y transform	none	
C.I. method	Wald	

Model Results

Model Fit

Type	R^2	df	LRT X^2	p
Conditional	0.667	17	4419.517	<.001
Marginal	0.545	15	233.683	<.001

Fixed Effects Omnibus Tests

	F	df	df (res)	p
Fractal Scaling	1272.233	3	32.000	<.001
ImageType	165.075	3	32.000	<.001
Fractal Scaling ✻ ImageType	39.870	9	32.000	<.001

Parameter Estimates (Fixed coefficients)

Names	Effect	Estimate	SE	95% Confidence Intervals Lower	Upper	df	t	p
(Intercept)	(Intercept)	6.154	0.109	5.941	6.367	78.385	56.643	<.001
Fractal Scaling1	1.25 - 0.75	-1.202	0.117	-1.432	-0.973	32.000	-10.274	<.001
Fractal Scaling2	1.75 - 0.75	-3.517	0.117	-3.746	-3.287	32.000	-30.055	<.001
Fractal Scaling3	2.25 - 0.75	-4.697	0.117	-4.926	-4.467	32.000	-40.137	<.001
ImageType1	Grayscale - Edge	-1.045	0.117	-1.274	-0.816	32.000	-8.930	<.001
ImageType2	Mountain - Edge	-1.918	0.117	-2.147	-1.688	32.000	-16.388	<.001
ImageType3	Thresholded - Edge	-0.303	0.117	-0.533	-0.074	32.000	-2.593	.014
Fractal Scaling1 ✻ ImageType1	(1.25 - 0.75) ✻ (Grayscale - Edge)	0.199	0.165	-0.126	0.523	32.000	1.200	.239
Fractal Scaling2 ✻ ImageType1	(1.75 - 0.75) ✻ (Grayscale - Edge)	1.693	0.165	1.368	2.017	32.000	10.230	<.001
Fractal Scaling3 ✻ ImageType1	(2.25 - 0.75) ✻ (Grayscale - Edge)	2.360	0.165	2.035	2.684	32.000	14.258	<.001
Fractal Scaling1 ✻ ImageType2	(1.25 - 0.75) ✻ (Mountain - Edge)	0.558	0.165	0.234	0.882	32.000	3.372	.002
Fractal Scaling2 ✻ ImageType2	(1.75 - 0.75) ✻ (Mountain - Edge)	1.727	0.165	1.402	2.051	32.000	10.434	<.001
Fractal Scaling3 ✻ ImageType2	(2.25 - 0.75) ✻ (Mountain - Edge)	2.030	0.165	1.706	2.354	32.000	12.267	<.001
Fractal Scaling1 ✻ ImageType3	(1.25 - 0.75) ✻ (Thresholded - Edge)	0.472	0.165	0.147	0.796	32.000	2.852	.008
Fractal Scaling2 ✻ ImageType3	(1.75 - 0.75) ✻ (Thresholded - Edge)	1.315	0.165	0.990	1.639	32.000	7.944	<.001
Fractal Scaling3 ✻ ImageType3	(2.25 - 0.75) ✻ (Thresholded - Edge)	1.135	0.165	0.810	1.459	32.000	6.858	<.001

Random Components

Groups	Name	Variance	SD	ICC
ParticipantID	(Intercept)	0.441	0.664	0.265
Trial Image	(Intercept)	0.007	0.082	0.006
Residual		1.222	1.106	

Note. Number of Obs: 4272 , Number of groups: ParticipantID 89, Trial Image 48

Post Hoc Tests

Post Hoc comparison: Fractal Scaling

Comparison Fractal Scaling	vs	Fractal Scaling	Difference	SE	95% Confidence Intervals Lower	Upper	t	df	$p_{bonferroni}$	p_{holm}
0.75	-	1.25	0.895	0.059	0.737	1.054	15.299	32.000	<.001	<.001
0.75	-	1.75	2.333	0.059	2.175	2.492	39.881	32.000	<.001	<.001
0.75	-	2.25	3.316	0.059	3.157	3.474	56.669	32.000	<.001	<.001
1.25	-	1.75	1.438	0.059	1.280	1.597	24.582	32.000	<.001	<.001
1.25	-	2.25	2.420	0.059	2.262	2.579	41.369	32.000	<.001	<.001
1.75	-	2.25	0.982	0.059	0.824	1.141	16.788	32.000	<.001	<.001

Post Hoc comparison: ImageType

Comparison					95% Confidence Intervals					
ImageType	vs	ImageType	Difference	SE	Lower	Upper	t	df	$p_{bonferroni}$	p_{holm}
Edge	-	Grayscale	-0.018	0.059	-0.176	0.141	-0.304	32.000	1.000	.763
Edge	-	Mountain	0.839	0.059	0.680	0.997	14.339	32.000	<.001	<.001
Edge	-	Thresholded	-0.427	0.059	-0.585	-0.268	-7.298	32.000	<.001	<.001
Grayscale	-	Mountain	0.857	0.059	0.698	1.015	14.643	32.000	<.001	<.001
Grayscale	-	Thresholded	-0.409	0.059	-0.568	-0.251	-6.994	32.000	<.001	<.001
Mountain	-	Thresholded	-1.266	0.059	-1.424	-1.107	-21.637	32.000	<.001	<.001

Post Hoc comparison: Fractal Scaling ✻ ImageType

Comparison							95% Confidence Intervals					
Fractal Scaling	ImageType	vs	Fractal Scaling	ImageType	Difference	SE	Lower	Upper	t	df	$p_{bonferroni}$	p_{holm}
0.75	Edge	-	0.75	Grayscale	1.045	0.117	0.611	1.479	8.930	32.000	<.001	<.001
0.75	Edge	-	0.75	Mountain	1.918	0.117	1.484	2.352	16.388	32.000	<.001	<.001
0.75	Edge	-	0.75	Thresholded	0.303	0.117	-0.131	0.737	2.593	32.000	1.000	.145
0.75	Edge	-	1.25	Edge	1.202	0.117	0.768	1.636	10.274	32.000	<.001	<.001
0.75	Edge	-	1.25	Grayscale	2.049	0.117	1.615	2.483	17.508	32.000	<.001	<.001
0.75	Edge	-	1.25	Mountain	2.562	0.117	2.128	2.996	21.893	32.000	<.001	<.001
0.75	Edge	-	1.25	Thresholded	1.034	0.117	0.600	1.468	8.834	32.000	<.001	<.001
0.75	Edge	-	1.75	Edge	3.517	0.117	3.083	3.951	30.055	32.000	<.001	<.001
0.75	Edge	-	1.75	Grayscale	2.869	0.117	2.435	3.303	24.517	32.000	<.001	<.001
0.75	Edge	-	1.75	Mountain	3.708	0.117	3.274	4.142	31.687	32.000	<.001	<.001
0.75	Edge	-	1.75	Thresholded	2.506	0.117	2.072	2.940	21.413	32.000	<.001	<.001
0.75	Edge	-	2.25	Edge	4.697	0.117	4.263	5.131	40.137	32.000	<.001	<.001
0.75	Edge	-	2.25	Grayscale	3.382	0.117	2.948	3.816	28.902	32.000	<.001	<.001
0.75	Edge	-	2.25	Mountain	4.584	0.117	4.150	5.018	39.177	32.000	<.001	<.001
0.75	Edge	-	2.25	Thresholded	3.865	0.117	3.431	4.299	33.031	32.000	<.001	<.001
0.75	Grayscale	-	0.75	Mountain	0.873	0.117	0.439	1.307	7.458	32.000	<.001	<.001
0.75	Grayscale	-	0.75	Thresholded	-0.742	0.117	-1.175	-0.308	-6.337	32.000	<.001	<.001
0.75	Grayscale	-	1.25	Edge	0.157	0.117	-0.277	0.591	1.344	32.000	1.000	1.000
0.75	Grayscale	-	1.25	Grayscale	1.004	0.117	0.570	1.438	8.578	32.000	<.001	<.001
0.75	Grayscale	-	1.25	Mountain	1.517	0.117	1.083	1.951	12.963	32.000	<.001	<.001
0.75	Grayscale	-	1.25	Thresholded	-0.011	0.117	-0.445	0.423	-0.096	32.000	1.000	1.000
0.75	Grayscale	-	1.75	Edge	2.472	0.117	2.038	2.906	21.125	32.000	<.001	<.001
0.75	Grayscale	-	1.75	Grayscale	1.824	0.117	1.390	2.258	15.587	32.000	<.001	<.001
0.75	Grayscale	-	1.75	Mountain	2.663	0.117	2.229	3.097	22.757	32.000	<.001	<.001
0.75	Grayscale	-	1.75	Thresholded	1.461	0.117	1.027	1.895	12.483	32.000	<.001	<.001
0.75	Grayscale	-	2.25	Edge	3.652	0.117	3.218	4.086	31.207	32.000	<.001	<.001
0.75	Grayscale	-	2.25	Grayscale	2.337	0.117	1.903	2.771	19.972	32.000	<.001	<.001
0.75	Grayscale	-	2.25	Mountain	3.539	0.117	3.105	3.973	30.247	32.000	<.001	<.001
0.75	Grayscale	-	2.25	Thresholded	2.820	0.117	2.386	3.254	24.101	32.000	<.001	<.001
0.75	Mountain	-	0.75	Thresholded	-1.614	0.117	-2.048	-1.180	-13.795	32.000	<.001	<.001
0.75	Mountain	-	1.25	Edge	-0.715	0.117	-1.149	-0.281	-6.113	32.000	<.001	<.001
0.75	Mountain	-	1.25	Grayscale	0.131	0.117	-0.303	0.565	1.120	32.000	1.000	1.000
0.75	Mountain	-	1.25	Mountain	0.644	0.117	0.210	1.078	5.505	32.000	<.001	<.001
0.75	Mountain	-	1.25	Thresholded	-0.884	0.117	-1.318	-0.450	-7.554	32.000	<.001	<.001
0.75	Mountain	-	1.75	Edge	1.599	0.117	1.165	2.033	13.667	32.000	<.001	<.001
0.75	Mountain	-	1.75	Grayscale	0.951	0.117	0.517	1.385	8.130	32.000	<.001	<.001
0.75	Mountain	-	1.75	Mountain	1.790	0.117	1.356	2.224	15.299	32.000	<.001	<.001
0.75	Mountain	-	1.75	Thresholded	0.588	0.117	0.154	1.022	5.025	32.000	.002	<.001
0.75	Mountain	-	2.25	Edge	2.779	0.117	2.345	3.213	23.749	32.000	<.001	<.001
0.75	Mountain	-	2.25	Grayscale	1.464	0.117	1.031	1.898	12.515	32.000	<.001	<.001
0.75	Mountain	-	2.25	Mountain	2.667	0.117	2.233	3.101	22.789	32.000	<.001	<.001
0.75	Mountain	-	2.25	Thresholded	1.948	0.117	1.514	2.381	16.644	32.000	<.001	<.001
0.75	Thresholded	-	1.25	Edge	0.899	0.117	0.465	1.333	7.682	32.000	<.001	<.001
0.75	Thresholded	-	1.25	Grayscale	1.745	0.117	1.311	2.179	14.915	32.000	<.001	<.001
0.75	Thresholded	-	1.25	Mountain	2.258	0.117	1.825	2.692	19.300	32.000	<.001	<.001

Post Hoc comparison: Fractal Scaling ✻ ImageType

0.75	Thresholded	-	1.25	Thresholded	0.730	0.117	0.296	1.164	6.241	32.000	<.001	<.001
0.75	Thresholded	-	1.75	Edge	3.213	0.117	2.780	3.647	27.462	32.000	<.001	<.001
0.75	Thresholded	-	1.75	Grayscale	2.566	0.117	2.132	2.999	21.925	32.000	<.001	<.001
0.75	Thresholded	-	1.75	Mountain	3.404	0.117	2.971	3.838	29.095	32.000	<.001	<.001
0.75	Thresholded	-	1.75	Thresholded	2.202	0.117	1.768	2.636	18.820	32.000	<.001	<.001
0.75	Thresholded	-	2.25	Edge	4.393	0.117	3.959	4.827	37.544	32.000	<.001	<.001
0.75	Thresholded	-	2.25	Grayscale	3.079	0.117	2.645	3.513	26.310	32.000	<.001	<.001
0.75	Thresholded	-	2.25	Mountain	4.281	0.117	3.847	4.715	36.584	32.000	<.001	<.001
0.75	Thresholded	-	2.25	Thresholded	3.562	0.117	3.128	3.996	30.439	32.000	<.001	<.001
1.25	Edge	-	1.25	Grayscale	0.846	0.117	0.413	1.280	7.234	32.000	<.001	<.001
1.25	Edge	-	1.25	Mountain	1.360	0.117	0.926	1.793	11.619	32.000	<.001	<.001
1.25	Edge	-	1.25	Thresholded	-0.169	0.117	-0.602	0.265	-1.440	32.000	1.000	1.000
1.25	Edge	-	1.75	Edge	2.315	0.117	1.881	2.749	19.780	32.000	<.001	<.001
1.25	Edge	-	1.75	Grayscale	1.667	0.117	1.233	2.101	14.243	32.000	<.001	<.001
1.25	Edge	-	1.75	Mountain	2.506	0.117	2.072	2.940	21.413	32.000	<.001	<.001
1.25	Edge	-	1.75	Thresholded	1.303	0.117	0.869	1.737	11.138	32.000	<.001	<.001
1.25	Edge	-	2.25	Edge	3.494	0.117	3.060	3.928	29.863	32.000	<.001	<.001
1.25	Edge	-	2.25	Grayscale	2.180	0.117	1.746	2.614	18.628	32.000	<.001	<.001
1.25	Edge	-	2.25	Mountain	3.382	0.117	2.948	3.816	28.902	32.000	<.001	<.001
1.25	Edge	-	2.25	Thresholded	2.663	0.117	2.229	3.097	22.757	32.000	<.001	<.001
1.25	Grayscale	-	1.25	Mountain	0.513	0.117	0.079	0.947	4.385	32.000	.014	.002
1.25	Grayscale	-	1.25	Thresholded	-1.015	0.117	-1.449	-0.581	-8.674	32.000	<.001	<.001
1.25	Grayscale	-	1.75	Edge	1.468	0.117	1.034	1.902	12.547	32.000	<.001	<.001
1.25	Grayscale	-	1.75	Grayscale	0.820	0.117	0.386	1.254	7.010	32.000	<.001	<.001
1.25	Grayscale	-	1.75	Mountain	1.659	0.117	1.225	2.093	14.179	32.000	<.001	<.001
1.25	Grayscale	-	1.75	Thresholded	0.457	0.117	0.023	0.891	3.905	32.000	.055	.007
1.25	Grayscale	-	2.25	Edge	2.648	0.117	2.214	3.082	22.629	32.000	<.001	<.001
1.25	Grayscale	-	2.25	Grayscale	1.333	0.117	0.899	1.767	11.395	32.000	<.001	<.001
1.25	Grayscale	-	2.25	Mountain	2.536	0.117	2.102	2.969	21.669	32.000	<.001	<.001
1.25	Grayscale	-	2.25	Thresholded	1.816	0.117	1.383	2.250	15.523	32.000	<.001	<.001
1.25	Mountain	-	1.25	Thresholded	-1.528	0.117	-1.962	-1.094	-13.059	32.000	<.001	<.001
1.25	Mountain	-	1.75	Edge	0.955	0.117	0.521	1.389	8.162	32.000	<.001	<.001
1.25	Mountain	-	1.75	Grayscale	0.307	0.117	-0.127	0.741	2.625	32.000	1.000	.145
1.25	Mountain	-	1.75	Mountain	1.146	0.117	0.712	1.580	9.794	32.000	<.001	<.001
1.25	Mountain	-	1.75	Thresholded	-0.056	0.117	-0.490	0.378	-0.480	32.000	1.000	1.000
1.25	Mountain	-	2.25	Edge	2.135	0.117	1.701	2.569	18.244	32.000	<.001	<.001
1.25	Mountain	-	2.25	Grayscale	0.820	0.117	0.386	1.254	7.010	32.000	<.001	<.001
1.25	Mountain	-	2.25	Mountain	2.022	0.117	1.589	2.456	17.284	32.000	<.001	<.001
1.25	Mountain	-	2.25	Thresholded	1.303	0.117	0.869	1.737	11.138	32.000	<.001	<.001
1.25	Thresholded	-	1.75	Edge	2.483	0.117	2.049	2.917	21.221	32.000	<.001	<.001
1.25	Thresholded	-	1.75	Grayscale	1.835	0.117	1.401	2.269	15.684	32.000	<.001	<.001
1.25	Thresholded	-	1.75	Mountain	2.674	0.117	2.240	3.108	22.853	32.000	<.001	<.001
1.25	Thresholded	-	1.75	Thresholded	1.472	0.117	1.038	1.906	12.579	32.000	<.001	<.001
1.25	Thresholded	-	2.25	Edge	3.663	0.117	3.229	4.097	31.303	32.000	<.001	<.001
1.25	Thresholded	-	2.25	Grayscale	2.348	0.117	1.914	2.782	20.068	32.000	<.001	<.001
1.25	Thresholded	-	2.25	Mountain	3.551	0.117	3.117	3.984	30.343	32.000	<.001	<.001
1.25	Thresholded	-	2.25	Thresholded	2.831	0.117	2.398	3.265	24.197	32.000	<.001	<.001
1.75	Edge	-	1.75	Grayscale	-0.648	0.117	-1.082	-0.214	-5.537	32.000	<.001	<.001
1.75	Edge	-	1.75	Mountain	0.191	0.117	-0.243	0.625	1.632	32.000	1.000	1.000

Post Hoc comparison: Fractal Scaling ✻ ImageType

1.75	Edge	-	1.75	Thresholded	-1.011	0.117	-1.445	-0.577	-8.642	32.000	<.001	<.001
1.75	Edge	-	2.25	Edge	1.180	0.117	0.746	1.614	10.082	32.000	<.001	<.001
1.75	Edge	-	2.25	Grayscale	-0.135	0.117	-0.569	0.299	-1.152	32.000	1.000	1.000
1.75	Edge	-	2.25	Mountain	1.067	0.117	0.634	1.501	9.122	32.000	<.001	<.001
1.75	Edge	-	2.25	Thresholded	0.348	0.117	-0.086	0.782	2.977	32.000	.662	.072
1.75	Grayscale	-	1.75	Mountain	0.839	0.117	0.405	1.273	7.170	32.000	<.001	<.001
1.75	Grayscale	-	1.75	Thresholded	-0.363	0.117	-0.797	0.071	-3.105	32.000	.476	.056
1.75	Grayscale	-	2.25	Edge	1.828	0.117	1.394	2.262	15.619	32.000	<.001	<.001
1.75	Grayscale	-	2.25	Grayscale	0.513	0.117	0.079	0.947	4.385	32.000	.014	.002
1.75	Grayscale	-	2.25	Mountain	1.715	0.117	1.281	2.149	14.659	32.000	<.001	<.001
1.75	Grayscale	-	2.25	Thresholded	0.996	0.117	0.562	1.430	8.514	32.000	<.001	<.001
1.75	Mountain	-	1.75	Thresholded	-1.202	0.117	-1.636	-0.768	-10.274	32.000	<.001	<.001
1.75	Mountain	-	2.25	Edge	0.989	0.117	0.555	1.423	8.450	32.000	<.001	<.001
1.75	Mountain	-	2.25	Grayscale	-0.326	0.117	-0.760	0.108	-2.785	32.000	1.000	.107
1.75	Mountain	-	2.25	Mountain	0.876	0.117	0.443	1.310	7.490	32.000	<.001	<.001
1.75	Mountain	-	2.25	Thresholded	0.157	0.117	-0.277	0.591	1.344	32.000	1.000	1.000
1.75	Thresholded	-	2.25	Edge	2.191	0.117	1.757	2.625	18.724	32.000	<.001	<.001
1.75	Thresholded	-	2.25	Grayscale	0.876	0.117	0.443	1.310	7.490	32.000	<.001	<.001
1.75	Thresholded	-	2.25	Mountain	2.079	0.117	1.645	2.513	17.764	32.000	<.001	<.001
1.75	Thresholded	-	2.25	Thresholded	1.360	0.117	0.926	1.793	11.619	32.000	<.001	<.001
2.25	Edge	-	2.25	Grayscale	-1.315	0.117	-1.749	-0.881	-11.235	32.000	<.001	<.001
2.25	Edge	-	2.25	Mountain	-0.112	0.117	-0.546	0.322	-0.960	32.000	1.000	1.000
2.25	Edge	-	2.25	Thresholded	-0.831	0.117	-1.265	-0.398	-7.106	32.000	<.001	<.001
2.25	Grayscale	-	2.25	Mountain	1.202	0.117	0.768	1.636	10.274	32.000	<.001	<.001
2.25	Grayscale	-	2.25	Thresholded	0.483	0.117	0.049	0.917	4.129	32.000	.029	.004
2.25	Mountain	-	2.25	Thresholded	-0.719	0.117	-1.153	-0.285	-6.145	32.000	<.001	<.001

Simple Effects

ANOVA for Simple Effects of ImageType

Moderator				
Fractal Scaling	F	Num df	Den df	p
0.75	106.851	3.000	32.000	<.001
1.25	75.732	3.000	32.000	<.001
1.75	45.769	3.000	32.000	<.001
2.25	56.333	3.000	32.000	<.001

Parameter Estimates for simple effects of ImageType

Moderator				95% Confidence Intervals				
Fractal Scaling	Effect	Estimate	SE	Lower	Upper	df	t	p
0.75	Grayscale - Edge	-1.045	0.117	-1.283	-0.807	32.000	-8.930	<.001
	Mountain - Edge	-1.918	0.117	-2.156	-1.679	32.000	-16.388	<.001
	Thresholded - Edge	-0.303	0.117	-0.542	-0.065	32.000	-2.593	.014
1.25	Grayscale - Edge	-0.846	0.117	-1.085	-0.608	32.000	-7.234	<.001
	Mountain - Edge	-1.360	0.117	-1.598	-1.121	32.000	-11.619	<.001
	Thresholded - Edge	0.169	0.117	-0.070	0.407	32.000	1.440	.159
1.75	Grayscale - Edge	0.648	0.117	0.410	0.886	32.000	5.537	<.001
	Mountain - Edge	-0.191	0.117	-0.429	0.047	32.000	-1.632	.112
	Thresholded - Edge	1.011	0.117	0.773	1.250	32.000	8.642	<.001
2.25	Grayscale - Edge	1.315	0.117	1.076	1.553	32.000	11.235	<.001
	Mountain - Edge	0.112	0.117	-0.126	0.351	32.000	0.960	.344
	Thresholded - Edge	0.831	0.117	0.593	1.070	32.000	7.106	<.001

Section S2: Complexity Ratings: LME Model with Trial Number and Repeated Instance Fixed Effects

Model Type	Mixed Model	Linear Mixed model for continuous y
Model	lmer	Complexity ~ 1 + \`Fractal Scaling\` + ImageType + TrialNumber + \`Repeated Instance\` + \`Fractal Scaling\`:ImageType + (1 + TrialNumber | ParticipantID) + (1 | Trial Image)
Distribution	Gaussian	Normal distribution of residuals
Direction	y	Dependend variable scores
Optimizer	bobyqa	
DF method	Satterthwaite	
Sample size	4272	
Converged	yes	
Y transform	none	
C.I. method	Wald	

Note. All covariates are centered to the mean

Model Results

Model Fit

Type	R^2	df	LRT X^2	p
Conditional	0.674	22	4448.990	<.001
Marginal	0.546	18	238.585	<.001

Fixed Effects Omnibus Tests

	F	df	df (res)	p
Fractal Scaling	1275.225	3	31.993	<.001
ImageType	165.619	3	31.944	<.001
TrialNumber	1.040	1	211.229	.309
Repeated Instance	2.340	2	4097.154	.096
Fractal Scaling ✻ ImageType	39.962	9	31.965	<.001

Parameter Estimates (Fixed coefficients)

Names	Effect	Estimate	SE	95% Confidence Intervals Lower	95% Confidence Intervals Upper	df	t	p
(Intercept)	(Intercept)	6.220	0.113	5.998	6.442	92.445	54.903	<.001
Fractal Scaling1	1.25 - 0.75	-1.203	0.117	-1.433	-0.974	31.957	-10.281	<.001
Fractal Scaling2	1.75 - 0.75	-3.527	0.117	-3.756	-3.297	31.894	-30.149	<.001
Fractal Scaling3	2.25 - 0.75	-4.693	0.117	-4.923	-4.464	31.933	-40.113	<.001
ImageType1	Grayscale - Edge	-1.041	0.117	-1.270	-0.812	31.909	-8.900	<.001
ImageType2	Mountain - Edge	-1.917	0.117	-2.146	-1.688	31.917	-16.385	<.001
ImageType3	Thresholded - Edge	-0.306	0.117	-0.535	-0.076	31.927	-2.613	.014
TrialNumber	TrialNumber	0.002	0.002	-0.002	0.006	211.229	1.020	.309
Repeated Instance1	2 - 1	-0.065	0.046	-0.156	0.026	4094.573	-1.409	.159
Repeated Instance2	3 - 1	-0.129	0.060	-0.246	-0.012	4106.200	-2.163	.031
Fractal Scaling1 ✻ ImageType1	(1.25 - 0.75) ✻ (Grayscale - Edge)	0.193	0.165	-0.131	0.517	31.953	1.166	.252
Fractal Scaling2 ✻ ImageType1	(1.75 - 0.75) ✻ (Grayscale - Edge)	1.702	0.166	1.377	2.027	32.062	10.276	<.001
Fractal Scaling3 ✻ ImageType1	(2.25 - 0.75) ✻ (Grayscale - Edge)	2.352	0.165	2.028	2.676	31.879	14.219	<.001
Fractal Scaling1 ✻ ImageType2	(1.25 - 0.75) ✻ (Mountain - Edge)	0.557	0.165	0.233	0.882	31.928	3.368	.002
Fractal Scaling2 ✻ ImageType2	(1.75 - 0.75) ✻ (Mountain - Edge)	1.742	0.165	1.418	2.067	31.936	10.530	<.001
Fractal Scaling3 ✻ ImageType2	(2.25 - 0.75) ✻ (Mountain - Edge)	2.007	0.166	1.682	2.331	31.980	12.122	<.001
Fractal Scaling1 ✻ ImageType3	(1.25 - 0.75) ✻ (Thresholded - Edge)	0.478	0.166	0.154	0.803	31.961	2.890	.007
Fractal Scaling2 ✻ ImageType3	(1.75 - 0.75) ✻ (Thresholded - Edge)	1.330	0.166	1.005	1.654	31.971	8.033	<.001
Fractal Scaling3 ✻ ImageType3	(2.25 - 0.75) ✻ (Thresholded - Edge)	1.124	0.165	0.800	1.449	31.924	6.795	<.001

Random Components

Groups	Name	Variance	SD	ICC
ParticipantID	(Intercept)	0.442	0.665	0.269
	TrialNumber	0.000	0.011	
Trial Image	(Intercept)	0.007	0.083	0.006
Residual		1.197	1.094	

Note. Number of Obs: 4272 , Number of groups: ParticipantID 89, Trial Image 48

Random Parameters correlations

Groups	param1	param2	Cov.	Corr.
ParticipantID	(Intercept)	TrialNumber	0.000	0.028

Post Hoc Tests

Post Hoc comparison: Fractal Scaling

Comparison					95% Confidence Intervals					
Fractal Scaling	vs	Fractal Scaling	Difference	SE	Lower	Upper	t	df	$p_{bonferroni}$	p_{holm}
0.75	-	1.25	0.896	0.059	0.737	1.055	15.313	31.957	<.001	<.001
0.75	-	1.75	2.333	0.059	2.174	2.492	39.876	31.940	<.001	<.001
0.75	-	2.25	3.323	0.059	3.164	3.481	56.749	32.040	<.001	<.001
1.25	-	1.75	1.437	0.059	1.278	1.596	24.554	31.979	<.001	<.001
1.25	-	2.25	2.427	0.059	2.268	2.585	41.458	32.003	<.001	<.001
1.75	-	2.25	0.990	0.059	0.831	1.148	16.904	32.039	<.001	<.001

Post Hoc comparison: ImageType

Comparison					95% Confidence Intervals					
ImageType	vs	ImageType	Difference	SE	Lower	Upper	t	df	$p_{bonferroni}$	p_{holm}
Edge	-	Grayscale	-0.021	0.059	-0.179	0.138	-0.352	31.946	1.000	.727
Edge	-	Mountain	0.840	0.059	0.682	0.999	14.356	31.990	<.001	<.001
Edge	-	Thresholded	-0.427	0.059	-0.586	-0.269	-7.302	31.978	<.001	<.001
Grayscale	-	Mountain	0.861	0.058	0.702	1.019	14.719	31.904	<.001	<.001
Grayscale	-	Thresholded	-0.407	0.058	-0.565	-0.248	-6.955	31.887	<.001	<.001
Mountain	-	Thresholded	-1.268	0.059	-1.426	-1.109	-21.663	31.958	<.001	<.001

Section S3: Complexity Ratings: LME Model with Covariates

Model Info

Info		
Model Type	Mixed Model	Linear Mixed model for continuous y
Model	lmer	Complexity ~ 1 + `Fractal Scaling` + ImageType + SPQ + CATI + CHYPS + ASRS + `Fractal Scaling`:ImageType + `Fractal Scaling`:SPQ + `Fractal Scaling`:CATI + `Fractal Scaling`:CHYPS + `Fractal Scaling`:ASRS + (1 | ParticipantID) + (1 | Trial Image)
Distribution	Gaussian	Normal distribution of residuals
Direction	y	Dependend variable scores
Optimizer	bobyqa	
DF method	Satterthwaite	
Sample size	4272	
Converged	yes	
Y transform	none	
C.I. method	Wald	

Note. All covariates are standardized

Model Results

Model Fit

Type	R^2	df	LRT X^2	p
Conditional	0.673	33	4489.516	<.001
Marginal	0.555	31	303.682	<.001

Additional Indices

Info	Model Value	Comment
LogLikelihood	-6642.391	
AIC	13354.781	Less is better
BIC	13577.375	Less is better

Fixed Effects Omnibus Tests

	F	df	df (res)	p
Fractal Scaling	1272.233	3	32.000	<.001
ImageType	165.075	3	32.000	<.001
SPQ	1.965	1	84.000	.165
CATI	0.156	1	84.000	.694
CHYPS	0.478	1	84.000	.491
ASRS	3.128	1	84.000	.081
Fractal Scaling ✻ ImageType	39.870	9	32.000	<.001
Fractal Scaling ✻ SPQ	4.268	3	4124.000	.005
Fractal Scaling ✻ CATI	12.234	3	4124.000	<.001
Fractal Scaling ✻ CHYPS	8.597	3	4124.000	<.001
Fractal Scaling ✻ ASRS	4.836	3	4124.000	.002

Parameter Estimates (Fixed coefficients)

				95% Confidence Intervals				
Names	Effect	Estimate	SE	Lower	Upper	df	t	p
(Intercept)	(Intercept)	3.701	0.072	3.559	3.843	87.235	51.085	<.001
Fractal Scaling1	1.25 - 0.75	-0.895	0.059	-1.010	-0.780	32.000	-15.299	<.001
Fractal Scaling2	1.75 - 0.75	-2.333	0.059	-2.448	-2.219	32.000	-39.881	<.001
Fractal Scaling3	2.25 - 0.75	-3.316	0.059	-3.430	-3.201	32.000	-56.669	<.001
ImageType1	Grayscale - Edge	0.018	0.059	-0.097	0.132	32.000	0.304	.763
ImageType2	Mountain - Edge	-0.839	0.059	-0.954	-0.724	32.000	-14.339	<.001
ImageType3	Thresholded - Edge	0.427	0.059	0.312	0.542	32.000	7.298	<.001
SPQ	SPQ	-0.117	0.083	-0.280	0.047	84.000	-1.402	.165
CATI	CATI	-0.037	0.093	-0.220	0.146	84.000	-0.395	.694
CHYPS	CHYPS	-0.065	0.094	-0.249	0.119	84.000	-0.692	.491
ASRS	ASRS	0.155	0.088	-0.017	0.327	84.000	1.769	.081
Fractal Scaling1 ✻ ImageType1	(1.25 - 0.75) ✻ (Grayscale - Edge)	0.199	0.165	-0.126	0.523	32.000	1.200	.239
Fractal Scaling2 ✻ ImageType1	(1.75 - 0.75) ✻ (Grayscale - Edge)	1.693	0.165	1.368	2.017	32.000	10.230	<.001
Fractal Scaling3 ✻ ImageType1	(2.25 - 0.75) ✻ (Grayscale - Edge)	2.360	0.165	2.035	2.684	32.000	14.258	<.001
Fractal Scaling1 ✻ ImageType2	(1.25 - 0.75) ✻ (Mountain - Edge)	0.558	0.165	0.234	0.882	32.000	3.372	.002
Fractal Scaling2 ✻ ImageType2	(1.75 - 0.75) ✻ (Mountain - Edge)	1.727	0.165	1.402	2.051	32.000	10.434	<.001
Fractal Scaling3 ✻ ImageType2	(2.25 - 0.75) ✻ (Mountain - Edge)	2.030	0.165	1.706	2.354	32.000	12.267	<.001
Fractal Scaling1 ✻ ImageType3	(1.25 - 0.75) ✻ (Thresholded - Edge)	0.472	0.165	0.147	0.796	32.000	2.852	.008
Fractal Scaling2 ✻ ImageType3	(1.75 - 0.75) ✻ (Thresholded - Edge)	1.315	0.165	0.990	1.639	32.000	7.944	<.001
Fractal Scaling3 ✻ ImageType3	(2.25 - 0.75) ✻ (Thresholded - Edge)	1.135	0.165	0.810	1.459	32.000	6.858	<.001
Fractal Scaling1 ✻ SPQ	(1.25 - 0.75) ✻ SPQ	0.084	0.055	-0.025	0.192	4124.000	1.514	.130
Fractal Scaling2 ✻ SPQ	(1.75 - 0.75) ✻ SPQ	0.013	0.055	-0.096	0.121	4124.000	0.227	.820
Fractal Scaling3 ✻ SPQ	(2.25 - 0.75) ✻ SPQ	0.176	0.055	0.068	0.285	4124.000	3.180	.001
Fractal Scaling1 ✻ CATI	(1.25 - 0.75) ✻ CATI	-0.115	0.062	-0.237	0.007	4124.000	-1.853	.064
Fractal Scaling2 ✻ CATI	(1.75 - 0.75) ✻ CATI	-0.338	0.062	-0.460	-0.216	4124.000	-5.448	<.001
Fractal Scaling3 ✻ CATI	(2.25 - 0.75) ✻ CATI	-0.275	0.062	-0.397	-0.154	4124.000	-4.436	<.001
Fractal Scaling1 ✻ CHYPS	(1.25 - 0.75) ✻ CHYPS	0.051	0.062	-0.071	0.173	4124.000	0.816	.415

Parameter Estimates (Fixed coefficients)

Fractal Scaling2 ✻ CHYPS	(1.75 - 0.75) ✻ CHYPS	0.160	0.062	0.038	0.283	4124.000	2.571	.010
Fractal Scaling3 ✻ CHYPS	(2.25 - 0.75) ✻ CHYPS	0.292	0.062	0.170	0.414	4124.000	4.677	<.001
Fractal Scaling1 ✻ ASRS	(1.25 - 0.75) ✻ ASRS	0.073	0.058	-0.041	0.188	4124.000	1.261	.208
Fractal Scaling2 ✻ ASRS	(1.75 - 0.75) ✻ ASRS	0.214	0.058	0.100	0.329	4124.000	3.677	<.001
Fractal Scaling3 ✻ ASRS	(2.25 - 0.75) ✻ ASRS	0.061	0.058	-0.053	0.175	4124.000	1.043	.297

Random Components

Groups	Name	Variance	SD	ICC
ParticipantID	(Intercept)	0.429	0.655	0.262
Trial Image	(Intercept)	0.007	0.084	0.006
Residual		1.207	1.099	

Note. Number of Obs: 4272 , Number of groups: ParticipantID 89, Trial Image 48

Estimated Marginal Means

Estimate Marginal Means - Fractal Scaling ✻ CATI

					95% Confidence Intervals	
Fractal Scaling	CATI	Mean	SE	df	Lower	Upper
0.75	Mean-1·SD	5.192	0.129	117.850	4.936	5.448
0.75	Mean	5.337	0.081	106.248	5.177	5.497
0.75	Mean+1·SD	5.482	0.129	117.850	5.227	5.738
1.25	Mean-1·SD	4.412	0.129	117.850	4.156	4.667
1.25	Mean	4.442	0.081	106.248	4.282	4.602
1.25	Mean+1·SD	4.472	0.129	117.850	4.217	4.728
1.75	Mean-1·SD	3.197	0.129	117.850	2.941	3.452
1.75	Mean	3.004	0.081	106.248	2.844	3.164
1.75	Mean+1·SD	2.811	0.129	117.850	2.555	3.067
2.25	Mean-1·SD	2.152	0.129	117.850	1.896	2.407
2.25	Mean	2.022	0.081	106.248	1.861	2.182
2.25	Mean+1·SD	1.892	0.129	117.850	1.636	2.147

Estimate Marginal Means - Fractal Scaling ✻ CHYPS

					95% Confidence Intervals	
Fractal Scaling	CHYPS	Mean	SE	df	Lower	Upper
0.75	Mean-1·SD	5.528	0.130	117.856	5.271	5.784
0.75	Mean	5.337	0.081	106.248	5.177	5.497
0.75	Mean+1·SD	5.146	0.130	117.856	4.890	5.403
1.25	Mean-1·SD	4.582	0.130	117.856	4.325	4.838
1.25	Mean	4.442	0.081	106.248	4.282	4.602
1.25	Mean+1·SD	4.302	0.130	117.856	4.046	4.559
1.75	Mean-1·SD	3.034	0.130	117.856	2.777	3.291
1.75	Mean	3.004	0.081	106.248	2.844	3.164
1.75	Mean+1·SD	2.974	0.130	117.856	2.717	3.230
2.25	Mean-1·SD	1.920	0.130	117.856	1.664	2.177
2.25	Mean	2.022	0.081	106.248	1.861	2.182
2.25	Mean+1·SD	2.123	0.130	117.856	1.866	2.379

Estimate Marginal Means - Fractal Scaling ✻ ASRS

					95% Confidence Intervals	
Fractal Scaling	ASRS	Mean	SE	df	Lower	Upper
0.75	Mean-1·SD	5.269	0.124	117.750	5.023	5.516
0.75	Mean	5.337	0.081	106.248	5.177	5.497
0.75	Mean+1·SD	5.405	0.124	117.750	5.158	5.651
1.25	Mean-1·SD	4.301	0.124	117.750	4.054	4.547
1.25	Mean	4.442	0.081	106.248	4.282	4.602
1.25	Mean+1·SD	4.583	0.124	117.750	4.337	4.830
1.75	Mean-1·SD	2.722	0.124	117.750	2.475	2.968
1.75	Mean	3.004	0.081	106.248	2.844	3.164
1.75	Mean+1·SD	3.286	0.124	117.750	3.039	3.532
2.25	Mean-1·SD	1.893	0.124	117.750	1.647	2.139
2.25	Mean	2.022	0.081	106.248	1.861	2.182
2.25	Mean+1·SD	2.150	0.124	117.750	1.904	2.396

Estimate Marginal Means - Fractal Scaling ✻ SPQ

					95% Confidence Intervals	
Fractal Scaling	SPQ	Mean	SE	df	Lower	Upper
0.75	Mean-1·SD	5.522	0.121	117.623	5.283	5.761
0.75	Mean	5.337	0.081	106.248	5.177	5.497
0.75	Mean+1·SD	5.152	0.121	117.623	4.913	5.392
1.25	Mean-1·SD	4.543	0.121	117.623	4.304	4.782
1.25	Mean	4.442	0.081	106.248	4.282	4.602
1.25	Mean+1·SD	4.341	0.121	117.623	4.102	4.580
1.75	Mean-1·SD	3.176	0.121	117.623	2.937	3.415
1.75	Mean	3.004	0.081	106.248	2.844	3.164
1.75	Mean+1·SD	2.832	0.121	117.623	2.592	3.071
2.25	Mean-1·SD	2.030	0.121	117.623	1.791	2.270
2.25	Mean	2.022	0.081	106.248	1.861	2.182
2.25	Mean+1·SD	2.013	0.121	117.623	1.774	2.252

Results Plots

Plot: Complexity ~ CATI ✻ Fractal Scaling

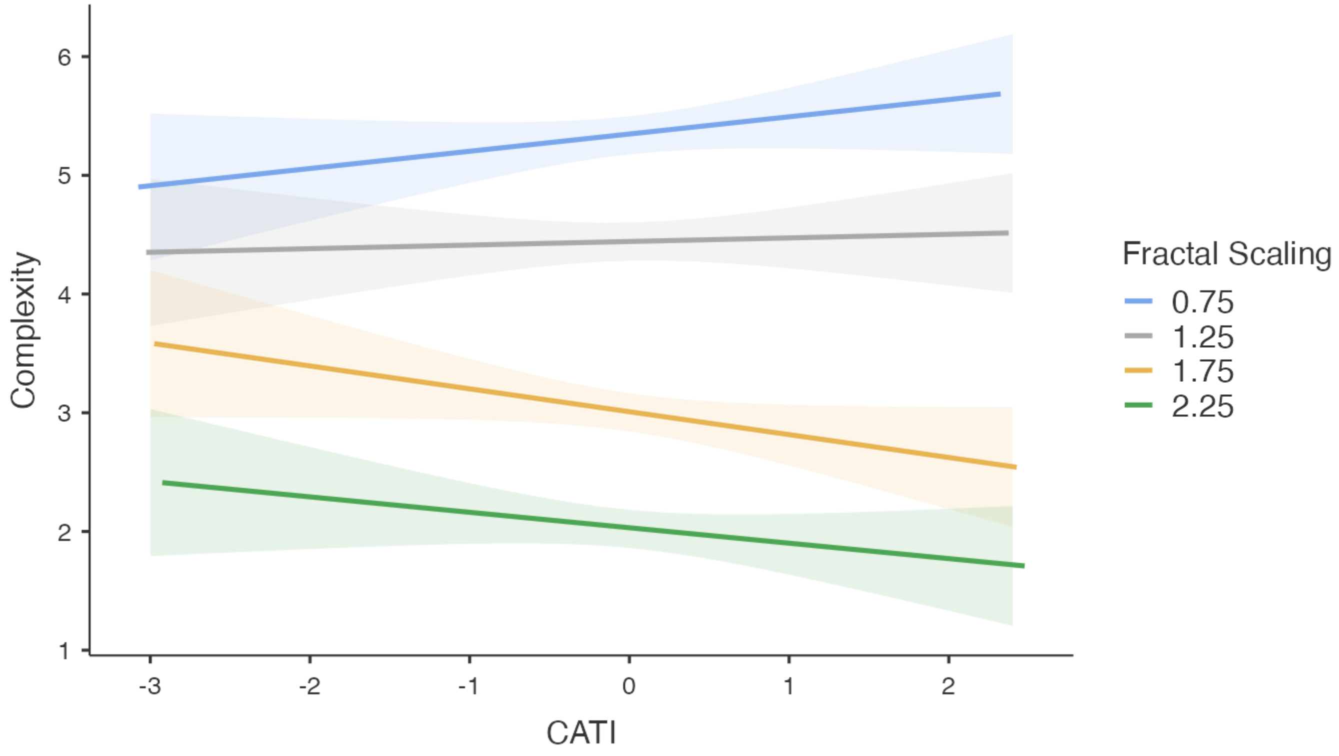

Plot: Complexity ~ CHYPS ✻ Fractal Scaling

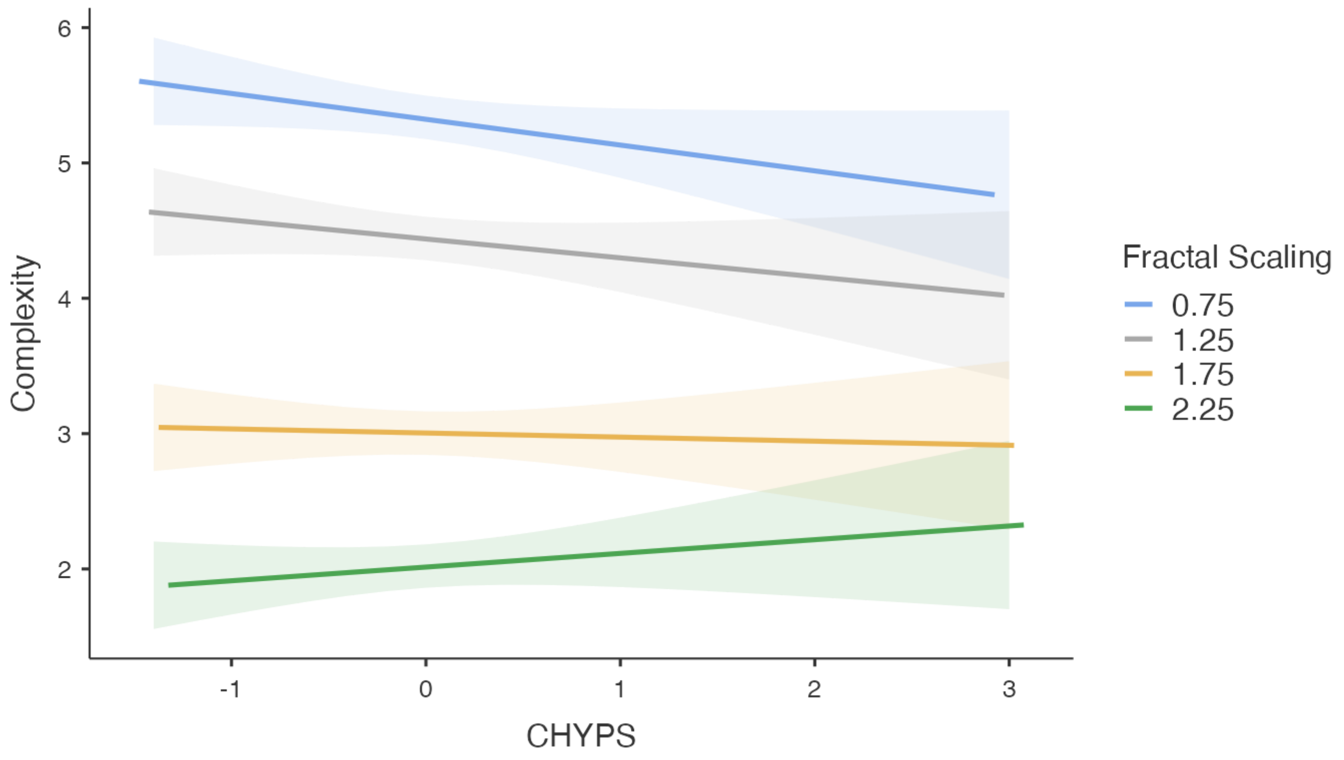

Plot: Complexity ~ ASRS ∗ Fractal Scaling

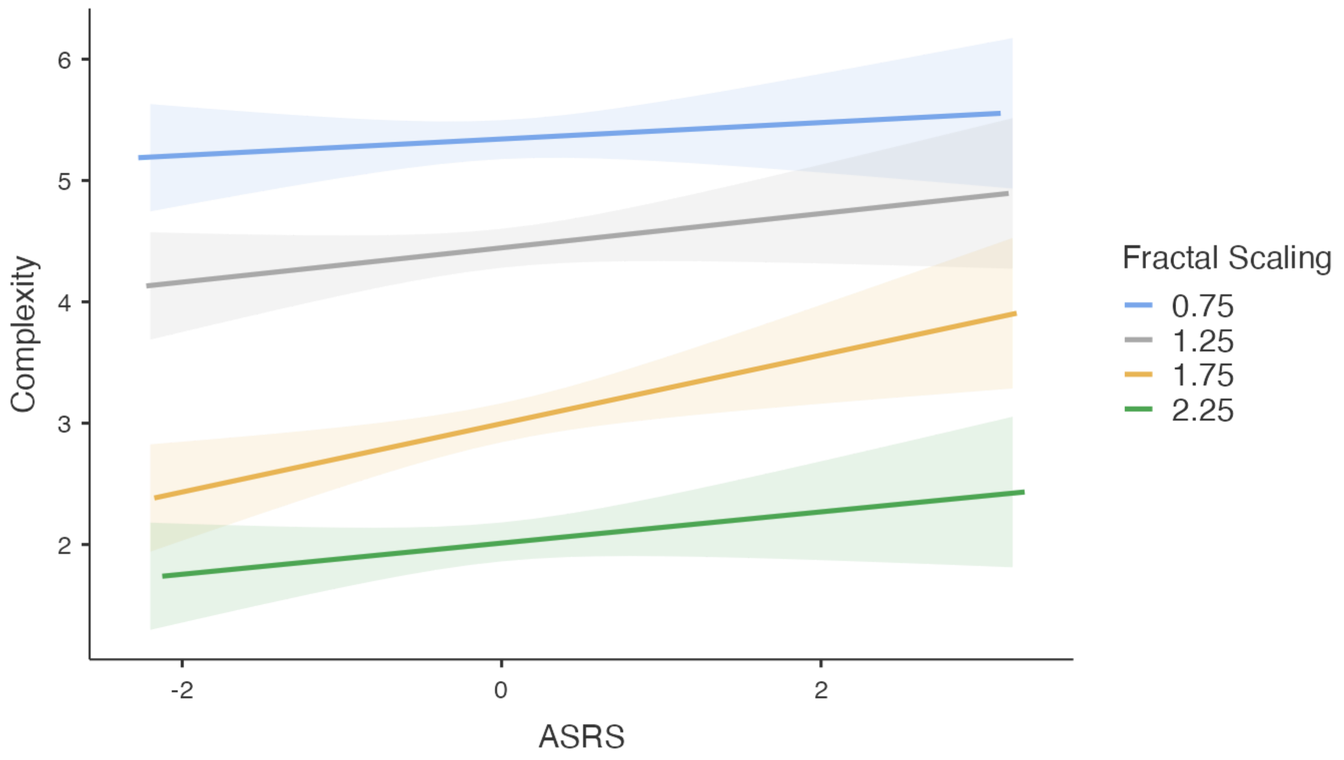

Plot: Complexity ~ SPQ ∗ Fractal Scaling

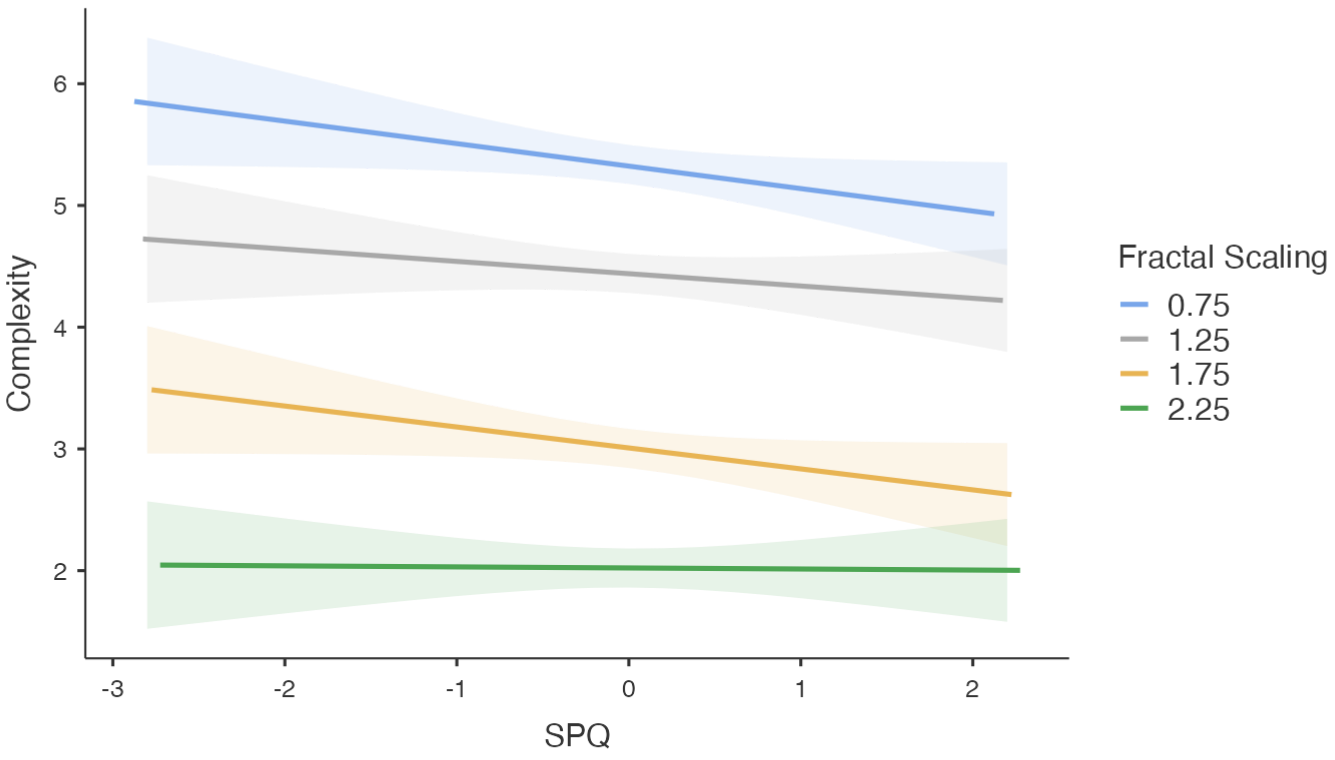

Complexity
6
5
4
3
2
-3
-2
-1
0
1
2
SPQ
Fractal Scaling
0.75
1.25
1.75
2.25

Section S4: Discomfort Ratings: Initial LME Model

Model Info

Info		
Model Type	Mixed Model	Linear Mixed model for continuous y
Model	lmer	Discomfort ~ 1 + DiscomfortImageType + DiscomfortScaling + DiscomfortScaling:DiscomfortImageType + (1 | ParticipantID) + (1 | DiscomfortImage)
Distribution	Gaussian	Normal distribution of residuals
Direction	y	Dependend variable scores
Optimizer	bobyqa	
DF method	Satterthwaite	
Sample size	4272	
Converged	yes	
Y transform	none	
C.I. method	Wald	

Model Results

Model Fit

Type	R^2	df	LRT X^2	p
Conditional	0.429	17	2124.832	<.001
Marginal	0.209	15	199.045	<.001

Fixed Effects Omnibus Tests

	F	df	df (res)	p
DiscomfortImageType	64.762	3	4168.000	<.001
DiscomfortScaling	399.981	3	4168.000	<.001
DiscomfortImageType ✻ DiscomfortScaling	18.579	9	4168.000	<.001

Parameter Estimates (Fixed coefficients)

				95% Confidence Intervals				
Names	Effect	Estimate	SE	Lower	Upper	df	t	p
(Intercept)	(Intercept)	5.004	0.128	4.754	5.254	272.059	39.232	<.001
DiscomfortImageType1	Grayscale - Edge	-1.213	0.123	-1.454	-0.973	4168.000	-9.879	<.001
DiscomfortImageType2	Mountain - Edge	-1.240	0.123	-1.481	-0.999	4168.000	-10.093	<.001
DiscomfortImageType3	Thresholded - Edge	-0.330	0.123	-0.570	-0.089	4168.000	-2.683	.007
DiscomfortScaling1	1.25 - 0.75	-0.850	0.123	-1.091	-0.609	4168.000	-6.921	<.001
DiscomfortScaling2	1.75 - 0.75	-2.333	0.123	-2.574	-2.093	4168.000	-18.996	<.001
DiscomfortScaling3	2.25 - 0.75	-2.880	0.123	-3.121	-2.639	4168.000	-23.448	<.001
DiscomfortImageType1 ✻ DiscomfortScaling1	(Grayscale - Edge) ✻ (1.25 - 0.75)	0.202	0.174	-0.138	0.543	4168.000	1.164	.244
DiscomfortImageType2 ✻ DiscomfortScaling1	(Mountain - Edge) ✻ (1.25 - 0.75)	0.300	0.174	-0.041	0.640	4168.000	1.725	.085
DiscomfortImageType3 ✻ DiscomfortScaling1	(Thresholded - Edge) ✻ (1.25 - 0.75)	0.150	0.174	-0.191	0.490	4168.000	0.862	.389
DiscomfortImageType1 ✻ DiscomfortScaling2	(Grayscale - Edge) ✻ (1.75 - 0.75)	1.348	0.174	1.008	1.689	4168.000	7.762	<.001
DiscomfortImageType2 ✻ DiscomfortScaling2	(Mountain - Edge) ✻ (1.75 - 0.75)	0.933	0.174	0.592	1.273	4168.000	5.369	<.001
DiscomfortImageType3 ✻ DiscomfortScaling2	(Thresholded - Edge) ✻ (1.75 - 0.75)	0.689	0.174	0.349	1.030	4168.000	3.967	<.001
DiscomfortImageType1 ✻ DiscomfortScaling3	(Grayscale - Edge) ✻ (2.25 - 0.75)	1.888	0.174	1.547	2.228	4168.000	10.866	<.001
DiscomfortImageType2 ✻ DiscomfortScaling3	(Mountain - Edge) ✻ (2.25 - 0.75)	1.090	0.174	0.749	1.430	4168.000	6.274	<.001
DiscomfortImageType3 ✻ DiscomfortScaling3	(Thresholded - Edge) ✻ (2.25 - 0.75)	0.910	0.174	0.570	1.251	4168.000	5.239	<.001

Random Components

Groups	Name	Variance	SD	ICC
ParticipantID	(Intercept)	0.776	0.881	0.278
DiscomfortImage	(Intercept)	0.000	0.000	0.000
Residual		2.014	1.419	

Note. Number of Obs: 4272 , Number of groups: ParticipantID 89, DiscomfortImage 48

Post Hoc Tests

Post Hoc comparison: DiscomfortImageType

Comparison								
DiscomfortImageType	vs	DiscomfortImageType	Difference	SE	t	df	$p_{bonferroni}$	p_{holm}
Edge	-	Grayscale	0.354	0.061	5.763	4168.000	<.001	<.001
Edge	-	Mountain	0.659	0.061	10.733	4168.000	<.001	<.001
Edge	-	Thresholded	-0.108	0.061	-1.753	4168.000	.478	.080
Grayscale	-	Mountain	0.305	0.061	4.970	4168.000	<.001	<.001
Grayscale	-	Thresholded	-0.462	0.061	-7.516	4168.000	<.001	<.001
Mountain	-	Thresholded	-0.767	0.061	-12.486	4168.000	<.001	<.001

Simple Effects

ANOVA for Simple Effects of DiscomfortScaling

Moderator				
DiscomfortImageType	F	Num df	Den df	p
Edge	232.878	3.000	4168.000	<.001
Grayscale	28.804	3.000	4168.000	<.001
Mountain	87.063	3.000	4168.000	<.001
Thresholded	106.972	3.000	4168.000	<.001

Parameter Estimates for simple effects of DiscomfortScaling

Moderator				95% Confidence Intervals				
DiscomfortImageType	Effect	Estimate	SE	Lower	Upper	df	t	p
Edge	1.25 - 0.75	-0.850	0.123	-1.091	-0.609	4168.000	-6.921	<.001
	1.75 - 0.75	-2.333	0.123	-2.574	-2.093	4168.000	-18.996	<.001
	2.25 - 0.75	-2.880	0.123	-3.121	-2.639	4168.000	-23.448	<.001
Grayscale	1.25 - 0.75	-0.648	0.123	-0.889	-0.407	4168.000	-5.275	<.001
	1.75 - 0.75	-0.985	0.123	-1.226	-0.744	4168.000	-8.019	<.001
	2.25 - 0.75	-0.993	0.123	-1.233	-0.752	4168.000	-8.080	<.001
Mountain	1.25 - 0.75	-0.551	0.123	-0.791	-0.310	4168.000	-4.482	<.001
	1.75 - 0.75	-1.401	0.123	-1.642	-1.160	4168.000	-11.404	<.001
	2.25 - 0.75	-1.790	0.123	-2.031	-1.549	4168.000	-14.575	<.001
Thresholded	1.25 - 0.75	-0.700	0.123	-0.941	-0.460	4168.000	-5.702	<.001
	1.75 - 0.75	-1.644	0.123	-1.885	-1.403	4168.000	-13.386	<.001
	2.25 - 0.75	-1.970	0.123	-2.211	-1.729	4168.000	-16.038	<.001

Estimated Marginal Means

Estimate Marginal Means - DiscomfortScaling

				95% Confidence Intervals	
DiscomfortScaling	Mean	SE	df	Lower	Upper
0.75	4.308	0.103	117.102	4.104	4.512
1.25	3.621	0.103	117.102	3.417	3.825
1.75	2.717	0.103	117.102	2.513	2.921
2.25	2.400	0.103	117.102	2.196	2.604

Estimate Marginal Means - DiscomfortImageType

				95% Confidence Intervals	
DiscomfortImageType	Mean	SE	df	Lower	Upper
Edge	3.488	0.103	117.102	3.284	3.692
Grayscale	3.134	0.103	117.102	2.930	3.338
Mountain	2.829	0.103	117.102	2.625	3.033
Thresholded	3.596	0.103	117.102	3.392	3.799

Estimate Marginal Means - DiscomfortScaling ✻ DiscomfortImageType

					95% Confidence Intervals	
DiscomfortScaling	DiscomfortImageType	Mean	SE	df	Lower	Upper
0.75	Edge	5.004	0.128	272.059	4.753	5.255
0.75	Grayscale	3.790	0.128	272.059	3.539	4.041
0.75	Mountain	3.764	0.128	272.059	3.513	4.015
0.75	Thresholded	4.674	0.128	272.059	4.423	4.925
1.25	Edge	4.154	0.128	272.059	3.902	4.405
1.25	Grayscale	3.142	0.128	272.059	2.891	3.393
1.25	Mountain	3.213	0.128	272.059	2.962	3.465
1.25	Thresholded	3.974	0.128	272.059	3.723	4.225
1.75	Edge	2.670	0.128	272.059	2.419	2.922
1.75	Grayscale	2.805	0.128	272.059	2.554	3.056
1.75	Mountain	2.363	0.128	272.059	2.112	2.614
1.75	Thresholded	3.030	0.128	272.059	2.779	3.281
2.25	Edge	2.124	0.128	272.059	1.873	2.375
2.25	Grayscale	2.798	0.128	272.059	2.547	3.049
2.25	Mountain	1.974	0.128	272.059	1.723	2.225
2.25	Thresholded	2.704	0.128	272.059	2.453	2.955

Results Plots

Plot: Discomfort ~ DiscomfortScaling ✻ DiscomfortImageType

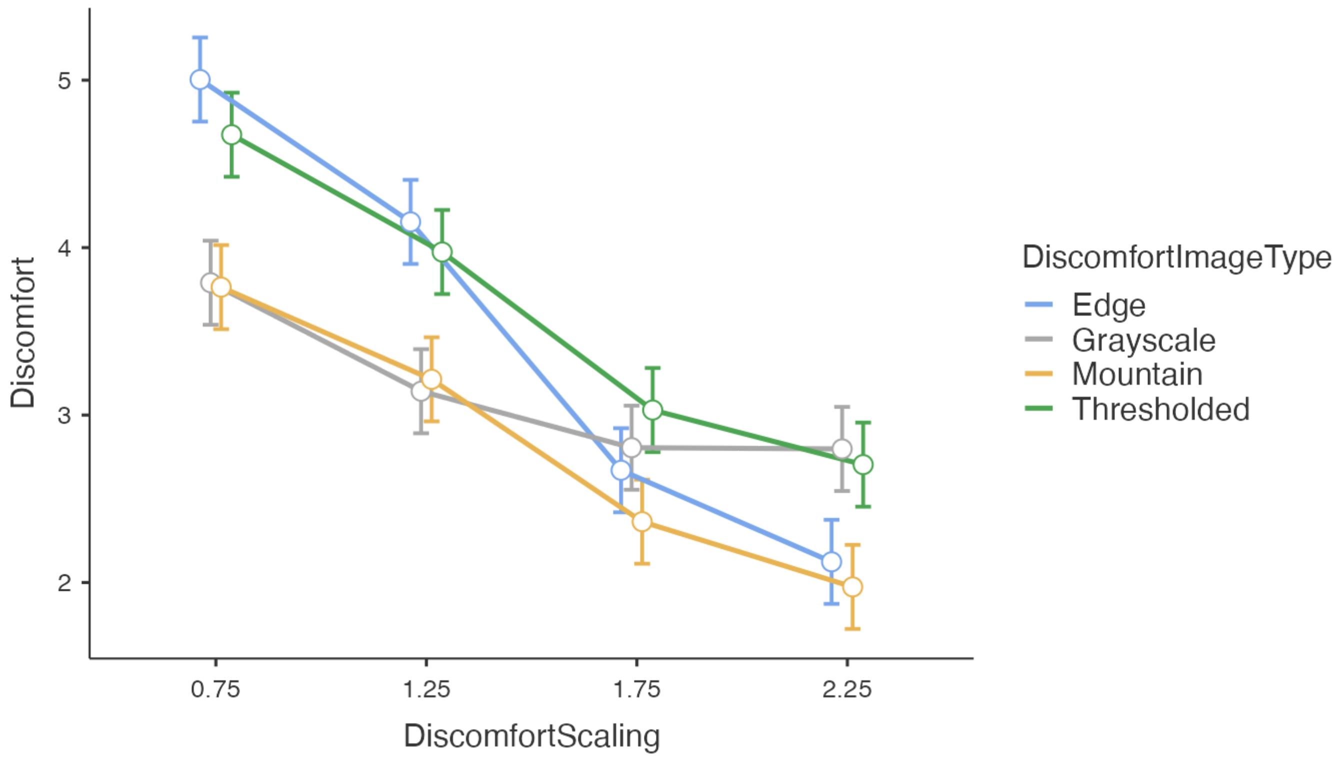

Discomfort
5
4
3
2
0.75
1.25
1.75
2.25
DiscomfortScaling
DiscomfortImageType
Edge
Grayscale
Mountain
Thresholded

Section S5: Discomfort Ratings: LME Model with Trial Number and Repeated Instance Fixed Effects

Model Info

Model Type	Mixed Model	Linear Mixed model for continuous y
Model	lmer	Discomfort ~ 1 + DiscomfortScaling + DiscomfortImageType + TrialNumber + DiscomfortConditionInstance + DiscomfortScaling:DiscomfortImageType + (1 + TrialNumber | ParticipantID) + (1 | DiscomfortImage)
Distribution	Gaussian	Normal distribution of residuals
Direction	y	Dependend variable scores
Optimizer	bobyqa	
DF method	Satterthwaite	
Sample size	4272	
Converged	yes	
Y transform	none	
C.I. method	Wald	

Note. All covariates are in the original scale

Model Results

Model Fit

Type	R^2	df	LRT X^2	p
Conditional	0.448	22	2183.732	<.001
Marginal	0.210	18	208.349	<.001

Note. Model is nearly unidentifiable: very large eigenvalue - Rescale variables?

Fixed Effects Omnibus Tests

	F	df	df (res)	p
DiscomfortScaling	409.289	3	4131.346	<.001
DiscomfortImageType	66.540	3	4129.685	<.001
TrialNumber	5.327	1	186.478	.022
DiscomfortConditionInstance	2.070	2	4093.016	.126
DiscomfortScaling ✻ DiscomfortImageType	19.263	9	4133.649	<.001

Parameter Estimates (Fixed coefficients)

				95% Confidence Intervals				
Names	Effect	Estimate	SE	Lower	Upper	df	t	p
(Intercept)	(Intercept)	4.905	0.137	4.637	5.173	227.669	35.858	<.001
DiscomfortScaling1	1.25 - 0.75	-0.844	0.121	-1.082	-0.606	4125.240	-6.947	<.001
DiscomfortScaling2	1.75 - 0.75	-2.328	0.122	-2.566	-2.089	4130.435	-19.147	<.001
DiscomfortScaling3	2.25 - 0.75	-2.886	0.122	-3.125	-2.648	4132.492	-23.734	<.001
DiscomfortImageType1	Grayscale - Edge	-1.192	0.122	-1.431	-0.954	4129.667	-9.811	<.001
DiscomfortImageType2	Mountain - Edge	-1.222	0.122	-1.460	-0.983	4135.975	-10.042	<.001
DiscomfortImageType3	Thresholded - Edge	-0.286	0.122	-0.524	-0.047	4139.791	-2.345	.019
TrialNumber	TrialNumber	0.007	0.003	0.001	0.013	186.478	2.308	.022
DiscomfortConditionInstance1	2 - 1	-0.118	0.059	-0.234	-0.001	4093.959	-1.981	.048
DiscomfortConditionInstance2	3 - 1	-0.125	0.077	-0.276	0.025	4097.047	-1.636	.102
DiscomfortScaling1 ✻ DiscomfortImageType1	(1.25 - 0.75) ✻ (Grayscale - Edge)	0.184	0.172	-0.154	0.521	4133.794	1.068	.286
DiscomfortScaling2 ✻ DiscomfortImageType1	(1.75 - 0.75) ✻ (Grayscale - Edge)	1.341	0.172	1.003	1.678	4137.682	7.791	<.001
DiscomfortScaling3 ✻ DiscomfortImageType1	(2.25 - 0.75) ✻ (Grayscale - Edge)	1.899	0.172	1.562	2.235	4124.178	11.054	<.001
DiscomfortScaling1 ✻ DiscomfortImageType2	(1.25 - 0.75) ✻ (Mountain - Edge)	0.288	0.172	-0.049	0.625	4125.029	1.677	.094
DiscomfortScaling2 ✻ DiscomfortImageType2	(1.75 - 0.75) ✻ (Mountain - Edge)	0.913	0.172	0.576	1.250	4130.257	5.309	<.001
DiscomfortScaling3 ✻ DiscomfortImageType2	(2.25 - 0.75) ✻ (Mountain - Edge)	1.088	0.172	0.751	1.425	4134.486	6.327	<.001
DiscomfortScaling1 ✻ DiscomfortImageType3	(1.25 - 0.75) ✻ (Thresholded - Edge)	0.103	0.172	-0.235	0.440	4130.096	0.597	.551
DiscomfortScaling2 ✻ DiscomfortImageType3	(1.75 - 0.75) ✻ (Thresholded - Edge)	0.676	0.172	0.338	1.013	4134.131	3.926	<.001
DiscomfortScaling3 ✻ DiscomfortImageType3	(2.25 - 0.75) ✻ (Thresholded - Edge)	0.884	0.172	0.547	1.221	4133.497	5.138	<.001

Random Components

Groups	Name	Variance	SD	ICC
ParticipantID	(Intercept)	0.861	0.928	0.306
	TrialNumber	0.000	0.018	
DiscomfortImage	(Intercept)	0.000	0.000	0.000
Residual		1.950	1.396	

Note. Number of Obs: 4272 , Number of groups: ParticipantID 89, DiscomfortImage 48

Random Parameters correlations

Groups	param1	param2	Cov.	Corr.
ParticipantID	(Intercept)	TrialNumber	-0.006	-0.338

Estimated Marginal Means

Estimate Marginal Means - DiscomfortScaling ✻ DiscomfortImageType

					95% Confidence Intervals	
DiscomfortScaling	DiscomfortImageType	Mean	SE	df	Lower	Upper
0.75	Edge	4.990	0.127	267.747	4.740	5.240
0.75	Grayscale	3.798	0.127	267.303	3.548	4.048
0.75	Mountain	3.768	0.127	267.343	3.518	4.018
0.75	Thresholded	4.704	0.127	268.788	4.454	4.955
1.25	Edge	4.146	0.127	267.402	3.896	4.396
1.25	Grayscale	3.137	0.127	268.360	2.887	3.387
1.25	Mountain	3.212	0.127	267.569	2.962	3.462
1.25	Thresholded	3.963	0.127	267.404	3.713	4.213
1.75	Edge	2.662	0.127	267.076	2.412	2.912
1.75	Grayscale	2.811	0.127	267.717	2.561	3.061
1.75	Mountain	2.353	0.127	267.184	2.103	2.603
1.75	Thresholded	3.052	0.127	267.675	2.802	3.302
2.25	Edge	2.104	0.127	267.630	1.854	2.354
2.25	Grayscale	2.810	0.127	267.483	2.560	3.060
2.25	Mountain	1.970	0.127	267.291	1.720	2.220
2.25	Thresholded	2.702	0.127	267.091	2.452	2.952

Results Plots

Plot: Discomfort ~ TrialNumber ✻ DiscomfortScaling

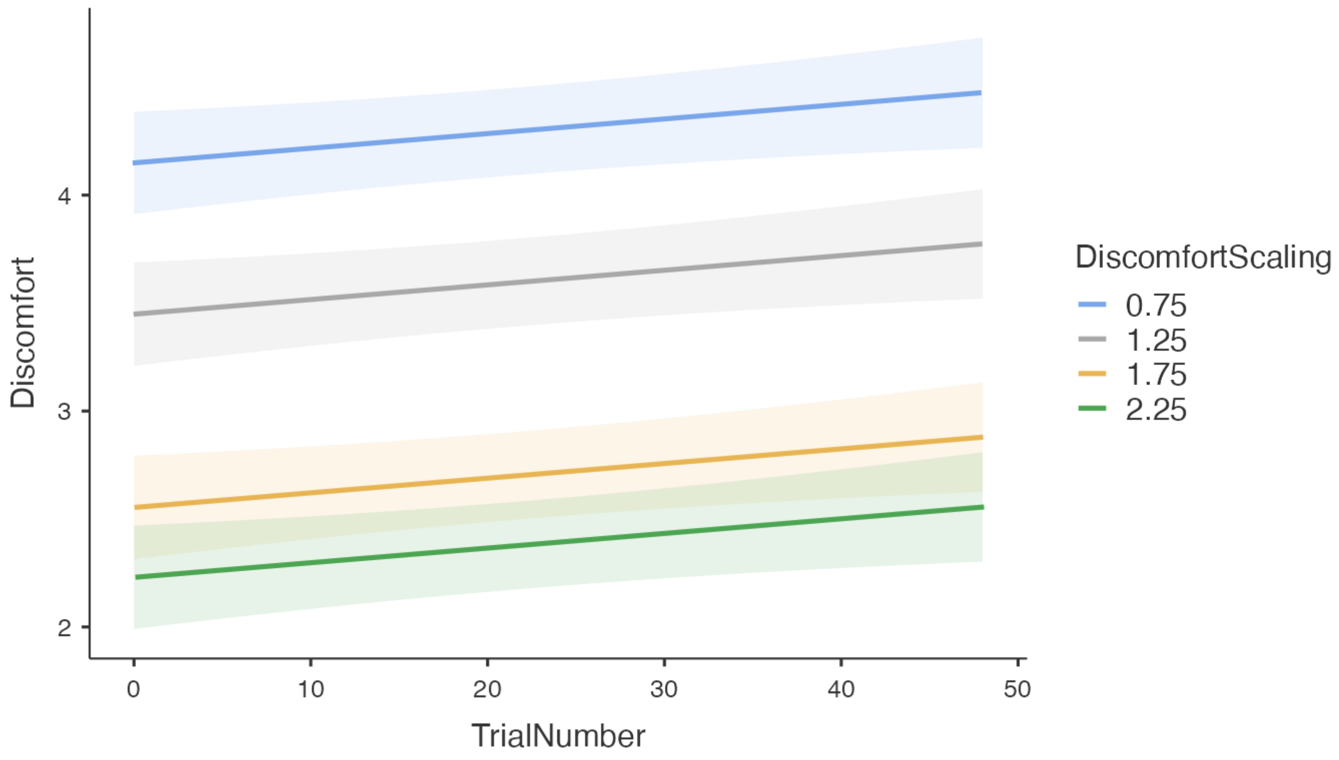

Discomfort
4
3
2
0
10
20
30
40
50
TrialNumber
DiscomfortScaling
0.75
1.25
1.75
2.25

Section S6: Discomfort Ratings: LME Model with Covariates

Model Info

Model Type	Mixed Model	Linear Mixed model for continuous y
Model	lmer	Discomfort ~ 1 + DiscomfortScaling + DiscomfortImageType + SPQ + CATI + CHYPS + ASRS + DiscomfortScaling:DiscomfortImageType + DiscomfortScaling:SPQ + DiscomfortScaling:CATI + DiscomfortScaling:CHYPS + DiscomfortScaling:ASRS + (1 | ParticipantID) + (1 | DiscomfortImage)
Distribution	Gaussian	Normal distribution of residuals
Direction	y	Dependend variable scores
Optimizer	bobyqa	
DF method	Satterthwaite	
Sample size	4272	
Converged	yes	
Y transform	none	
C.I. method	Wald	

Note. All covariates are standardized

Model Results

Model Fit

Type	R^2	df	LRT X^2	p
Conditional	0.440	33	2178.718	<.001
Marginal	0.230	31	252.931	<.001

Fixed Effects Omnibus Tests

	F	df	df (res)	p
DiscomfortScaling	403.331	3	4156.000	<.001
DiscomfortImageType	65.305	3	4156.000	<.001
SPQ	0.634	1	84.000	.428
CATI	1.728	1	84.000	.192
CHYPS	4.347	1	84.000	.040
ASRS	1.736	1	84.000	.191
DiscomfortScaling ✻ DiscomfortImageType	18.734	9	4156.000	<.001
DiscomfortScaling ✻ SPQ	4.156	3	4156.000	.006
DiscomfortScaling ✻ CATI	8.840	3	4156.000	<.001
DiscomfortScaling ✻ CHYPS	4.261	3	4156.000	.005
DiscomfortScaling ✻ ASRS	6.900	3	4156.000	<.001

Parameter Estimates (Fixed coefficients)

Names	Effect	Estimate	SE	95% Confidence Intervals Lower	Upper	df	t	p
(Intercept)	(Intercept)	5.004	0.126	4.756	5.251	265.254	39.666	<.001
DiscomfortScaling1	1.25 - 0.75	-0.850	0.122	-1.090	-0.610	4156.000	-6.950	<.001
DiscomfortScaling2	1.75 - 0.75	-2.333	0.122	-2.573	-2.094	4156.000	-19.075	<.001
DiscomfortScaling3	2.25 - 0.75	-2.880	0.122	-3.120	-2.640	4156.000	-23.546	<.001
DiscomfortImageType1	Grayscale - Edge	-1.213	0.122	-1.453	-0.974	4156.000	-9.920	<.001
DiscomfortImageType2	Mountain - Edge	-1.240	0.122	-1.480	-1.000	4156.000	-10.135	<.001
DiscomfortImageType3	Thresholded - Edge	-0.330	0.122	-0.569	-0.090	4156.000	-2.694	.007
SPQ	SPQ	-0.061	0.118	-0.292	0.171	112.512	-0.513	.609
CATI	CATI	-0.018	0.132	-0.278	0.242	112.512	-0.136	.892
CHYPS	CHYPS	0.118	0.133	-0.144	0.379	112.512	0.882	.379
ASRS	ASRS	-0.014	0.124	-0.258	0.230	112.512	-0.110	.912
DiscomfortScaling1 ✻ DiscomfortImageType1	(1.25 - 0.75) ✻ (Grayscale - Edge)	0.202	0.173	-0.137	0.541	4156.000	1.169	.242
DiscomfortScaling2 ✻ DiscomfortImageType1	(1.75 - 0.75) ✻ (Grayscale - Edge)	1.348	0.173	1.009	1.687	4156.000	7.794	<.001
DiscomfortScaling3 ✻ DiscomfortImageType1	(2.25 - 0.75) ✻ (Grayscale - Edge)	1.888	0.173	1.548	2.227	4156.000	10.912	<.001
DiscomfortScaling1 ✻ DiscomfortImageType2	(1.25 - 0.75) ✻ (Mountain - Edge)	0.300	0.173	-0.040	0.639	4156.000	1.732	.083
DiscomfortScaling2 ✻ DiscomfortImageType2	(1.75 - 0.75) ✻ (Mountain - Edge)	0.933	0.173	0.593	1.272	4156.000	5.391	<.001
DiscomfortScaling3 ✻ DiscomfortImageType2	(2.25 - 0.75) ✻ (Mountain - Edge)	1.090	0.173	0.751	1.429	4156.000	6.300	<.001
DiscomfortScaling1 ✻ DiscomfortImageType3	(1.25 - 0.75) ✻ (Thresholded - Edge)	0.150	0.173	-0.189	0.489	4156.000	0.866	.387
DiscomfortScaling2 ✻ DiscomfortImageType3	(1.75 - 0.75) ✻ (Thresholded - Edge)	0.689	0.173	0.350	1.028	4156.000	3.984	<.001
DiscomfortScaling3 ✻ DiscomfortImageType3	(2.25 - 0.75) ✻ (Thresholded - Edge)	0.910	0.173	0.571	1.249	4156.000	5.261	<.001
DiscomfortScaling1 ✻ SPQ	(1.25 - 0.75) ✻ SPQ	0.160	0.071	0.021	0.300	4156.000	2.252	.024
DiscomfortScaling2 ✻ SPQ	(1.75 - 0.75) ✻ SPQ	0.204	0.071	0.064	0.343	4156.000	2.860	.004
DiscomfortScaling3 ✻ SPQ	(2.25 - 0.75) ✻ SPQ	0.228	0.071	0.089	0.368	4156.000	3.207	.001
DiscomfortScaling1 ✻ CATI	(1.25 - 0.75) ✻ CATI	0.003	0.080	-0.154	0.159	4156.000	0.034	.973

Parameter Estimates (Fixed coefficients)

DiscomfortScaling2 ✻ CATI	(1.75 - 0.75) ✻ CATI	-0.280	0.080	-0.437	-0.124	4156.000	-3.511	<.001
DiscomfortScaling3 ✻ CATI	(2.25 - 0.75) ✻ CATI	-0.298	0.080	-0.454	-0.141	4156.000	-3.731	<.001
DiscomfortScaling1 ✻ CHYPS	(1.25 - 0.75) ✻ CHYPS	0.092	0.080	-0.065	0.249	4156.000	1.147	.251
DiscomfortScaling2 ✻ CHYPS	(1.75 - 0.75) ✻ CHYPS	0.259	0.080	0.102	0.417	4156.000	3.232	.001
DiscomfortScaling3 ✻ CHYPS	(2.25 - 0.75) ✻ CHYPS	0.211	0.080	0.053	0.368	4156.000	2.626	.009
DiscomfortScaling1 ✻ ASRS	(1.25 - 0.75) ✻ ASRS	0.104	0.075	-0.043	0.251	4156.000	1.390	.165
DiscomfortScaling2 ✻ ASRS	(1.75 - 0.75) ✻ ASRS	0.260	0.075	0.113	0.407	4156.000	3.472	<.001
DiscomfortScaling3 ✻ ASRS	(2.25 - 0.75) ✻ ASRS	0.300	0.075	0.153	0.447	4156.000	3.999	<.001

Random Components

Groups	Name	Variance	SD	ICC
ParticipantID	(Intercept)	0.750	0.866	0.273
DiscomfortImage	(Intercept)	0.000	0.000	0.000
Residual		1.998	1.413	

Note. Number of Obs: 4272 , Number of groups: ParticipantID 89, DiscomfortImage 48

Estimated Marginal Means

Estimate Marginal Means - DiscomfortScaling ✻ CATI

					95% Confidence Intervals	
DiscomfortScaling	CATI	Mean	SE	df	Lower	Upper
0.75	Mean-1·SD	4.326	0.167	112.512	3.995	4.657
0.75	Mean	4.308	0.101	112.512	4.107	4.509
0.75	Mean+1·SD	4.290	0.167	112.512	3.959	4.621
1.25	Mean-1·SD	3.636	0.167	112.512	3.305	3.967
1.25	Mean	3.621	0.101	112.512	3.420	3.822
1.25	Mean+1·SD	3.605	0.167	112.512	3.275	3.936
1.75	Mean-1·SD	3.015	0.167	112.512	2.685	3.346
1.75	Mean	2.717	0.101	112.512	2.516	2.918
1.75	Mean+1·SD	2.419	0.167	112.512	2.088	2.750
2.25	Mean-1·SD	2.716	0.167	112.512	2.385	3.046
2.25	Mean	2.400	0.101	112.512	2.199	2.601
2.25	Mean+1·SD	2.084	0.167	112.512	1.753	2.415

Estimate Marginal Means - DiscomfortScaling ✻ CHYPS

					95% Confidence Intervals	
DiscomfortScaling	CHYPS	Mean	SE	df	Lower	Upper
0.75	Mean-1·SD	4.191	0.167	112.512	3.859	4.522
0.75	Mean	4.308	0.101	112.512	4.107	4.509
0.75	Mean+1·SD	4.426	0.167	112.512	4.094	4.757
1.25	Mean-1·SD	3.411	0.167	112.512	3.079	3.743
1.25	Mean	3.621	0.101	112.512	3.420	3.822
1.25	Mean+1·SD	3.830	0.167	112.512	3.499	4.162
1.75	Mean-1·SD	2.340	0.167	112.512	2.008	2.672
1.75	Mean	2.717	0.101	112.512	2.516	2.918
1.75	Mean+1·SD	3.094	0.167	112.512	2.762	3.426
2.25	Mean-1·SD	2.072	0.167	112.512	1.740	2.403
2.25	Mean	2.400	0.101	112.512	2.199	2.601
2.25	Mean+1·SD	2.728	0.167	112.512	2.396	3.060

Estimate Marginal Means - DiscomfortScaling ✻ ASRS

					95% Confidence Intervals	
DiscomfortScaling	ASRS	Mean	SE	df	Lower	Upper
0.75	Mean-1·SD	4.322	0.161	112.512	4.004	4.640
0.75	Mean	4.308	0.101	112.512	4.107	4.509
0.75	Mean+1·SD	4.294	0.161	112.512	3.976	4.612
1.25	Mean-1·SD	3.530	0.161	112.512	3.212	3.848
1.25	Mean	3.621	0.101	112.512	3.420	3.822
1.25	Mean+1·SD	3.711	0.161	112.512	3.393	4.029
1.75	Mean-1·SD	2.471	0.161	112.512	2.152	2.789
1.75	Mean	2.717	0.101	112.512	2.516	2.918
1.75	Mean+1·SD	2.964	0.161	112.512	2.646	3.282
2.25	Mean-1·SD	2.114	0.161	112.512	1.796	2.432
2.25	Mean	2.400	0.101	112.512	2.199	2.601
2.25	Mean+1·SD	2.686	0.161	112.512	2.368	3.004

Estimate Marginal Means - DiscomfortScaling ✻ SPQ

					95% Confidence Intervals	
DiscomfortScaling	SPQ	Mean	SE	df	Lower	Upper
0.75	Mean-1·SD	4.369	0.156	112.512	4.060	4.677
0.75	Mean	4.308	0.101	112.512	4.107	4.509
0.75	Mean+1·SD	4.247	0.156	112.512	3.939	4.556
1.25	Mean-1·SD	3.521	0.156	112.512	3.212	3.830
1.25	Mean	3.621	0.101	112.512	3.420	3.822
1.25	Mean+1·SD	3.721	0.156	112.512	3.412	4.029
1.75	Mean-1·SD	2.574	0.156	112.512	2.265	2.883
1.75	Mean	2.717	0.101	112.512	2.516	2.918
1.75	Mean+1·SD	2.860	0.156	112.512	2.552	3.169
2.25	Mean-1·SD	2.232	0.156	112.512	1.923	2.541
2.25	Mean	2.400	0.101	112.512	2.199	2.601
2.25	Mean+1·SD	2.568	0.156	112.512	2.259	2.876

Results Plots

Plot: Discomfort ~ SPQ ✻ DiscomfortScaling

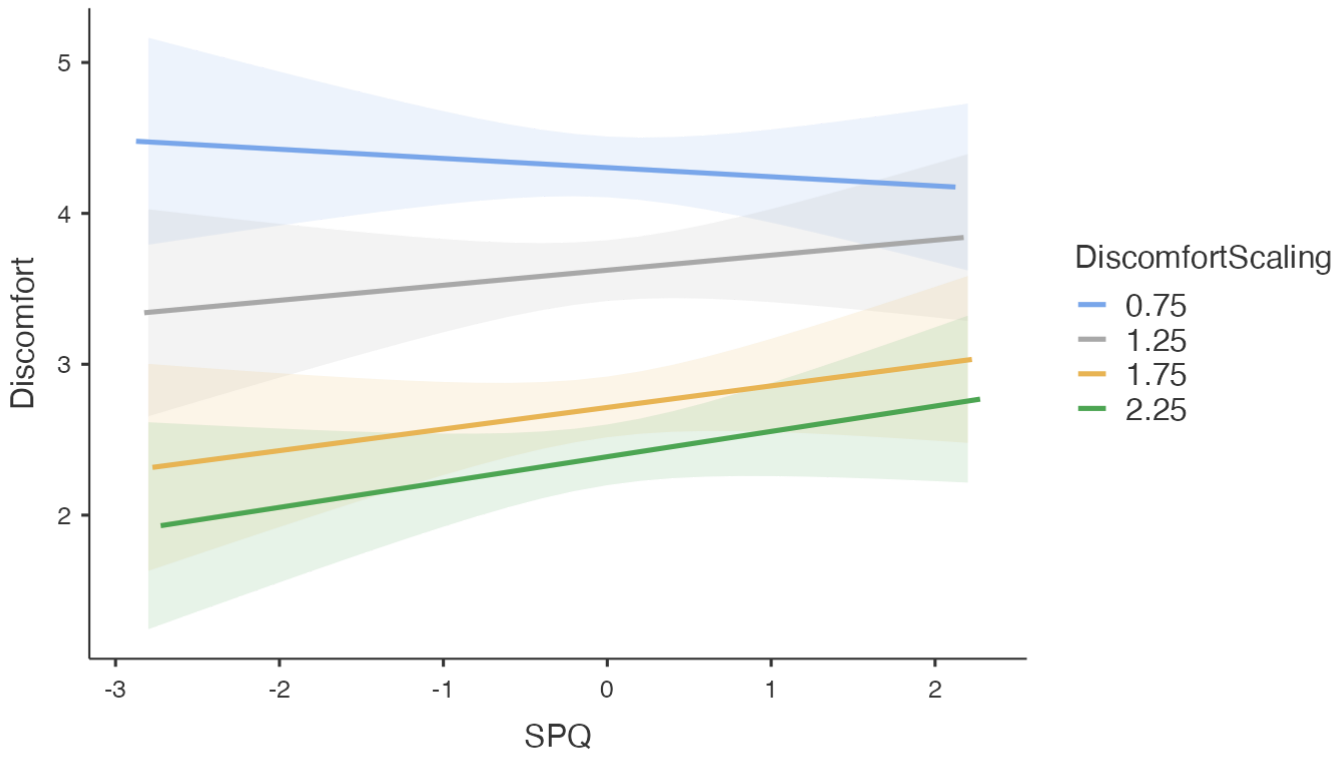

Plot: Discomfort ~ CATI ✻ DiscomfortScaling

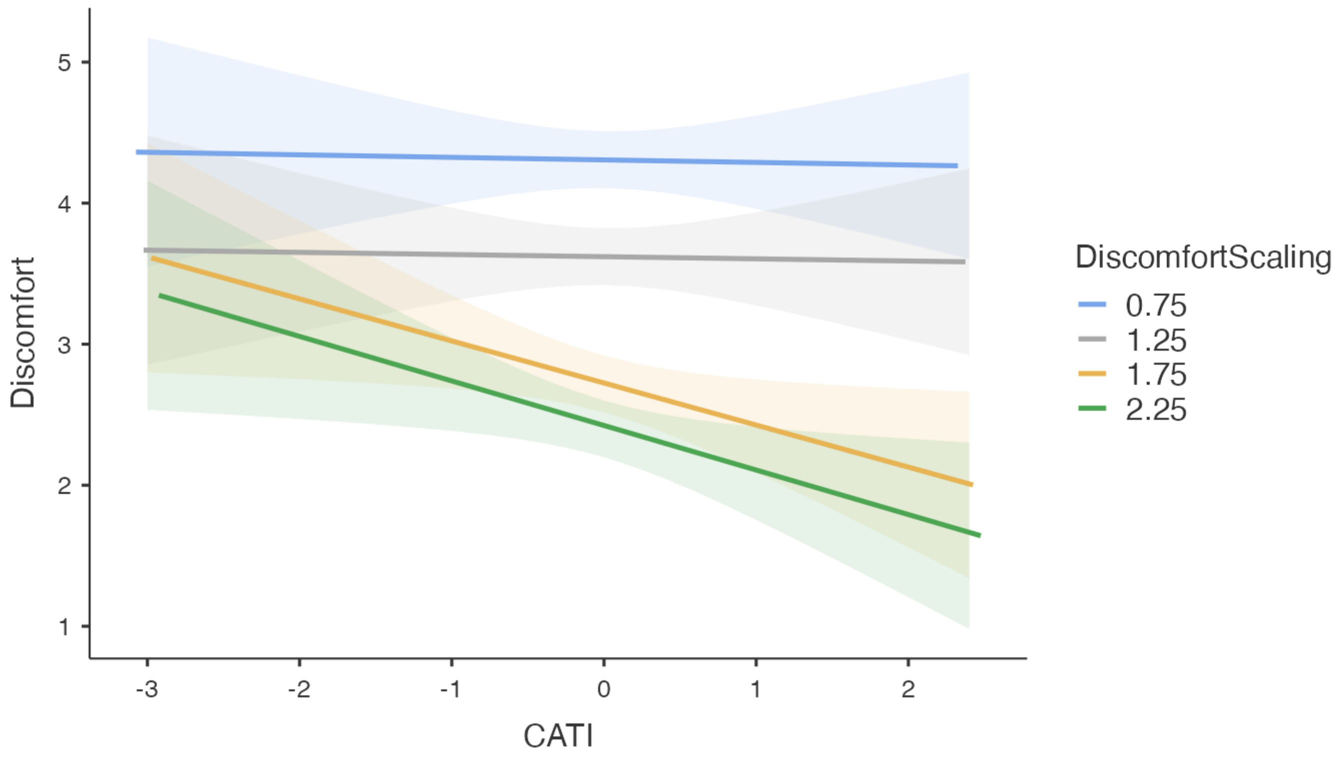

Plot: Discomfort ~ ASRS ∗ DiscomfortScaling

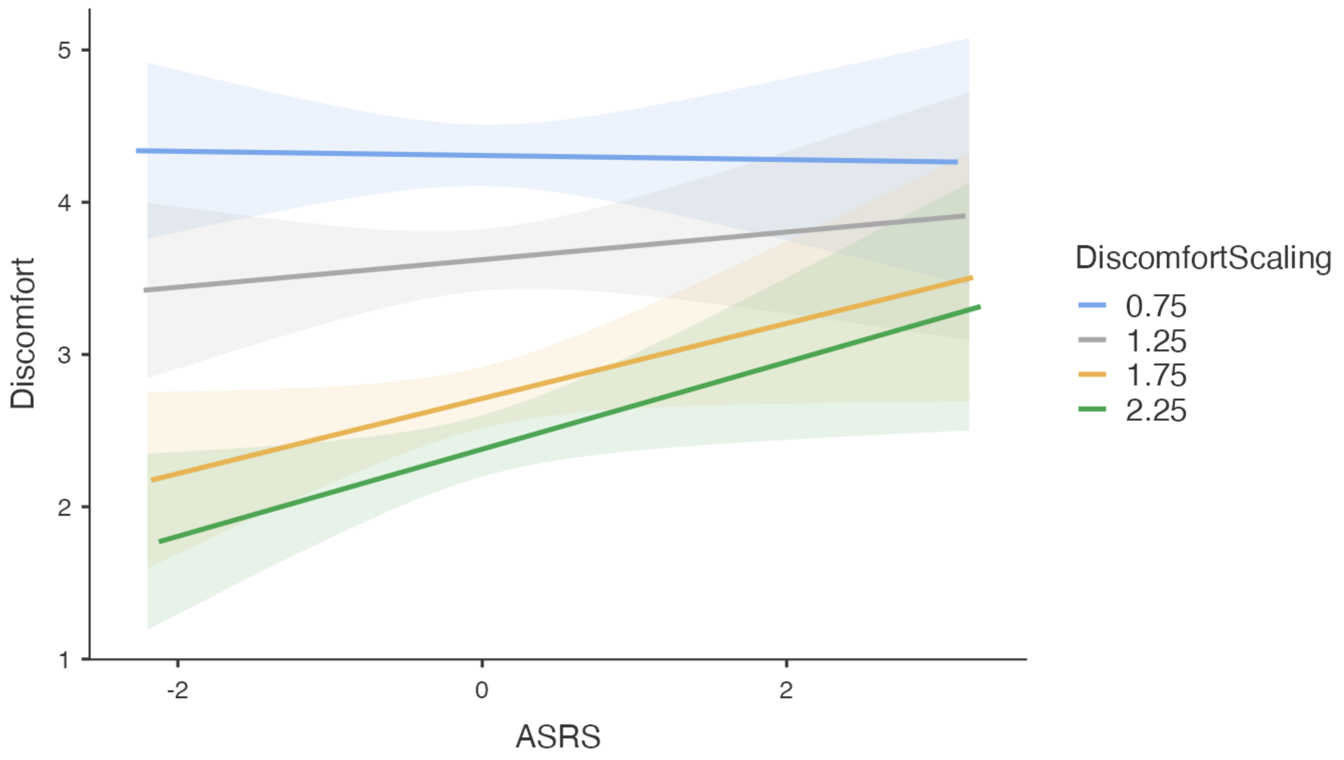

Plot: Discomfort ~ CHYPS ∗ DiscomfortScaling

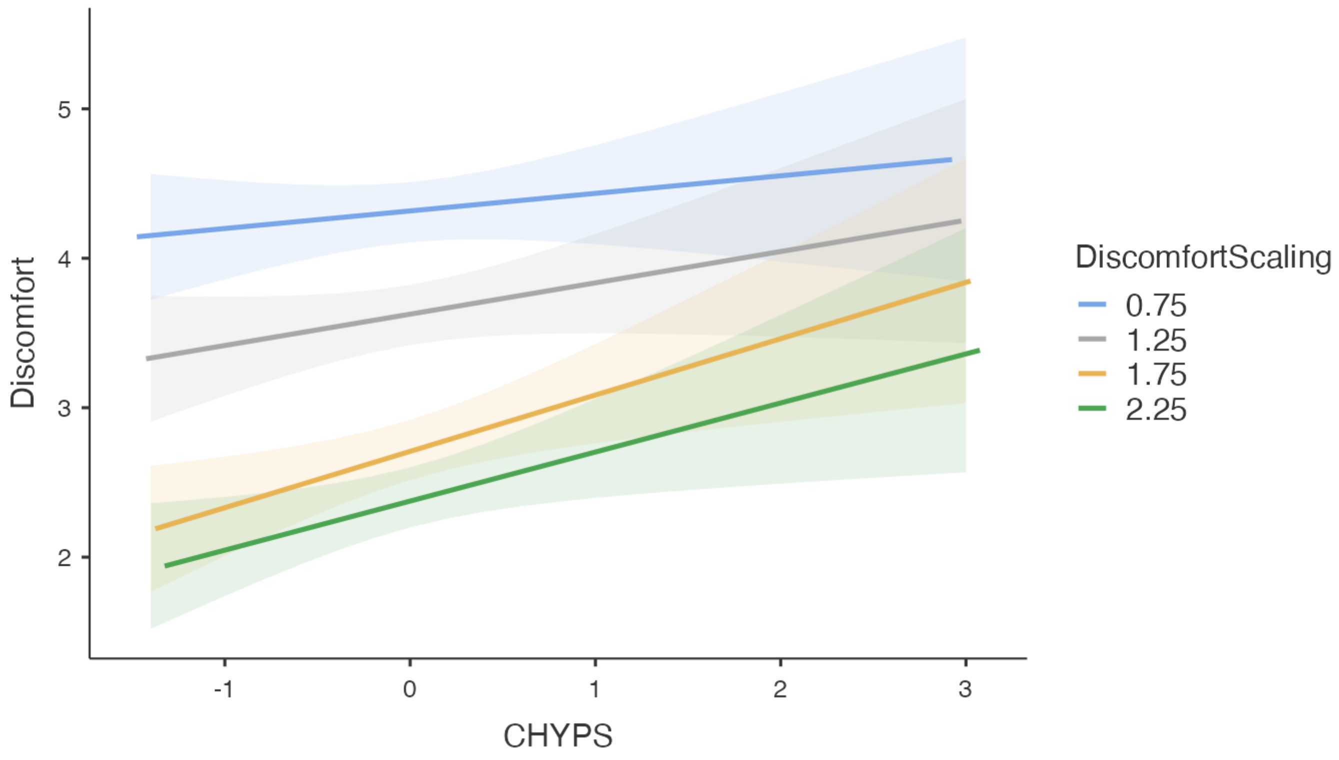
Discomfort
5
4
3
2
-1
0
1
2
3
CHYPS
DiscomfortScaling
0.75
1.25
1.75
2.25

Section S7: Preference Ratings: Initial LME Model

Info		
Model Type	Mixed Model	Linear Mixed model for continuous y
Model	lmer	Preference ~ 1 + PreferenceScaling + PreferenceImageType + PreferenceScaling:PreferenceImageType + (1 l ParticipantID) + (1 l PreferenceImage)
Distribution	Gaussian	Normal distribution of residuals
Direction	y	Dependend variable scores
Optimizer	bobyqa	
DF method	Satterthwaite	
Sample size	4272	
Converged	yes	
Y transform	none	
C.I. method	Wald	

Model Results

Model Fit

Type	R^2	df	LRT X^2	p
Conditional	0.294	17	1224.126	<.001
Marginal	0.046	15	115.059	<.001

Fixed Effects Omnibus Tests

	F	df	df (res)	p
PreferenceScaling	32.432	3	4168.000	<.001
PreferenceImageType	50.529	3	4168.000	<.001
PreferenceScaling ✻ PreferenceImageType	3.291	9	4168.000	<.001

Parameter Estimates (Fixed coefficients)

Names	Effect	Estimate	SE	95% Confidence Intervals Lower	Upper	df	t	p
(Intercept)	(Intercept)	2.730	0.127	2.480	2.980	293.362	21.425	<.001
PreferenceScaling1	1.25 - 0.75	0.150	0.126	-0.097	0.396	4168.000	1.191	.234
PreferenceScaling2	1.75 - 0.75	0.513	0.126	0.267	0.760	4168.000	4.081	<.001
PreferenceScaling3	2.25 - 0.75	0.483	0.126	0.237	0.730	4168.000	3.842	<.001
PreferenceImageType1	Grayscale - Edge	0.558	0.126	0.312	0.805	4168.000	4.438	<.001
PreferenceImageType2	Mountain - Edge	0.120	0.126	-0.127	0.366	4168.000	0.953	.341
PreferenceImageType3	Thresholded - Edge	0.195	0.126	-0.052	0.441	4168.000	1.549	.121
PreferenceScaling1 ✻ PreferenceImageType1	(1.25 - 0.75) ✻ (Grayscale - Edge)	0.326	0.178	-0.023	0.674	4168.000	1.832	.067
PreferenceScaling2 ✻ PreferenceImageType1	(1.75 - 0.75) ✻ (Grayscale - Edge)	0.180	0.178	-0.169	0.528	4168.000	1.011	.312
PreferenceScaling3 ✻ PreferenceImageType1	(2.25 - 0.75) ✻ (Grayscale - Edge)	0.142	0.178	-0.206	0.491	4168.000	0.800	.424
PreferenceScaling1 ✻ PreferenceImageType2	(1.25 - 0.75) ✻ (Mountain - Edge)	-0.045	0.178	-0.394	0.304	4168.000	-0.253	.800
PreferenceScaling2 ✻ PreferenceImageType2	(1.75 - 0.75) ✻ (Mountain - Edge)	0.135	0.178	-0.214	0.483	4168.000	0.758	.448
PreferenceScaling3 ✻ PreferenceImageType2	(2.25 - 0.75) ✻ (Mountain - Edge)	0.288	0.178	-0.060	0.637	4168.000	1.622	.105
PreferenceScaling1 ✻ PreferenceImageType3	(1.25 - 0.75) ✻ (Thresholded - Edge)	0.210	0.178	-0.139	0.558	4168.000	1.179	.238
PreferenceScaling2 ✻ PreferenceImageType3	(1.75 - 0.75) ✻ (Thresholded - Edge)	-0.213	0.178	-0.562	0.135	4168.000	-1.201	.230
PreferenceScaling3 ✻ PreferenceImageType3	(2.25 - 0.75) ✻ (Thresholded - Edge)	-0.277	0.178	-0.626	0.071	4168.000	-1.559	.119

Random Components

Groups	Name	Variance	SD	ICC
ParticipantID	(Intercept)	0.742	0.861	0.260
PreferenceImage	(Intercept)	0.000	0.000	0.000
Residual		2.111	1.453	

Note. Number of Obs: 4272 , Number of groups: ParticipantID 89, PreferenceImage 48

Post Hoc Tests

Post Hoc comparison: PreferenceScaling

Comparison PreferenceScaling	vs	PreferenceScaling	Difference	SE	t	df	$p_{bonferroni}$	p_{holm}
0.75	-	1.25	-0.272	0.063	-4.334	4168.000	<.001	<.001
0.75	-	1.75	-0.538	0.063	-8.564	4168.000	<.001	<.001
0.75	-	2.25	-0.522	0.063	-8.296	4168.000	<.001	<.001
1.25	-	1.75	-0.266	0.063	-4.230	4168.000	<.001	<.001
1.25	-	2.25	-0.249	0.063	-3.962	4168.000	<.001	<.001
1.75	-	2.25	0.017	0.063	0.268	4168.000	1.000	.789

Post Hoc comparison: PreferenceImageType

Comparison								
PreferenceImageType	vs	PreferenceImageType	Difference	SE	t	df	$p_{bonferroni}$	p_{holm}
Edge	-	Grayscale	-0.720	0.063	-11.453	4168.000	<.001	<.001
Edge	-	Mountain	-0.214	0.063	-3.411	4168.000	.004	.002
Edge	-	Thresholded	-0.125	0.063	-1.981	4168.000	.286	.095
Grayscale	-	Mountain	0.506	0.063	8.042	4168.000	<.001	<.001
Grayscale	-	Thresholded	0.596	0.063	9.472	4168.000	<.001	<.001
Mountain	-	Thresholded	0.090	0.063	1.430	4168.000	.917	.153

Post Hoc comparison: PreferenceScaling ✻ PreferenceImageType

Comparison										
PreferenceScaling	PreferenceImageType	vs	PreferenceScaling	PreferenceImageType	Difference	SE	t	df	$p_{bonferroni}$	p_{holm}
0.75	Edge	-	0.75	Grayscale	-0.558	0.126	-4.438	4168.000	.001	<.001
0.75	Edge	-	0.75	Mountain	-0.120	0.126	-0.953	4168.000	1.000	1.000
0.75	Edge	-	0.75	Thresholded	-0.195	0.126	-1.549	4168.000	1.000	1.000
0.75	Edge	-	1.25	Edge	-0.150	0.126	-1.191	4168.000	1.000	1.000
0.75	Edge	-	1.25	Grayscale	-1.034	0.126	-8.221	4168.000	<.001	<.001
0.75	Edge	-	1.25	Mountain	-0.225	0.126	-1.787	4168.000	1.000	1.000
0.75	Edge	-	1.25	Thresholded	-0.554	0.126	-4.408	4168.000	.001	<.001
0.75	Edge	-	1.75	Edge	-0.513	0.126	-4.081	4168.000	.005	.004
0.75	Edge	-	1.75	Grayscale	-1.251	0.126	-9.949	4168.000	<.001	<.001
0.75	Edge	-	1.75	Mountain	-0.768	0.126	-6.106	4168.000	<.001	<.001
0.75	Edge	-	1.75	Thresholded	-0.494	0.126	-3.932	4168.000	.010	.007
0.75	Edge	-	2.25	Edge	-0.483	0.126	-3.842	4168.000	.015	.009
0.75	Edge	-	2.25	Grayscale	-1.184	0.126	-9.413	4168.000	<.001	<.001
0.75	Edge	-	2.25	Mountain	-0.891	0.126	-7.089	4168.000	<.001	<.001
0.75	Edge	-	2.25	Thresholded	-0.401	0.126	-3.187	4168.000	.174	.093
0.75	Grayscale	-	0.75	Mountain	0.438	0.126	3.485	4168.000	.060	.035
0.75	Grayscale	-	0.75	Thresholded	0.363	0.126	2.889	4168.000	.466	.225
0.75	Grayscale	-	1.25	Edge	0.408	0.126	3.247	4168.000	.141	.079
0.75	Grayscale	-	1.25	Grayscale	-0.476	0.126	-3.783	4168.000	.019	.011
0.75	Grayscale	-	1.25	Mountain	0.333	0.126	2.651	4168.000	.967	.411
0.75	Grayscale	-	1.25	Thresholded	0.004	0.126	0.030	4168.000	1.000	1.000
0.75	Grayscale	-	1.75	Edge	0.045	0.126	0.357	4168.000	1.000	1.000
0.75	Grayscale	-	1.75	Grayscale	-0.693	0.126	-5.511	4168.000	<.001	<.001
0.75	Grayscale	-	1.75	Mountain	-0.210	0.126	-1.668	4168.000	1.000	1.000
0.75	Grayscale	-	1.75	Thresholded	0.064	0.126	0.506	4168.000	1.000	1.000
0.75	Grayscale	-	2.25	Edge	0.075	0.126	0.596	4168.000	1.000	1.000
0.75	Grayscale	-	2.25	Grayscale	-0.625	0.126	-4.974	4168.000	<.001	<.001
0.75	Grayscale	-	2.25	Mountain	-0.333	0.126	-2.651	4168.000	.967	.411
0.75	Grayscale	-	2.25	Thresholded	0.157	0.126	1.251	4168.000	1.000	1.000
0.75	Mountain	-	0.75	Thresholded	-0.075	0.126	-0.596	4168.000	1.000	1.000
0.75	Mountain	-	1.25	Edge	-0.030	0.126	-0.238	4168.000	1.000	1.000
0.75	Mountain	-	1.25	Grayscale	-0.914	0.126	-7.268	4168.000	<.001	<.001
0.75	Mountain	-	1.25	Mountain	-0.105	0.126	-0.834	4168.000	1.000	1.000
0.75	Mountain	-	1.25	Thresholded	-0.434	0.126	-3.455	4168.000	.067	.038
0.75	Mountain	-	1.75	Edge	-0.393	0.126	-3.128	4168.000	.213	.110
0.75	Mountain	-	1.75	Grayscale	-1.131	0.126	-8.996	4168.000	<.001	<.001
0.75	Mountain	-	1.75	Mountain	-0.648	0.126	-5.153	4168.000	<.001	<.001
0.75	Mountain	-	1.75	Thresholded	-0.375	0.126	-2.979	4168.000	.349	.175
0.75	Mountain	-	2.25	Edge	-0.363	0.126	-2.889	4168.000	.466	.225
0.75	Mountain	-	2.25	Grayscale	-1.064	0.126	-8.459	4168.000	<.001	<.001
0.75	Mountain	-	2.25	Mountain	-0.772	0.126	-6.136	4168.000	<.001	<.001

Post Hoc comparison: PreferenceScaling ✻ PreferenceImageType

0.75	Mountain	-	2.25	Thresholded	-0.281	0.126	-2.234	4168.000	1.000	1.000
0.75	Thresholded	-	1.25	Edge	0.045	0.126	0.357	4168.000	1.000	1.000
0.75	Thresholded	-	1.25	Grayscale	-0.839	0.126	-6.672	4168.000	<.001	<.001
0.75	Thresholded	-	1.25	Mountain	-0.030	0.126	-0.238	4168.000	1.000	1.000
0.75	Thresholded	-	1.25	Thresholded	-0.360	0.126	-2.860	4168.000	.512	.235
0.75	Thresholded	-	1.75	Edge	-0.318	0.126	-2.532	4168.000	1.000	.535
0.75	Thresholded	-	1.75	Grayscale	-1.056	0.126	-8.400	4168.000	<.001	<.001
0.75	Thresholded	-	1.75	Mountain	-0.573	0.126	-4.557	4168.000	<.001	<.001
0.75	Thresholded	-	1.75	Thresholded	-0.300	0.126	-2.383	4168.000	1.000	.792
0.75	Thresholded	-	2.25	Edge	-0.288	0.126	-2.294	4168.000	1.000	.962
0.75	Thresholded	-	2.25	Grayscale	-0.989	0.126	-7.864	4168.000	<.001	<.001
0.75	Thresholded	-	2.25	Mountain	-0.697	0.126	-5.540	4168.000	<.001	<.001
0.75	Thresholded	-	2.25	Thresholded	-0.206	0.126	-1.638	4168.000	1.000	1.000
1.25	Edge	-	1.25	Grayscale	-0.884	0.126	-7.030	4168.000	<.001	<.001
1.25	Edge	-	1.25	Mountain	-0.075	0.126	-0.596	4168.000	1.000	1.000
1.25	Edge	-	1.25	Thresholded	-0.404	0.126	-3.217	4168.000	.157	.085
1.25	Edge	-	1.75	Edge	-0.363	0.126	-2.889	4168.000	.466	.225
1.25	Edge	-	1.75	Grayscale	-1.101	0.126	-8.757	4168.000	<.001	<.001
1.25	Edge	-	1.75	Mountain	-0.618	0.126	-4.915	4168.000	<.001	<.001
1.25	Edge	-	1.75	Thresholded	-0.345	0.126	-2.740	4168.000	.740	.327
1.25	Edge	-	2.25	Edge	-0.333	0.126	-2.651	4168.000	.967	.411
1.25	Edge	-	2.25	Grayscale	-1.034	0.126	-8.221	4168.000	<.001	<.001
1.25	Edge	-	2.25	Mountain	-0.742	0.126	-5.898	4168.000	<.001	<.001
1.25	Edge	-	2.25	Thresholded	-0.251	0.126	-1.996	4168.000	1.000	1.000
1.25	Grayscale	-	1.25	Mountain	0.809	0.126	6.434	4168.000	<.001	<.001
1.25	Grayscale	-	1.25	Thresholded	0.479	0.126	3.813	4168.000	.017	.010
1.25	Grayscale	-	1.75	Edge	0.521	0.126	4.140	4168.000	.004	.003
1.25	Grayscale	-	1.75	Grayscale	-0.217	0.126	-1.728	4168.000	1.000	1.000
1.25	Grayscale	-	1.75	Mountain	0.266	0.126	2.115	4168.000	1.000	1.000
1.25	Grayscale	-	1.75	Thresholded	0.539	0.126	4.289	4168.000	.002	.001
1.25	Grayscale	-	2.25	Edge	0.551	0.126	4.379	4168.000	.001	<.001
1.25	Grayscale	-	2.25	Grayscale	-0.150	0.126	-1.191	4168.000	1.000	1.000
1.25	Grayscale	-	2.25	Mountain	0.142	0.126	1.132	4168.000	1.000	1.000
1.25	Grayscale	-	2.25	Thresholded	0.633	0.126	5.034	4168.000	<.001	<.001
1.25	Mountain	-	1.25	Thresholded	-0.330	0.126	-2.621	4168.000	1.000	.422
1.25	Mountain	-	1.75	Edge	-0.288	0.126	-2.294	4168.000	1.000	.962
1.25	Mountain	-	1.75	Grayscale	-1.026	0.126	-8.162	4168.000	<.001	<.001
1.25	Mountain	-	1.75	Mountain	-0.543	0.126	-4.319	4168.000	.002	.001
1.25	Mountain	-	1.75	Thresholded	-0.270	0.126	-2.145	4168.000	1.000	1.000
1.25	Mountain	-	2.25	Edge	-0.258	0.126	-2.055	4168.000	1.000	1.000
1.25	Mountain	-	2.25	Grayscale	-0.959	0.126	-7.625	4168.000	<.001	<.001
1.25	Mountain	-	2.25	Mountain	-0.667	0.126	-5.302	4168.000	<.001	<.001
1.25	Mountain	-	2.25	Thresholded	-0.176	0.126	-1.400	4168.000	1.000	1.000
1.25	Thresholded	-	1.75	Edge	0.041	0.126	0.328	4168.000	1.000	1.000
1.25	Thresholded	-	1.75	Grayscale	-0.697	0.126	-5.540	4168.000	<.001	<.001
1.25	Thresholded	-	1.75	Mountain	-0.213	0.126	-1.698	4168.000	1.000	1.000
1.25	Thresholded	-	1.75	Thresholded	0.060	0.126	0.477	4168.000	1.000	1.000
1.25	Thresholded	-	2.25	Edge	0.071	0.126	0.566	4168.000	1.000	1.000
1.25	Thresholded	-	2.25	Grayscale	-0.629	0.126	-5.004	4168.000	<.001	<.001
1.25	Thresholded	-	2.25	Mountain	-0.337	0.126	-2.681	4168.000	.885	.383
1.25	Thresholded	-	2.25	Thresholded	0.154	0.126	1.221	4168.000	1.000	1.000
1.75	Edge	-	1.75	Grayscale	-0.738	0.126	-5.868	4168.000	<.001	<.001
1.75	Edge	-	1.75	Mountain	-0.255	0.126	-2.025	4168.000	1.000	1.000
1.75	Edge	-	1.75	Thresholded	0.019	0.126	0.149	4168.000	1.000	1.000
1.75	Edge	-	2.25	Edge	0.030	0.126	0.238	4168.000	1.000	1.000

Post Hoc comparison: PreferenceScaling ✻ PreferenceImageType

1.75	Edge	-	2.25	Grayscale	-0.670	0.126	-5.332	4168.000	<.001	<.001
1.75	Edge	-	2.25	Mountain	-0.378	0.126	-3.008	4168.000	.317	.161
1.75	Edge	-	2.25	Thresholded	0.112	0.126	0.894	4168.000	1.000	1.000
1.75	Grayscale	-	1.75	Mountain	0.483	0.126	3.842	4168.000	.015	.009
1.75	Grayscale	-	1.75	Thresholded	0.757	0.126	6.017	4168.000	<.001	<.001
1.75	Grayscale	-	2.25	Edge	0.768	0.126	6.106	4168.000	<.001	<.001
1.75	Grayscale	-	2.25	Grayscale	0.067	0.126	0.536	4168.000	1.000	1.000
1.75	Grayscale	-	2.25	Mountain	0.360	0.126	2.860	4168.000	.512	.235
1.75	Grayscale	-	2.25	Thresholded	0.850	0.126	6.762	4168.000	<.001	<.001
1.75	Mountain	-	1.75	Thresholded	0.273	0.126	2.174	4168.000	1.000	1.000
1.75	Mountain	-	2.25	Edge	0.285	0.126	2.264	4168.000	1.000	.993
1.75	Mountain	-	2.25	Grayscale	-0.416	0.126	-3.306	4168.000	.114	.065
1.75	Mountain	-	2.25	Mountain	-0.124	0.126	-0.983	4168.000	1.000	1.000
1.75	Mountain	-	2.25	Thresholded	0.367	0.126	2.919	4168.000	.424	.208
1.75	Thresholded	-	2.25	Edge	0.011	0.126	0.089	4168.000	1.000	1.000
1.75	Thresholded	-	2.25	Grayscale	-0.689	0.126	-5.481	4168.000	<.001	<.001
1.75	Thresholded	-	2.25	Mountain	-0.397	0.126	-3.157	4168.000	.192	.101
1.75	Thresholded	-	2.25	Thresholded	0.094	0.126	0.745	4168.000	1.000	1.000
2.25	Edge	-	2.25	Grayscale	-0.700	0.126	-5.570	4168.000	<.001	<.001
2.25	Edge	-	2.25	Mountain	-0.408	0.126	-3.247	4168.000	.141	.079
2.25	Edge	-	2.25	Thresholded	0.082	0.126	0.655	4168.000	1.000	1.000
2.25	Grayscale	-	2.25	Mountain	0.292	0.126	2.323	4168.000	1.000	.909
2.25	Grayscale	-	2.25	Thresholded	0.783	0.126	6.225	4168.000	<.001	<.001
2.25	Mountain	-	2.25	Thresholded	0.491	0.126	3.902	4168.000	.012	.007

Simple Effects

ANOVA for Simple Effects of PreferenceImageType

Moderator				
PreferenceScaling	F	Num df	Den df	p
0.75	7.309	3.000	4168.000	<.001
1.25	20.487	3.000	4168.000	<.001
1.75	15.709	3.000	4168.000	<.001
2.25	16.896	3.000	4168.000	<.001

Parameter Estimates for simple effects of PreferenceImageType

Moderator				95% Confidence Intervals				
PreferenceScaling	Effect	Estimate	SE	Lower	Upper	df	t	p
0.75	Grayscale - Edge	0.558	0.126	0.312	0.805	4168.000	4.438	<.001
	Mountain - Edge	0.120	0.126	-0.127	0.366	4168.000	0.953	.341
	Thresholded - Edge	0.195	0.126	-0.052	0.441	4168.000	1.549	.121
1.25	Grayscale - Edge	0.884	0.126	0.637	1.130	4168.000	7.030	<.001
	Mountain - Edge	0.075	0.126	-0.172	0.321	4168.000	0.596	.551
	Thresholded - Edge	0.404	0.126	0.158	0.651	4168.000	3.217	.001
1.75	Grayscale - Edge	0.738	0.126	0.491	0.984	4168.000	5.868	<.001
	Mountain - Edge	0.255	0.126	0.008	0.501	4168.000	2.025	.043
	Thresholded - Edge	-0.019	0.126	-0.265	0.228	4168.000	-0.149	.882
2.25	Grayscale - Edge	0.700	0.126	0.454	0.947	4168.000	5.570	<.001
	Mountain - Edge	0.408	0.126	0.162	0.655	4168.000	3.247	.001
	Thresholded - Edge	-0.082	0.126	-0.329	0.164	4168.000	-0.655	.512

Estimated Marginal Means

Estimate Marginal Means - PreferenceScaling ✻ PreferenceImageType

					95% Confidence Intervals	
PreferenceScaling	PreferenceImageType	Mean	SE	df	Lower	Upper
0.75	Edge	2.730	0.127	293.362	2.480	2.981
0.75	Grayscale	3.288	0.127	293.362	3.038	3.539
0.75	Mountain	2.850	0.127	293.362	2.599	3.101
0.75	Thresholded	2.925	0.127	293.362	2.674	3.176
1.25	Edge	2.880	0.127	293.362	2.629	3.131
1.25	Grayscale	3.764	0.127	293.362	3.513	4.015
1.25	Mountain	2.955	0.127	293.362	2.704	3.206
1.25	Thresholded	3.285	0.127	293.362	3.034	3.535
1.75	Edge	3.243	0.127	293.362	2.993	3.494
1.75	Grayscale	3.981	0.127	293.362	3.730	4.232
1.75	Mountain	3.498	0.127	293.362	3.247	3.749
1.75	Thresholded	3.225	0.127	293.362	2.974	3.476
2.25	Edge	3.213	0.127	293.362	2.963	3.464
2.25	Grayscale	3.914	0.127	293.362	3.663	4.165
2.25	Mountain	3.622	0.127	293.362	3.371	3.873
2.25	Thresholded	3.131	0.127	293.362	2.880	3.382

Estimate Marginal Means - PreferenceScaling

				95% Confidence Intervals	
PreferenceScaling	Mean	SE	df	Lower	Upper
0.75	2.949	0.102	119.954	2.747	3.150
1.25	3.221	0.102	119.954	3.020	3.422
1.75	3.487	0.102	119.954	3.286	3.688
2.25	3.470	0.102	119.954	3.269	3.671

Estimate Marginal Means - PreferenceImageType

				95% Confidence Intervals	
PreferenceImageType	Mean	SE	df	Lower	Upper
Edge	3.017	0.102	119.954	2.816	3.218
Grayscale	3.737	0.102	119.954	3.536	3.938
Mountain	3.231	0.102	119.954	3.030	3.432
Thresholded	3.141	0.102	119.954	2.940	3.342

Section S8: Preference Ratings: LME Model with Trial Number and Repeated Instance Fixed Effects

Model Info

Model Type	Mixed Model	Linear Mixed model for continuous y
Model	lmer	Preference ~ 1 + PreferenceScaling + PreferenceImageType + TrialNumber + PreferenceConditionInstance + PreferenceScaling:PreferenceImageType + (1 + TrialNumber | ParticipantID) + (1 | PreferenceImage)
Distribution	Gaussian	Normal distribution of residuals
Direction	y	Dependend variable scores
Optimizer	bobyqa	
DF method	Satterthwaite	
Sample size	4272	
Converged	yes	
Y transform	none	
C.I. method	Wald	

Note. All covariates are centered to the mean

Model Results

Model Fit

Type	R^2	df	LRT X^2	p
Conditional	0.305	22	1244.905	<.001
Marginal	0.047	18	119.397	<.001

Fixed Effects Omnibus Tests

	F	df	df (res)	p
PreferenceScaling	32.500	3	4147.913	<.001
PreferenceImageType	50.861	3	4153.924	<.001
TrialNumber	0.170	1	233.758	.680
PreferenceConditionInstance	1.582	2	4099.653	.206
PreferenceScaling ✻ PreferenceImageType	3.354	9	4147.591	<.001

Parameter Estimates (Fixed coefficients)

				95% Confidence Intervals				
Names	Effect	Estimate	SE	Lower	Upper	df	t	p
(Intercept)	(Intercept)	2.737	0.127	2.488	2.987	292.290	21.501	<.001
PreferenceScaling1	1.25 - 0.75	0.135	0.125	-0.111	0.381	4153.182	1.076	.282
PreferenceScaling2	1.75 - 0.75	0.502	0.125	0.257	0.748	4146.664	4.008	<.001
PreferenceScaling3	2.25 - 0.75	0.476	0.125	0.230	0.722	4146.537	3.796	<.001
PreferenceImageType1	Grayscale - Edge	0.547	0.125	0.301	0.792	4152.645	4.358	<.001
PreferenceImageType2	Mountain - Edge	0.107	0.126	-0.139	0.353	4160.454	0.855	.392
PreferenceImageType3	Thresholded - Edge	0.197	0.125	-0.049	0.443	4148.770	1.571	.116
TrialNumber	TrialNumber	0.001	0.003	-0.004	0.006	233.758	0.412	.680
PreferenceConditionInstance1	2 - 1	-0.016	0.061	-0.135	0.104	4095.407	-0.256	.798
PreferenceConditionInstance2	3 - 1	-0.119	0.078	-0.272	0.035	4105.776	-1.515	.130
PreferenceScaling1 ✻ PreferenceImageType1	(1.25 - 0.75) ✻ (Grayscale - Edge)	0.350	0.177	0.003	0.698	4153.636	1.976	.048
PreferenceScaling2 ✻ PreferenceImageType1	(1.75 - 0.75) ✻ (Grayscale - Edge)	0.197	0.177	-0.151	0.544	4144.665	1.111	.267
PreferenceScaling3 ✻ PreferenceImageType1	(2.25 - 0.75) ✻ (Grayscale - Edge)	0.156	0.177	-0.192	0.503	4149.703	0.879	.380
PreferenceScaling1 ✻ PreferenceImageType2	(1.25 - 0.75) ✻ (Mountain - Edge)	-0.030	0.177	-0.378	0.318	4155.963	-0.171	.864
PreferenceScaling2 ✻ PreferenceImageType2	(1.75 - 0.75) ✻ (Mountain - Edge)	0.150	0.177	-0.198	0.497	4154.885	0.843	.399
PreferenceScaling3 ✻ PreferenceImageType2	(2.25 - 0.75) ✻ (Mountain - Edge)	0.293	0.177	-0.055	0.640	4151.627	1.651	.099
PreferenceScaling1 ✻ PreferenceImageType3	(1.25 - 0.75) ✻ (Thresholded - Edge)	0.219	0.177	-0.129	0.566	4148.171	1.233	.218
PreferenceScaling2 ✻ PreferenceImageType3	(1.75 - 0.75) ✻ (Thresholded - Edge)	-0.209	0.177	-0.557	0.139	4147.895	-1.177	.239
PreferenceScaling3 ✻ PreferenceImageType3	(2.25 - 0.75) ✻ (Thresholded - Edge)	-0.272	0.177	-0.619	0.076	4152.392	-1.530	.126

Random Components

Groups	Name	Variance	SD	ICC
ParticipantID	(Intercept)	0.742	0.862	0.263
	TrialNumber	0.000	0.013	
PreferenceImage	(Intercept)	0.000	0.000	0.000
Residual		2.078	1.442	

Note. Number of Obs: 4272 , Number of groups: ParticipantID 89, PreferenceImage 48

Random Parameters correlations

Groups	param1	param2	Cov.	Corr.
ParticipantID	(Intercept)	TrialNumber	0.002	0.223

Simple Effects

ANOVA for Simple Effects of PreferenceImageType

Moderator				
PreferenceScaling	F	Num df	Den df	p
0.75	7.128	3.000	4148.770	<.001
1.25	21.140	3.000	4143.170	<.001
1.75	15.915	3.000	4139.100	<.001
2.25	16.754	3.000	4146.070	<.001

Parameter Estimates for simple effects of PreferenceImageType

Moderator				95% Confidence Intervals				
PreferenceScaling	Effect	Estimate	SE	Lower	Upper	df	t	p
0.75	Grayscale - Edge	0.547	0.125	0.301	0.792	4152.645	4.358	<.001
	Mountain - Edge	0.107	0.126	-0.139	0.353	4160.454	0.855	.392
	Thresholded - Edge	0.197	0.125	-0.049	0.443	4148.770	1.571	.116
1.25	Grayscale - Edge	0.897	0.125	0.651	1.143	4154.856	7.148	<.001
	Mountain - Edge	0.077	0.125	-0.169	0.323	4150.065	0.614	.539
	Thresholded - Edge	0.416	0.125	0.170	0.661	4143.171	3.320	<.001
1.75	Grayscale - Edge	0.743	0.125	0.498	0.989	4139.102	5.937	<.001
	Mountain - Edge	0.257	0.125	0.011	0.503	4149.236	2.051	.040
	Thresholded - Edge	-0.012	0.125	-0.257	0.234	4152.414	-0.093	.926
2.25	Grayscale - Edge	0.702	0.125	0.457	0.948	4146.065	5.607	<.001
	Mountain - Edge	0.400	0.125	0.154	0.646	4155.624	3.192	.001
	Thresholded - Edge	-0.074	0.125	-0.320	0.171	4151.783	-0.593	.553

Results Plots

Plot: Preference ~ PreferenceScaling

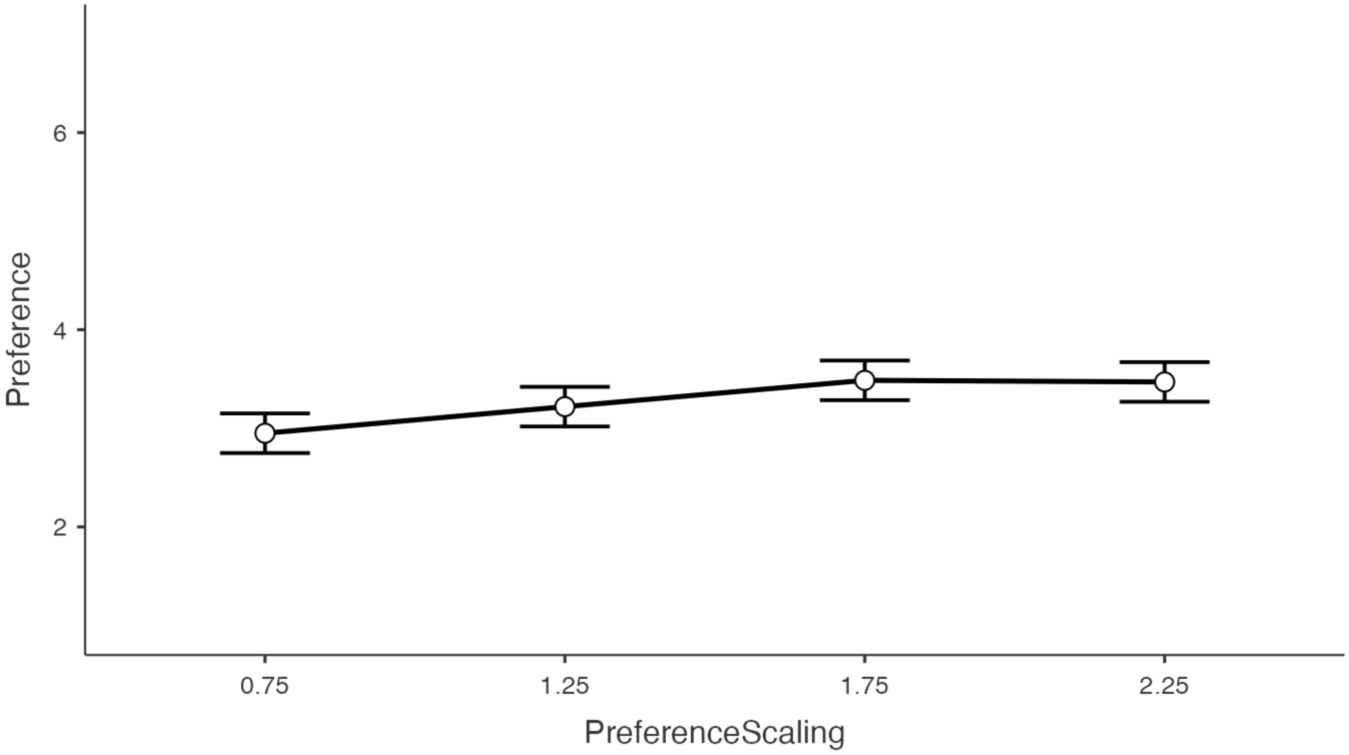

Section S9: Preference Ratings: LME Model with Covariates

Model Info

Info		
Model Type	Mixed Model	Linear Mixed model for continuous y
Model	lmer	Preference ~ 1 + PreferenceScaling + PreferenceImageType + CATI + CHYPS + ASRS + SPQ + PreferenceScaling:PreferenceImageType + PreferenceScaling:CATI + PreferenceScaling:CHYPS + PreferenceScaling:ASRS + PreferenceScaling:SPQ + (1 | ParticipantID)
Distribution	Gaussian	Normal distribution of residuals
Direction	y	Dependend variable scores
Optimizer	bobyqa	
DF method	Satterthwaite	
Sample size	4272	
Converged	yes	
Y transform	none	
C.I. method	Wald	

Note. All covariates are standardized

Model Results

Model Fit

Type	R^2	df	LRT X^2	p
Conditional	0.314	32	1298.128	<.001
Marginal	0.061	31	344.565	<.001

Fixed Effects Omnibus Tests

	F	df	df (res)	p
PreferenceScaling	32.908	3	4156.000	<.001
PreferenceImageType	51.269	3	4156.000	<.001
CATI	0.584	1	84.000	.447
CHYPS	0.075	1	84.000	.785
ASRS	0.432	1	84.000	.513
SPQ	0.471	1	84.000	.495
PreferenceScaling ✻ PreferenceImageType	3.339	9	4156.000	<.001
PreferenceScaling ✻ CATI	3.465	3	4156.000	.016
PreferenceScaling ✻ CHYPS	8.067	3	4156.000	<.001
PreferenceScaling ✻ ASRS	12.190	3	4156.000	<.001
PreferenceScaling ✻ SPQ	1.580	3	4156.000	.192

Parameter Estimates (Fixed coefficients)

				95% Confidence Intervals				
Names	Effect	Estimate	SE	Lower	Upper	df	t	p
(Intercept)	(Intercept)	2.730	0.128	2.479	2.982	268.754	21.300	<.001
PreferenceScaling1	1.25 - 0.75	0.150	0.125	-0.095	0.395	4156.000	1.200	.230
PreferenceScaling2	1.75 - 0.75	0.513	0.125	0.268	0.758	4156.000	4.111	<.001
PreferenceScaling3	2.25 - 0.75	0.483	0.125	0.238	0.728	4156.000	3.871	<.001
PreferenceImageType1	Grayscale - Edge	0.558	0.125	0.313	0.803	4156.000	4.471	<.001
PreferenceImageType2	Mountain - Edge	0.120	0.125	-0.125	0.365	4156.000	0.960	.337
PreferenceImageType3	Thresholded - Edge	0.195	0.125	-0.050	0.439	4156.000	1.560	.119
CATI	CATI	-0.038	0.134	-0.302	0.225	112.981	-0.286	.776
CHYPS	CHYPS	-0.186	0.135	-0.451	0.079	112.981	-1.376	.171
ASRS	ASRS	0.145	0.126	-0.103	0.392	112.981	1.146	.254
SPQ	SPQ	-0.018	0.120	-0.253	0.217	112.981	-0.150	.881
PreferenceScaling1 ✻ PreferenceImageType1	(1.25 - 0.75) ✻ (Grayscale - Edge)	0.326	0.177	-0.020	0.672	4156.000	1.846	.065
PreferenceScaling2 ✻ PreferenceImageType1	(1.75 - 0.75) ✻ (Grayscale - Edge)	0.180	0.177	-0.166	0.526	4156.000	1.018	.309
PreferenceScaling3 ✻ PreferenceImageType1	(2.25 - 0.75) ✻ (Grayscale - Edge)	0.142	0.177	-0.204	0.488	4156.000	0.806	.420
PreferenceScaling1 ✻ PreferenceImageType2	(1.25 - 0.75) ✻ (Mountain - Edge)	-0.045	0.177	-0.391	0.301	4156.000	-0.255	.799
PreferenceScaling2 ✻ PreferenceImageType2	(1.75 - 0.75) ✻ (Mountain - Edge)	0.135	0.177	-0.211	0.481	4156.000	0.764	.445
PreferenceScaling3 ✻ PreferenceImageType2	(2.25 - 0.75) ✻ (Mountain - Edge)	0.288	0.177	-0.058	0.634	4156.000	1.634	.102
PreferenceScaling1 ✻ PreferenceImageType3	(1.25 - 0.75) ✻ (Thresholded - Edge)	0.210	0.177	-0.136	0.556	4156.000	1.188	.235
PreferenceScaling2 ✻ PreferenceImageType3	(1.75 - 0.75) ✻ (Thresholded - Edge)	-0.213	0.177	-0.560	0.133	4156.000	-1.209	.227
PreferenceScaling3 ✻ PreferenceImageType3	(2.25 - 0.75) ✻ (Thresholded - Edge)	-0.277	0.177	-0.623	0.069	4156.000	-1.570	.116
PreferenceScaling1 ✻ CATI	(1.25 - 0.75) ✻ CATI	0.116	0.081	-0.044	0.275	4156.000	1.420	.156
PreferenceScaling2 ✻ CATI	(1.75 - 0.75) ✻ CATI	0.258	0.081	0.099	0.418	4156.000	3.173	.002
PreferenceScaling3 ✻ CATI	(2.25 - 0.75) ✻ CATI	0.160	0.081	0.001	0.320	4156.000	1.970	.049
PreferenceScaling1 ✻ CHYPS	(1.25 - 0.75) ✻ CHYPS	0.022	0.082	-0.138	0.183	4156.000	0.272	.785

Parameter Estimates (Fixed coefficients)

PreferenceScaling2 ✻ CHYPS	(1.75 - 0.75) ✻ CHYPS	0.260	0.082	0.100	0.421	4156.000	3.178	.001
PreferenceScaling3 ✻ CHYPS	(2.25 - 0.75) ✻ CHYPS	0.324	0.082	0.163	0.484	4156.000	3.953	<.001
PreferenceScaling1 ✻ ASRS	(1.25 - 0.75) ✻ ASRS	-0.130	0.077	-0.280	0.020	4156.000	-1.694	.090
PreferenceScaling2 ✻ ASRS	(1.75 - 0.75) ✻ ASRS	-0.363	0.077	-0.513	-0.213	4156.000	-4.749	<.001
PreferenceScaling3 ✻ ASRS	(2.25 - 0.75) ✻ ASRS	-0.393	0.077	-0.543	-0.243	4156.000	-5.136	<.001
PreferenceScaling1 ✻ SPQ	(1.25 - 0.75) ✻ SPQ	-0.137	0.073	-0.279	0.006	4156.000	-1.881	.060
PreferenceScaling2 ✻ SPQ	(1.75 - 0.75) ✻ SPQ	-0.011	0.073	-0.154	0.131	4156.000	-0.153	.879
PreferenceScaling3 ✻ SPQ	(2.25 - 0.75) ✻ SPQ	-0.086	0.073	-0.228	0.057	4156.000	-1.179	.238

Random Components

Groups	Name	Variance	SD	ICC
ParticipantID	(Intercept)	0.769	0.877	0.270
Residual		2.080	1.442	

Note. Number of Obs: 4272 , Number of groups: ParticipantID 89

Simple Effects

ANOVA for Simple Effects of PreferenceImageType

Moderator				
PreferenceScaling	F	Num df	Den df	p
0.75	7.416	3.000	4156.000	<.001
1.25	20.787	3.000	4156.000	<.001
1.75	15.939	3.000	4156.000	<.001
2.25	17.144	3.000	4156.000	<.001

Parameter Estimates for simple effects of PreferenceImageType

Moderator				95% Confidence Intervals				
PreferenceScaling	Effect	Estimate	SE	Lower	Upper	df	t	p
0.75	Grayscale - Edge	0.558	0.125	0.313	0.803	4156.000	4.471	<.001
	Mountain - Edge	0.120	0.125	-0.125	0.365	4156.000	0.960	.337
	Thresholded - Edge	0.195	0.125	-0.050	0.439	4156.000	1.560	.119
1.25	Grayscale - Edge	0.884	0.125	0.639	1.129	4156.000	7.081	<.001
	Mountain - Edge	0.075	0.125	-0.170	0.320	4156.000	0.600	.548
	Thresholded - Edge	0.404	0.125	0.160	0.649	4156.000	3.240	.001
1.75	Grayscale - Edge	0.738	0.125	0.493	0.983	4156.000	5.911	<.001
	Mountain - Edge	0.255	0.125	0.010	0.499	4156.000	2.040	.041
	Thresholded - Edge	-0.019	0.125	-0.263	0.226	4156.000	-0.150	.881
2.25	Grayscale - Edge	0.700	0.125	0.456	0.945	4156.000	5.611	<.001
	Mountain - Edge	0.408	0.125	0.164	0.653	4156.000	3.270	.001
	Thresholded - Edge	-0.082	0.125	-0.327	0.162	4156.000	-0.660	.509

Estimated Marginal Means

Estimate Marginal Means - PreferenceScaling ✻ CATI

					95% Confidence Intervals	
PreferenceScaling	CATI	Mean	SE	df	Lower	Upper
0.75	Mean-1·SD	2.987	0.169	112.981	2.652	3.322
0.75	Mean	2.949	0.103	112.981	2.745	3.152
0.75	Mean+1·SD	2.910	0.169	112.981	2.575	3.245
1.25	Mean-1·SD	3.144	0.169	112.981	2.809	3.479
1.25	Mean	3.221	0.103	112.981	3.017	3.425
1.25	Mean+1·SD	3.298	0.169	112.981	2.963	3.633
1.75	Mean-1·SD	3.267	0.169	112.981	2.932	3.602
1.75	Mean	3.487	0.103	112.981	3.283	3.691
1.75	Mean+1·SD	3.707	0.169	112.981	3.372	4.042
2.25	Mean-1·SD	3.348	0.169	112.981	3.013	3.683
2.25	Mean	3.470	0.103	112.981	3.266	3.674
2.25	Mean+1·SD	3.592	0.169	112.981	3.257	3.927

Estimate Marginal Means - PreferenceScaling ✻ CHYPS

					95% Confidence Intervals	
PreferenceScaling	CHYPS	Mean	SE	df	Lower	Upper
0.75	Mean-1·SD	3.134	0.170	112.981	2.798	3.471
0.75	Mean	2.949	0.103	112.981	2.745	3.152
0.75	Mean+1·SD	2.763	0.170	112.981	2.426	3.099
1.25	Mean-1·SD	3.385	0.170	112.981	3.048	3.721
1.25	Mean	3.221	0.103	112.981	3.017	3.425
1.25	Mean+1·SD	3.057	0.170	112.981	2.721	3.394
1.75	Mean-1·SD	3.412	0.170	112.981	3.076	3.749
1.75	Mean	3.487	0.103	112.981	3.283	3.691
1.75	Mean+1·SD	3.561	0.170	112.981	3.225	3.898
2.25	Mean-1·SD	3.332	0.170	112.981	2.996	3.668
2.25	Mean	3.470	0.103	112.981	3.266	3.674
2.25	Mean+1·SD	3.608	0.170	112.981	3.272	3.944

Estimate Marginal Means - PreferenceScaling ✻ ASRS

					95% Confidence Intervals	
PreferenceScaling	ASRS	Mean	SE	df	Lower	Upper
0.75	Mean-1·SD	2.804	0.163	112.981	2.481	3.126
0.75	Mean	2.949	0.103	112.981	2.745	3.152
0.75	Mean+1·SD	3.093	0.163	112.981	2.771	3.416
1.25	Mean-1·SD	3.206	0.163	112.981	2.883	3.529
1.25	Mean	3.221	0.103	112.981	3.017	3.425
1.25	Mean+1·SD	3.236	0.163	112.981	2.913	3.558
1.75	Mean-1·SD	3.706	0.163	112.981	3.383	4.028
1.75	Mean	3.487	0.103	112.981	3.283	3.691
1.75	Mean+1·SD	3.268	0.163	112.981	2.946	3.591
2.25	Mean-1·SD	3.718	0.163	112.981	3.396	4.041
2.25	Mean	3.470	0.103	112.981	3.266	3.674
2.25	Mean+1·SD	3.222	0.163	112.981	2.899	3.544

Results Plots

Plot: Preference ~ PreferenceScaling ✻ CATI

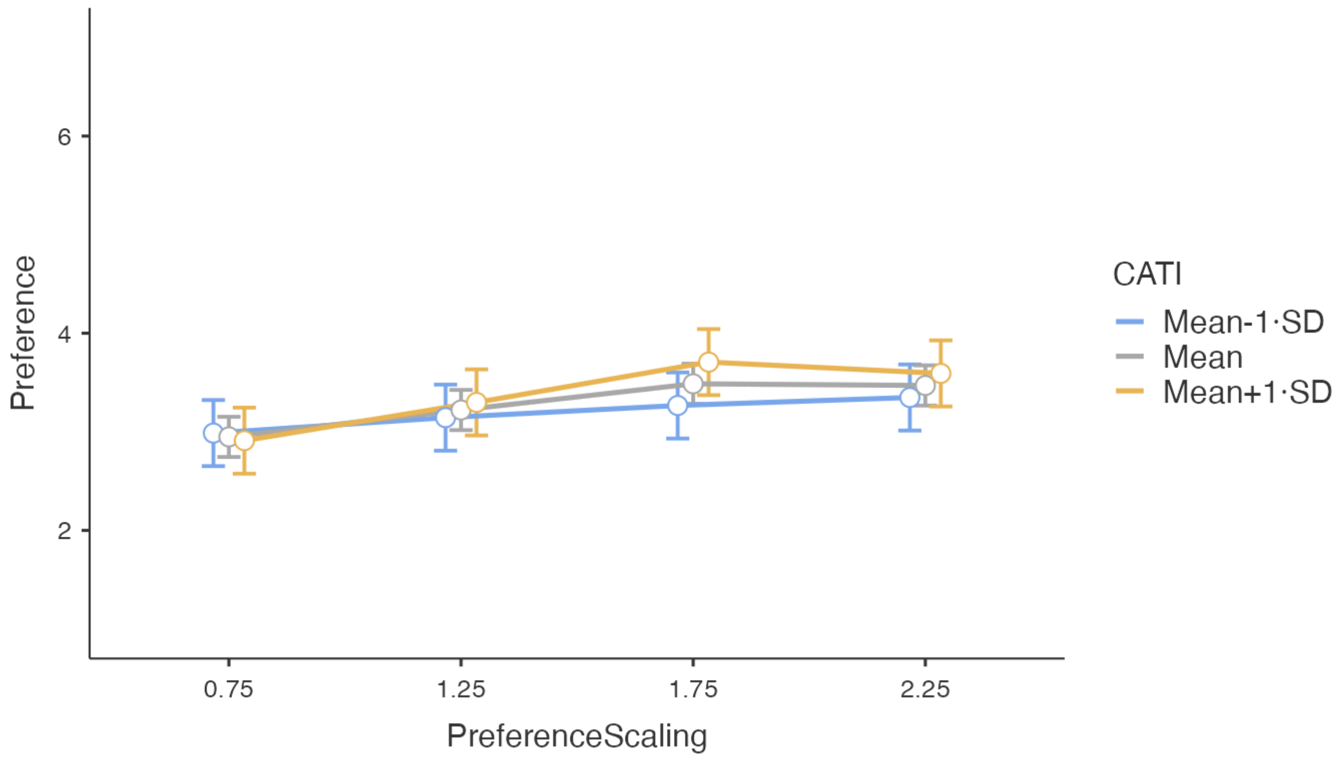

Plot: Preference ~ PreferenceScaling ✻ CHYPS

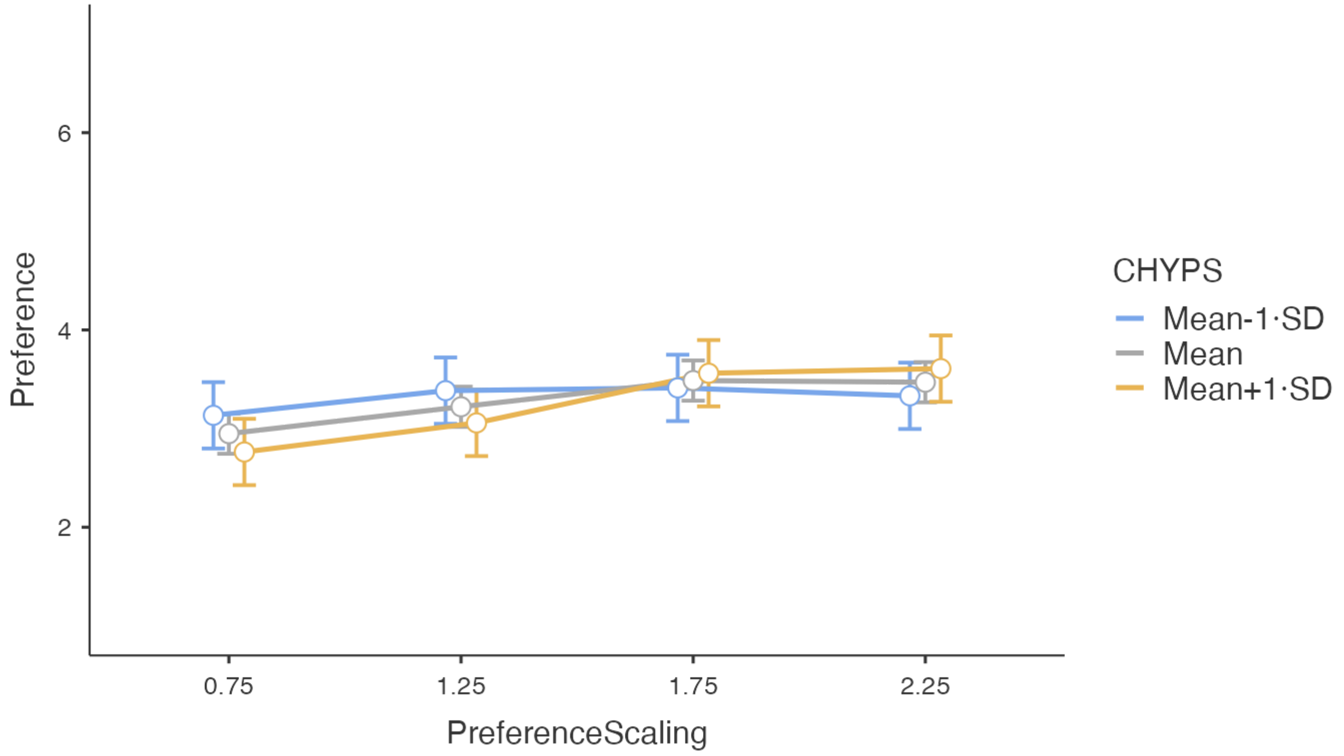

Plot: Preference ~ PreferenceScaling ✻ ASRS

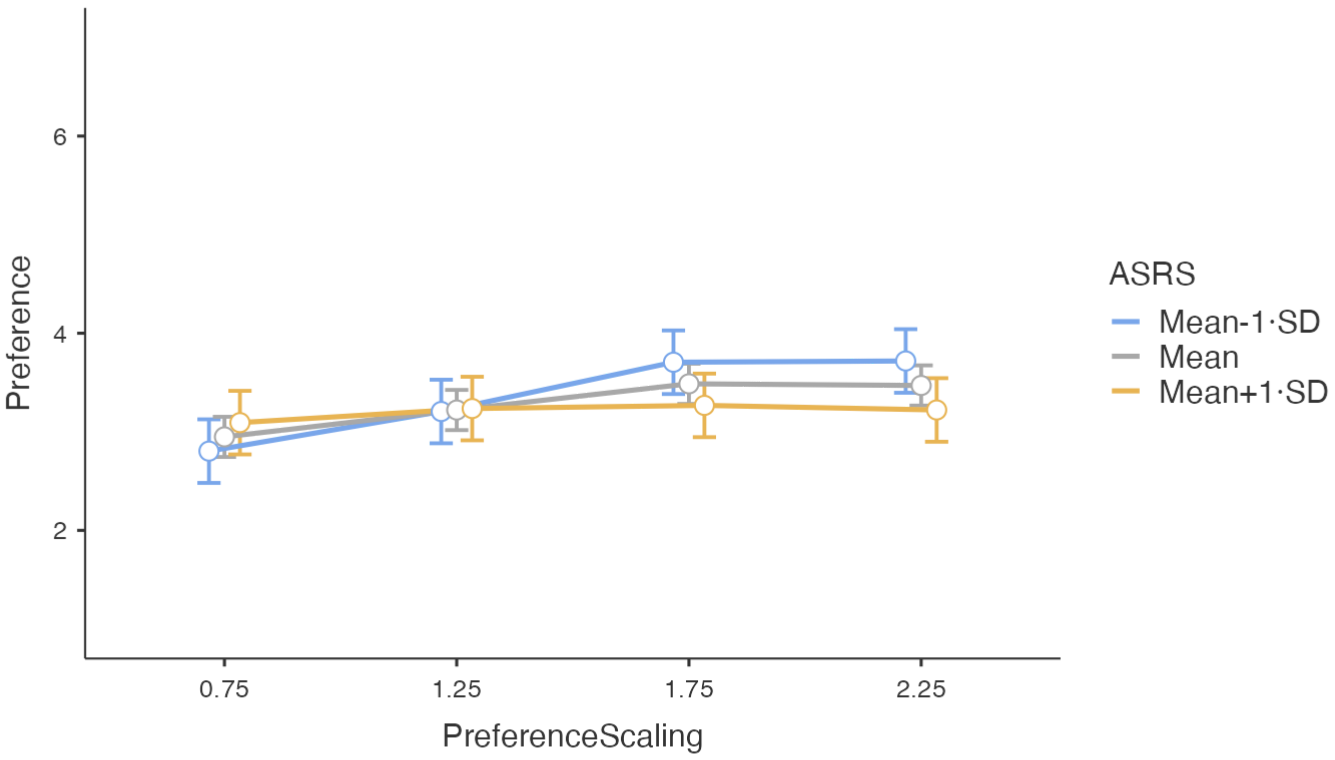
6
4
2
Preference
0.75
1.25
1.75
2.25
PreferenceScaling
ASRS
Mean-1·SD
Mean
Mean+1·SD
